# Efficacy of Short-Course AZT Plus 3TC to Reduce Nevirapine Resistance in the Prevention of Mother-to-Child HIV Transmission: A Randomized Clinical Trial

**DOI:** 10.1371/journal.pmed.1000172

**Published:** 2009-10-27

**Authors:** James A. McIntyre, Mark Hopley, Daya Moodley, Marie Eklund, Glenda E. Gray, David B. Hall, Patrick Robinson, Douglas Mayers, Neil A. Martinson

**Affiliations:** 1 Perinatal HIV Research Unit, University of the Witwatersrand, Johannesburg, South Africa; 2 Boehringer Ingelheim, Johannesburg, South Africa; 3 Department of Obstetrics and Gynaecology, Nelson Mandela School of Medicine, University of KwaZulu Natal, Durban, South Africa; 4 Boehringer Ingelheim Pharmaceuticals, Ridgefield, Connecticut, United States of America; 5 Johns Hopkins University School of Medicine, Baltimore, Maryland, United States of America; National Institute of Child Health and Human Development, United States of America

## Abstract

**Background:**

Single-dose nevirapine (sdNVP)—which prevents mother-to-child transmission of HIV—selects non-nucleoside reverse-transcriptase inhibitor (NNRTI) resistance mutations in the majority of women and HIV-infected infants receiving it. This open-label, randomised trial examined the efficacy of short-course zidovudine (AZT) and lamivudine (3TC) with sdNVP in reducing NNRTI resistance in mothers, and as a secondary objective, in infants, in a setting where sdNVP was standard-of-care.

**Methods and Findings:**

sdNVP alone, administered at the onset of labour and to the infant, was compared to sdNVP with AZT plus 3TC, given as combivir (CBV) for 4 (NVP/CBV4) or 7 (NVP/CBV7) days, initiated simultaneously with sdNVP in labour; their newborns received the same regimens. Women were randomised 1∶1∶1. HIV-1 resistance was assessed by population sequencing at: baseline, 2, and 6 wk after birth. An unplanned interim analysis resulted in early stopping of the sdNVP arm. 406 pregnant women were randomised and took study medication (sdNVP 74, NVP/CBV4 164, and NVP/CBV7 168). HIV-1 resistance mutations emerged in 59.2%, 11.7%, and 7.3% of women in the sdNVP, NVP/CBV4, and NVP/CBV7 arms by 6 wk postpartum; differences between NVP-only and both NVP/CBV arms were significant (*p*<0.0001), but the difference between NVP/CBV4 and NVP/CBV7 was not (*p* = 0.27). Estimated efficacy comparing combined CBV arms with sdNVP was 85.6%. Similar resistance reductions were seen in infants who were HIV-infected by their 6-wk visit.

**Conclusions:**

A short course of AZT plus 3TC, supplementing maternal and infant sdNVP, reduces emergent NNRTI resistance mutations in both mothers and their infants. However, this trial was not powered to detect small differences between the CBV arms.

**Trial registration:**

www.ClinicalTrials.gov NCT 00144183

*Please see later in the article for the Editors' Summary*

## Abstract 2

Neil Martinson and colleagues report a randomized trial of adding short-course zidovudine+lamivudine to reduce drug resistance from single-dose nevirapine used to prevent mother-to-child transmission of HIV.

## Editors' Summary

**Background:**

Currently, about 33 million people are infected with the human immunodeficiency virus (HIV), which causes AIDS. HIV can be treated with combination antiretroviral therapy (ART), commonly three individual antiretroviral drugs that together efficiently suppress the replication of the virus. HIV infection of a child by an HIV-positive mother during pregnancy, labor, delivery, or breastfeeding is called mother-to-child transmission (MTCT). In 2007, an estimated 420,000 children were newly infected with HIV, the majority through MTCT. Most of these mothers and children live in sub-Saharan Africa where child and maternal mortality rates are high and mortality in HIV-infected children is extremely high. MTCT is preventable and there is a global commitment, agreed at the UN General Assembly Session on HIV/AIDS in 2001, to reduce the proportion of infants infected with HIV by 50% by 2010.

**Why Was This Study Done?:**

In many resource-limited settings, MTCT is prevented by giving a single dose of nevirapine (an antiretroviral drug which has a long duration in the body and protects the fetus during labor and delivery only) to HIV-infected women in labor and also to a baby within 72 hours of birth. However, nevirapine, a non-nucleoside reverse-transcriptase inhibitor (NNRTI), which suppresses the replication of the virus, is associated with increased resistance of HIV, in mother and child, to NNRTI. This resistance reduces the effectiveness of future treatments of both mother and child with combination ART that includes an NNRTI; such regimens are the mainstay for long-term treatment of HIV in developing countries. The researchers investigated whether giving other antiretroviral drugs with nevirapine, during labor and delivery, to both mother and her newborn reduced the chances of them developing resistance to NNRTIs.

**What Did the Researchers Do and Find?:**

The researchers selected 406 HIV-positive pregnant women for study across five sites in South Africa between February 2003 and May 2007. The women and their newborn babies were randomly assigned to receive, either (i) a single dose of nevirapine, (ii) a single dose of nevirapine plus combivir (zidovudine combined with lamivudine) for four days, or (iii) a single dose of nevirapine plus combivir for seven days. At two days, two weeks, and six weeks after delivery blood was collected from mothers and babies. HIV virus from blood samples was analyzed for resistance mutations, and mothers and children with resistance mutations were monitored for a further 96 weeks until no resistance was detected or combination ART (also called “HAART”) was started. Enrollment into the single-dose nevirapine arm was stopped early because a very high rate of NNRTI resistance mutations was found and other investigators reported long-term bad consequences of NNRTI-resistance on subsequent ART. The two nevirapine plus combivir arms were continued. The researchers found that selection of resistance mutations by single-dose nevirapine was reduced in mother and child by the addition of zidovudine and lamivudine for a short period; resistance mutations were found in 59.2% of women who got nevirapine only but only 11.7%, and 7.3% of women treated nevirapine plus four days combivir, and nevirapine plus seven days combivir respectively. A reduction was also seen in new NNRTI resistant mutations in the HIV-infected infants that received combivir. The study did not have enough women to show that there was a real difference between the resistance in the four-day and seven-day combivir regimens.

**What Do These Findings Mean?:**

These findings show that a short-course treatment of zidovudine and lamivudine in addition to a single dose of nevirapine during labor and birth reduces the selection of NNRTI resistance mutations in both mother and child. The drug regimens appeared safe, and easy to provide and adhere to. Preliminary results from this study contributed to a change in clinical practice for the care of pregnant women with HIV; in 2004 the World Health Organisation guidelines introduced a short course of combivir with nevirapine for the management of pregnant HIV-infected women. However, the study had some limitations. It used HIV-positive women who were mainly infected with a subtype of HIV called HIV-1 clade C and who had a lot of virus in their blood. NNRTI resistance after treatment with nevirapine is more common in clade C than in others and this study does not address the effect of these combinations for preventing NNRTI resistance in other HIV subtypes. Also, World Health Organization, national, and international guidelines recommend combination ART during pregnancy, as it decreases HIV transmission from mother to child in the uterus to <2% in resource-limited settings. Although long-term combination treatment may not be available in all locations, this study does not tell us how the short-term combinations during and after delivery tested would compare to longer-term combinations given to pregnant women in reducing both HIV transmission and HIV drug resistance.

**Additional Information:**

Please access these Web sites via the online version of this summary at http://dx.doi.org/10.1371/journal.pmed.1000172.

- This study is further discussed in a *PLoS Medicine* Perspective by Lehman et al.
- The US Centers for Disease Control and Prevention provide information for HIV treatment and prevention
- MedlinePlus provides extensive information on symptoms and treatment for HIV/AIDS as well as access to related clinical trials and medical literature
- aidsmap, a nonprofit, nongovernmental organization provides information on HIV and supporting those living with HIV
- The World Health Organization gives information on the prevention of mother-to-child transmission of HIV

## Introduction

Single-dose nevirapine (sdNVP) given to HIV-1 infected women in labour, followed by a single dose administered to their infants within 72 h of birth, is the simplest regimen to prevent intrapartum mother-to-child transmission (MTCT) of HIV-1 [1]–[3] and is used extensively in resource-limited settings. However, NVP has a long half-life, leading to detectable plasma levels for as long as 3 wk following a single dose [4],[5] and is associated with selection of non-nucleoside reverse-transcriptase inhibitor (NNRTI) resistance mutations detectable with population sequencing in 19%–75% of exposed mothers and 33%–87% of their infected infants [6]–[10]. Selection of NNRTI-resistance mutations has also been described in 17%–45% of mothers who received sdNVP with a short course of antenatal zidovudine (AZT) [11],[12]. The presence of these mutations may reduce the clinical response to NNRTI-based combination antiretroviral therapy (HAART) in mothers and infants, especially if initiated less than 6 mo after exposure to sdNVP [11],[13]. In October 2008, the US National Institutes of Health (NIH) released a press statement reporting that a randomised trial comparing the efficacy of NVP-based HAART to ritonavir-boosted-lopinavir HAART in women previously exposed to sdNVP was terminated early because of higher rates of virological failure and death in the NVP-based HAART arm; this finding was accentuated in women with NVP-resistant HIV (http://www3.niaid.nih.gov/news/newsreleases/2008/ACTG_5208.htm).

We hypothesised that adding other antiretroviral drugs during the period of high selection pressure, after exposure to sdNVP, may prevent the selection of maternal-resistant HIV-1 variants after exposure to sdNVP. The primary objective of this trial—the Treatment Options Preservation Study (TOPS)—was to compare the selection of maternal NNRTI resistance mutations after receipt of three NVP-containing regimens for prevention of mother-to-child transmission (PMTCT): sdNVP alone or sdNVP combined with short courses (either 4 or 7 d) of AZT and lamivudine (3TC), given as combivir, initiated simultaneously with the dose of NVP at the onset of labour to mother and continued postpartum. Our secondary objective was to compare rates of resistance mutations in infants who received the same study drugs as their mothers but as individual component suspensions.

## Methods

### Study Setting and Design

This was an open-label, randomised controlled study conducted between February 2003 and May 2007 at five sites in South Africa (listed in the Acknowledgments) where the standard of care for the PMTCT of HIV-1 at the time of the trial was sdNVP—the HIVNET 012 regimen given to mothers at the start of labour and to neonates within 72 h of birth [1]. At the time of enrolment into the study HAART was not routinely available for pregnant women as treatment for their own HIV disease. Pregnant women were assessed at two screening visits after 36 wk of gestation. The late gestational age at recruitment was chosen to ensure that most complicated pregnancies including premature labour and delivery had revealed themselves by then. Pregnant HIV-infected women were eligible for randomisation if they were ≥18 y, had plasma HIV viral loads >2,000 RNA copies/ml, were antiretroviral drug naïve, did not have any medical contraindications to NVP, AZT, or 3TC, and gave written informed consent. The viral load criterion was to ensure that a baseline genotype could be performed. Haemoglobin <7.5 g/dl and elective caesarean section were exclusion criteria. Elective caesarean section is not routine for HIV-infected women in the South African public sector and indications for elective caesarean section would likely be for an obstetric or medical complication. According to the original protocol (Text S1), eligible women who had consented antenatally were randomly assigned at the onset of labour to receive sdNVP alone or sdNVP with 4 (NVP/CBV4) or 7 (NVP/CBV7) d of AZT with 3TC initiated at the time of NVP dosing and continued postpartum. A randomisation sequence maintaining a 1∶1∶1 balance between treatment arms was generated by the data management centre and distributed in blocks of six, on blinded scratch cards to trial sites. Newborns were assigned the same treatment arm as their mothers. Infant feeding decisions were left to the mothers following infant feeding counselling [14]. Women who had provided consent for the study but did not meet the randomisation criteria received the then standard of care—sdNVP—as did their infants.

All pregnant women were given an observed dose of 200 mg NVP orally when in labour. CBV (AZT 300 mg+3TC 150 mg) dosing was one tablet every 12 h, initiated with NVP. To ensure that mislaid tablets did not result in poor adherence, mothers in the NVP/CBV4 and the NVP/CBV7 arms were dispensed 12 and 18 CBV tablets representing a total possible course of 6.5 and 9.5 d, respectively. Women were advised to take them 12 hourly to complete either 4 or 7 d. Adherence was checked by pill count at 2 wk postpartum. Infants received a sdNVP suspension (2 mg/kg) and simultaneously commenced AZT oral suspension (Retrovir) 12 mg (1.2 ml) and 3TC oral suspension 6 mg (0.6 ml) every 12 h. Mothers were requested to give medication to their infants to complete either 4 or 7 d of therapy. Infant compliance was checked by comparing bottle-weight prior to initial dispensing with bottle-weight at the 2-wk postnatal visit, measured by trained study staff using study-specific calibrated electronic scales.

### Follow-up of Women and Children

Mothers and infants were reassessed at 2 d, 2 wk, and 6 wk following delivery. Whole blood specimens were collected for plasma quantitative HIV-1 RNA assay (HIV Amplicor monitor 1.5, Roche Molecular Systems) and HIV-1 genotyping (TruGene HIV-1 genotyping kit and OpenGene DNA sequencing system, Bayer). The limit of detection of the quantitative HIV RNA assay was 400 copies/ml. All mothers and/or infants who had resistance-associated mutations at their 2- and/or 6-wk visit were followed up at 12, 24, 36, 48, 72, and 96 wk or until no resistance was detected or until HAART was initiated. The study laboratory was blinded to treatment allocation.

Data management and adverse event reporting were coordinated by the data management centre at Boehringer Ingelheim, Randburg, South Africa. The trial was approved by the South African Medicines Control Council and ethics committees of the Universities of the Witwatersrand, Pretoria, Kwa-Zulu Natal, and the Pharma-Ethics Independent Research Ethics Committee. The trial design was registered in the ClinicalTrials.gov register: NCT00144183.

### Statistical Analysis

The initial sample size of 80 mothers per treatment arm had a power of 80% to detect a difference with an alpha of 0.05 (two sided) if either CBV treatment arm decreased emergent maternal resistance from 20% to 4%. The sample size included an expected rate of new NNRTI resistance mutations in the NVP-only arm of 20%–30%, on the basis of data from the HIVNET 012 study in which HIV-1 clades A or D predominated [7]. However, data describing selection of resistance at double this rate in HIV-1 clade C-infected women from South Africa exposed to sdNVP were presented by NAM at the 11th Conference of Retroviruses and Opportunistic Infections (CROI) in San Francisco in 2004. These data, in conjunction with the potential long term consequence of NNRTI resistance [11], resulted in an investigator-driven unplanned interim analysis (at the request of JMc of the Perinatal HIV Research Unit and performed by the sponsor) in mid-2004 as there was no data safety management board (DSMB) for this study. The rate of NNRTI resistance mutations in the NVP-only arm postpartum was found to have exceeded 50%, so enrolment into the NVP-only arm was halted after discussions with a representative of the Medicines Control Council and the Chair of the local ethics committee.

An amended protocol was then used (see Text S1, S2, S3, S4, S5, S6, S7, S8 for protocol amendments); enrolment was continued into the NVP/CBV4 and NVP/CBV7 arms only, using the original randomisation schedule but rerandomising if the NVP arm appeared. The sample size was increased to 150 mother infant pairs per arm (Text 7 Amendment 6); assuming 15% resistance at wk 6 in the less effective arm with the more effective arm expected to decrease resistance to 5%. The revised sample size of 150 per treatment arm had a power of 80% to detect a difference with an alpha of 0.05 (two sided) if one of the CBV treatment arms decreased maternal resistance from an expected 15% to 5%. We report results of all participants (mothers and infants) in all three arms.

A mother or infant was classified as having new or emergent NNRTI resistance-associated mutations if an amino acid substitution was observed during follow-up, at either week 2 or week 6 or both, at one of the following reverse transcriptase (RT) codons: 98G, 100I, 101E/P, 103N/S, 106A/I/M, 108I, 179D/E, 181C/I/V, 188C/H/L, 190A/C/E/Q/S, 225H, 227L, 230L, 236L, 238N/T, 318F. For the purposes of this study we did not differentiate between “high level” and other NNRTI resistance mutations. Likewise, nucleoside analogue reverse transcriptase (NRTI) resistance was identified on the basis of one or more amino acid substitutions 41L, 44A/D, 62V, 65R, 67N/G, 69D/N/X, 70R, 74IV, 75AI/M/T, 77L, 115F, 116Y, 118I, 151M, 161L, 184I/V, 210W, 215F/Y, 219E/N/Q (Stanford University HIV database, http://hivdb.stanford.edu/index.html, accessed from 2000 to 2007).

The initial primary analysis was three pair-wise comparisons (NVP-only versus NVP/CBV4, NVP-only versus NVP/CBV7, and NVP/CBV4 versus NVP/CBV7) of the proportion of mothers with new NNRTI resistance-associated mutations by week 6 between the three treatment arms using Fisher's exact test. Estimated efficacy was defined for women who received a CBV regimen as the proportion of mothers for whom emergent resistance-associated mutations were prevented [15] combining all CBV recipients into one group. A Cochran-Armitage test for trend was used to assess ordered categorical variables.

Maternal secondary endpoints in this study included adverse events, changes in maternal viral load, and the effect of baseline CD4 on the development of resistance. Infant-related endpoints were the percentage of infants infected with HIV-1, with or without new NNRTI resistance-associated mutations. Infected infants were classified as having acquired their HIV-1 infection either intrauterine or intra/postpartum on the basis of the results of DNA PCR testing [16].

## Results

**Figure 1:**
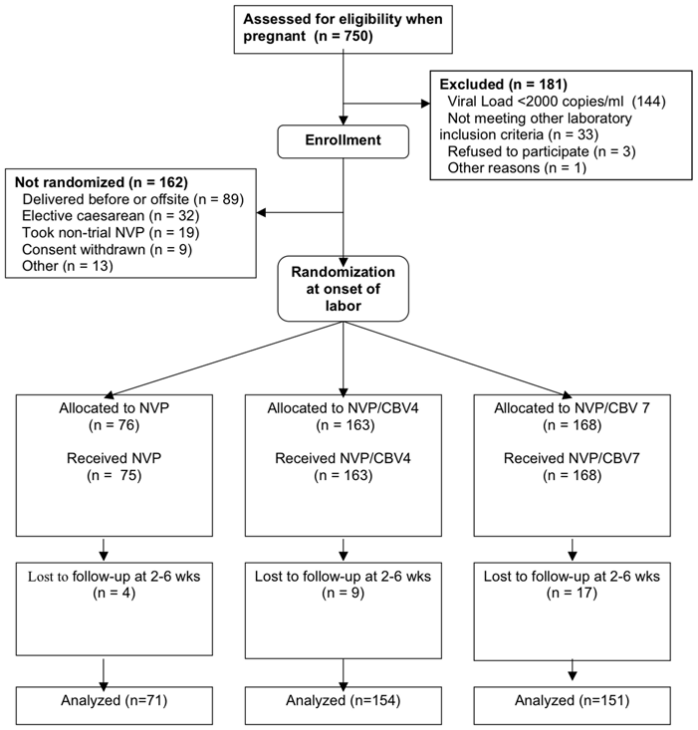
CONSORT diagram of the trial. Screening was while women were pregnant and randomisation was during labour (CBV, 3TC with AZT).

750 pregnant women were screened for inclusion into the trial (Figure 1) starting in February 2003, of whom 181 (24%) did not meet the entry criteria, the majority (144) of this group because their viral load was below 2,000 copies/ml. 162 women were not randomised during labour for the following reasons: they either delivered before randomisation or not at the study site (89), had elective caesarean section (32), or received nontrial NVP (19). 407 women were randomised from March 2003 to November 2005, 406 of whom took trial medication. Their median age was 27 y (interquartile range [IQR] 24–30), median CD4 count was 318 cells/mm^3^ (IQR 212–425), and median screening HIV RNA viral load was 27,100 copies/ml (IQR 8,130–74,200) (Table 1). There were 376 mothers (92.4% of randomised) with follow-up information at 6 wk; no participants were omitted from the 6-wk analysis other than those lost to follow-up or with no postpartum genotypic results. Four NVP-only, four NVP/CBV4, and six NVP/CBV7 did not return for any follow up visits; their viral load and CD4 counts were similar to the women that did return. There was no sequencing data on another 17 women, all of whom were in the CBV-containing arms; 14 had viral loads <400 copies/ml and the samples provided by the remainder were either insufficient or unable to be amplified. There were 413 live births and two stillbirths. Among the live births, there were eight sets of twins. One intrauterine death was detected after randomisation but before receipt of treatment, leading to the mother not being treated. Two infants were unable to take study medication because of birth asphyxia (both in the NVP/CBV4 arm). The 411 infants who received medication had a median weight of 3.0 kg (IQR 2.9–3.4 kg) and a median length of 50 cm (IQR 48–52 cm); 48.9% were female (Table 1). HIV-1 clade C was detected in 98.3% (399/406) of pregnant women, and 12 (3%; 12/393; 95% confidence interval [CI] 2%–5%) had an NNRTI resistance-associated mutation immediately prior to receipt of NVP; one woman in the NVP-only arm, eight in the NVP/CBV4 arm, and three in the NVP/CBV7 arm. Of these women, 11 had one NNRTI resistance-associated mutation (two A98G, two V101E, two K103N, one V106IM, one 108I, two V179D, one M230L) and one had V106M, Y181C, and G190A. Sequencing for resistant mutations immediately prior to receipt of NVP did not yield a result in 14 mothers (3.4% of mothers tested), their median viral load was 539 copies/ml (IQR<400–17,300 copies/ml).

**Table 1:** Baseline characteristics of randomised HIV-infected women and those of their infants at birth, by study arm.

Maternal and Infant Characteristics	NVP, *n* = 75	NVP/CBV4, *n* = 163	NVP/CBV7, *n* = 168	All, *n* = 406
**Maternal characteristics at enrolment**				
Black African	75 (100%)	161 (98.8%)	168 (100%)	404 (99.5%)
Median age in years (IQR)	26 (23–30)	27 (24–31)	26 (23–30)	27 (24–30)
Mean weight in kg (SD)	74.5 (13.6)	75.3 (12.9)	77.8 (14.4)	76.2 (13.7)
Infected with HIV subtype C	74 (98.7%)	159 (97.5%)	166 (98.8%)	399 (98.3%)
Median viral load in copies/ml (IQR)	27,900 (6,800–79,200)	23,950 (6,315–77,950)	30,100 (11,300–67,200)	27,100 (8,130–74,200)
Median CD4 count in cells/mm^3^ (IQR)	347 (198–434)	309 (211– 408)	331 (233–423)	318 (212–425)
Emergency caesarean section	17 (22.7%)	31 (19.0%)	49 (29.2%)	97 (23.9%)
**Infant characteristics at birth**				
Live births	77	164	170	411
Gender - girls	39 (50.6%)	80 (48.8%)	82 (48.2%)	201 (48.9%)
Median weight kg (IQR)	3.0 (2.9, 3.3)	3.0 (2.9, 3.4)	3.0 (3.0, 3.5)	3.0 (2.9, 3.4)
Median length cm (IQR)	50 (48, 51)	50 (48, 52)	51 (48, 53)	50 (48, 52)

CBV, AZT plus 3TC administered to mothers and their new born infants for either 4 or 7 d; NVP, sdNVP administered to mothers and their new born infant; SD, standard deviation.

### Selection of Maternal NNRTI Resistance Mutations

The NVP-only, NVP/CBV4, and NVP/CBV7 regimens were administered to 75, 163, and 168 women, respectively, of whom 71 (95%), 154 (95%), and 151 (90%) had follow-up visits and sequence results at 2 and 6 wk postnatally. Only eight of all the resistance mutations looked for were able to be sequenced in the postnatal specimens (Table 2); the remaining were either present at proportions below the limit of detection or were not present at all. New NNRTI resistance-associated mutations were detected at 2 wk, at 6 wk, or at both visits in 59.2% (42/71; 95% CI 46.8%–70.7%), 9.7% (15/154; 95% CI 5.1%–14.4%), and 7.3% (11/151; CI 3.7%–12.7%) of these women, respectively.

**Table 2:** New resistance mutations by study arm with estimated efficacy of preventing NNRTI resistance mutations in women who received either the 4 or 7 d of AZT plus 3TC (CBV4/7) treatment arms compared to those exposed to sdNVP alone.

Study Arm	NNRTI Resistance Mutation Codon										Women with New NNRTI Resistance Mutations (95% CI)
	98	101	103	106I	106A	106M	108	181	188	190	
**NVP, ** ***n*** ** = 71**	1 (1.4%)	0	31 (43.7%)	21 (29.6%)	10 (14.1%)	13 (18.3%)	0	28 (39.4%)	19 (26.8%)	14 (19.7%)	42
											59.2% (46.8–70.7)
**NVP/CBV4, ** ***n*** ** = 154**	0	0	7 (4.5%)	8 (5.2%)	0	8 (5.2%)	1 (0.6%	4 (2.6%)	4 (2.6%)	3 (1.9%)	15
											9.7% (5.1–14.4)
**NVP/CBV7, ** ***n*** ** = 151**	1 (0.7%)	3 (2.0%)	4 (2.6%)	3 (2.0%)	1 (0.7%)	2 (1.3%)	0	4 (2.6%)	1 (0.7%)	4 (2.6%)	11
											7.3% (3.7–12.7)
**Combined CBV arms, ** ***n*** ** = 305**	1 (0.3%)	3 (1.0%)	11 (3.6%)	11 (3.6%)	1 (0.3%)	10 (3.3%)	1 (0.3%	8 (2.6%)	5 (1.6%)	7 (2.3%)	26
											8.5% (5.6–12.2)
**Estimated efficacy** a **(95% CI)**	—	—	91.7% (84.5–95.6)	87.8% (76.1–93.8)	97.7% (86.1–99.6)	82.1% (61.2–91.6)	—	93.3% (86.2–96.8)	93.9% (84.6–97.6)	88.4% (72.8–95.0)	85.6% (78.2–90.5)^b^

a Estimated efficacy was calculated by comparing the expected rate of new resistance mutations based on the actuals in the NVP arm with the observed rate in the combined CBV arms. For codons where the number was ≤1, no efficacy calculation was done.

b Overall efficacy.

Most mothers receiving CBV took more than the prescribed number of doses as they all were dispensed two additional days of study drug; in the NVP/CBV4 treatment arm women took a median of nine doses (IQR 8–11), their median maternal CBV adherence was 113% (IQR 100%–133%); and in the NVP/CBV7 treatment arm women took a median of 15 doses (IQR 14–17), their adherence was 107% (IQR 100%–114%). However, when women with CBV adherence of <75% or >125% are excluded, resistance rates remain unchanged compared to those including these women: 10.7% (11/103; CI 6%–18%) for NVP/CBV4 and 6.3% (9/144; CI 3%–11%) for NVP/CBV7. An as-treated analysis, restricted to women who received CBV, suggested no relationship between the number of doses of CBV taken and the selection of resistance (permutation test, *p* = 0.3305).

On the basis of the 59.2% rate of resistance emergence in the NVP-only arm, the expected number of mothers with emergent resistance in the NVP/CBV4 arm and NVP/CBV7 arm was estimated at 92 and 91, respectively. The observed rates of emergence correspond to 80.5% and 87.8% efficacy in preventing emergence of resistance, respectively. Comparing the NVP arm to the combined CBV arms the estimated efficacy is 85.6%. Mutation-specific efficacy was over 90% for both leading mutations: K103N and Y181C.

The pattern of emergent mutations was altered by use of CBV (Table 2): 106M was the second leading mutation with the selection of relatively uncommon mutations at 101, and 108 which were not seen with NVP alone. Furthermore, after NVP-only, 60% (25/42; CI 43%–74%) of the mothers with mutations at 2 and/or 6 wk had three or more different NNRTI mutations. Whereas, 19% (5/26; 95% CI 7%–39%) of those with mutations after NVP/CBV4 or NVP/CBV7 had three or more different NNRTI mutations (Cochran-Armitage trend test, *p* = 0.001). One woman in the NVP/CBV7 arm had a new mutation associated with 3TC resistance at RT codon 184, detected by population sequencing at week 6 but not at week 12. This was the only nucleoside analogue reverse-transcriptase resistance mutation detected.

After the 6-wk visit, follow-up of women with resistance mutations continued until NNRTI mutation-free virus was documented. The last study follow up visit was in January 2007. Fading of NNRTI resistance mutations appeared to occur more rapidly in women who received NVP/CBV7 than NVP/CBV4 (Table 3). At 12 wk postpartum, 62% of NVP-only women who had NNRTI resistance mutations at 6 wk retained their NNRTI resistance, whereas 34% of the women who received one of the NVP/CBV regimens and who had NNRTI resistance at 6 wk retained resistance at 12 wk (Fisher's exact test, *p* = 0.04). At 24 and 48 wk the results are similar, but there were few women at these two time points. 

**Table 3:** Long-term follow-up of women with emergent NNRTI mutations detected 2–6 wk after exposure to sdNVP during labour.

Arm	Resistance and Visit Outcome	Weeks after delivery		
		12	24	48
**NVP 42/71 women with emergent mutations at 6 wk**	No resistance detected	11	24	31
	Resistance mutation(s) detected	26	13	5
	Total women tested at that visit	37	37	36
	Resistance mutation(s) detected at prior visit but lost to follow up at this visit	5	5	6
	Total potentially with resistance mutations	31 (43.7%)	18 (25.4%	11 (15.5%
**NVP/CBV4 18/154 women with emergent mutations at 6 wk**	No resistance detected	6	9	6
	Resistance mutation(s) detected	2	1	1
	Total women tested at that visit	8	10	7
	Resistance mutation(s) detected at prior visit but lost to follow up at this visit	7	5	5
	Total potentially with resistance mutations	9 (5.8%)	6 (3.9%)	6 (3.9%)
**NVP/CBV7 11/151 women with emergent mutations at 6 wk**	No resistance detected	4	9	9
	Resistance mutation(s) detected	5	1	0
	Total women tested	9	10	9
	Resistance mutation(s) detected at prior visit but lost to follow up at this visit	2	1	2
	Total potentially with resistance mutations	7 (4.6%)	2 (1.3%)	2 (1.3%)

### Maternal Viral Load

The median maternal viral load in labour, prior to receipt of study medication, was 27,900 copies/ml, 23,950 copies/ml, and 30,100 copies/ml for the three treatment arms, NVP, NVP/CBV4, and NVP/CBV7, respectively. HIV RNA was modestly reduced by day 2 and reached an observed median nadir at day 14 with counts of 7,650 copies/ml for NVP-only, 421 copies/ml for NVP/CBV4, and <400 copies/ml for NVP/CBV7. However, by the 6-wk visit median maternal HIV RNA levels were similar to those at baseline: 33,600 copies/ml, 28,600 copies/ml, and 33,850 copies/ml for the NVP-only, NVP/CBV4, and NVP/CBV7 arms, respectively.

### Maternal Baseline CD4 and Emergent NNRTI Resistance

One-fifth of the women recruited into this trial (21%, 85/406, 95% CI 17%–25%) had CD4 cell counts of ≤200 cells/µl. We compared emergent resistance rates by whether baseline CD4 was ≤200 or >200 cells/µl using the Fisher's exact, two-sided test: NVP arm, 82.4% (14/17) versus 48% (24/50), *p* = 0.02; NVP/CBV4 arm, 25.8% (8/31) versus 5.5% (6/110), *p* = 0.003; NVP/CBV7 arm, 10.3% (3/29) versus 5.4% (6/112), *p* = 0.39.

### Efficacy in Preventing MTCT

The overall, combined intra-uterine and peri/postpartum HIV transmission rate by the 6-wk visit was 12.2% (49/403; CI 9.3%–15.7%). The majority of infants with HIV-1 infection were HIV-1 positive when first tested within a day of delivery (HIV DNA- and RNA-positive) and confirmed-infected at follow-up (43/403 infants; 8/76 NVP-only, 25/161 NVP/CBV4, 10/166 NVP/CBV7), suggesting in utero HIV acquisition. Three additional infants were HIV-1 infected when first tested at 2–6 wk of age, all in the NVP/CBV7 arm and three were HIV-negative initially, but positive at 6 wk.

### Infants Developing NNRTI-Resistant Mutations

In the infants with intra-uterine HIV-1 infections and follow-up after exposure to antiretrovirals, NNRTI-resistant mutations were seen in seven of the eight HIV-infected infants treated with NVP-only, four of 25 of those who received NVP/CBV4, and none of the ten who received NVP/CBV7. The difference between NVP alone and the combined NVP/CBV arms is statistically significant (Fisher's exact, two-sided, *p*<0.001). No mother-infant pair developed identical NNRTI-resistant mutations.

### Maternal Safety

Three maternal deaths occurred and all received NVP/CBV4. The deaths occurred at 4, 9, and 12 mo after delivery and all were caused by respiratory disease; tuberculosis was suspected in the deaths of two of the three women. Both of the participants who died of suspected TB had CD4 counts of <100 cells/mm^3^ at the time of screening, one woman had been treated for tuberculosis for 4 mo prior to her death.

During the antenatal screening period, before any study drug had been administered, one-third of the mothers suffered adverse events. The most common events during this period were urinary tract infections. Pregnancy-induced hypertension occurred in ten (2.5%) mothers, two of whom were diagnosed with pre-eclampsia. Following randomisation and delivery the most common adverse events were also infections: 4% (3/70), 6.7% (11/163), and 4.2% (7/168) for the NVP-only, NVP/CBV4, and NVP/CBV7 arms, respectively. Postpartum sepsis was diagnosed in 1.3% (1/70), 3.1% (5/163), and 2.4% (4/168) for the NVP-only, NVP/CBV4, and NVP/CBV7 arms, respectively. Suspected adverse drug reactions were reported in 2.5% (10/406) of mothers and were limited to the combination arms, 1.8% in NVP/CBV4 arm (3/163), and 4.2% in NVP/CBV7 arm (7/168); none were severe. Rashes were reported for six women, for one of them a relationship to NVP use was considered possible. The most common abnormal laboratory tests were below normal haemoglobin and above normal aspartate aminotransferase (AST) levels; there were 24, 55, and 56 women in the NVP, NVP/CBV4, and NVP/CBV7 arms, respectively, whose AST levels were elevated above normal. However, there were no hepatic adverse events reported. There were no significant trends associated with CBV use.

Serious adverse events were observed in 4.9% of mothers during the screening period and in 8.1% of mothers after randomisation. During the screening phase these events were primarily pregnancy and perinatal conditions (31/37), whereas infections were the majority after randomisation (21/33).

### Infant Safety

There were 13 infant deaths (3.2%; 95% CI 2%–5%); ten were HIV-infected, three in the NVP-only arm, five in the NVP/CBV4 arm, and two in the NVP/CBV7 arm. The causes of death in the HIV-infected infants were either gastroenteritis or respiratory infections; the oldest child to die in this group was 10 mo old. Three HIV-negative infants died, one from congenital pneumonia (NVP-only arm), one from complications of trisomy 18 (NVP/CBV4 arm), and one sudden infant death (NVP/CBV4 arm) without any preceding illness at 37 d after delivery. Suspected drug-related reactions were reported in 1.2% of infants (5/411). The events were hyperamylasaemia in the NVP-only arm (1/77), abdominal pain and vomiting in the NVP/CBV4 arm (2/164), and vomiting and jaundice in the NVP/CBV7 arm (2/170). The jaundice in the NVP/CBV7 arm was classified as serious. The most common abnormal laboratory tests were below-normal haemoglobin and above-normal bilirubin levels. There were no significant trends associated with CBV use.

## Discussion

This study shows that the addition of short course AZT and 3TC (for either 4 or 7 d) to sdNVP, initiated at the same time as the dose of NVP for PMTCT in antiretroviral-naïve pregnant women and their infants, results in a reduction in the proportion of mothers with new NNRTI-resistant mutations, when measured at 2 and 6 wk postdelivery. The nature of these mutations was also altered with fewer emergent mutations per woman being found. A reduction was also demonstrated in new NNRTI-resistant mutations in HIV-infected infants. All three regimens were safe in both mothers and infants and the majority of deaths (100% for mothers and 78% for infants) were HIV-related. Of HIV-infected infants who died, all were in utero infections.

The 4 and 7 d AZT plus 3TC with sdNVP combination regimens in this study were similarly efficacious in preventing emergence of resistance in the 6 wk after NVP exposure: 80.5% in the NVP/CBV4 arm and 87.8% in the NVP/CBV7 when compared to NVP-only. However, 7 d of NVP/CBV resulted in an earlier return to wildtype virus than the shorter course. The presentation in 2004 of preliminary results of this study, demonstrating a significant reduction in the selection of resistance compared to NVP alone, prompted the inclusion of a short course CBV regimen in the World Health Organisation guidelines for the management of pregnant HIV-infected mothers [17].

NNRTI resistance selected after sdNVP regimens [18] appears to compromise the response to NVP-based HAART, especially if started soon after the initial NVP exposure [11],[13]. Women whose baseline CD4 count was ≤200 cells/mm^3^ were particularly prone to selection of resistance mutations.

In this study both CBV regimens were adhered to and were easy to provide and could be considered as additions to sdNVP for PMTCT in countries where more complex or longer PMTCT prophylactic regimens are not feasible. The optimal length of postpartum cover of the prolonged NVP “tail” is not known, it is possible that additional benefit may be obtained from longer courses of antiretroviral agents, given the length of persistence of NVP following a single dose [4]. However, two subsequent studies have supported a short course “tail cover” approach [19],[20]. In the Zambian study, the addition of one dose of tenofovir and emtricitabine to the maternal intrapartum NVP dose reduced selection of NNRTI resistance by 53% [21]. Women in the Zambian trial with CD4+ cell counts less than 200/mm^3^ were excluded so they could be treated with HAART and all participants received antepartum AZT in addition to intrapartum NVP.

None of the mother-infant pairs in this trial had access to prophylaxis for in utero transmission of HIV-1, which was not available at the time in South Africa. The high in utero HIV-1 transmission rate we report supports the use of interventions earlier in pregnancy to prevent this. Combination antiretroviral therapy during pregnancy has decreased HIV-1 MTCT transmission in well-resourced settings to <2% [22]. The correlation between baseline CD4 and development of NNRTI-resistant mutations we report supports the initiation of HAART during pregnancy in women with low CD4 counts, in line with World Health Organisation and other national and international guidelines. SdNVP with 4 or 7 d of CBV, as used in this study, suppressed HIV viral loads to <400 copies/ml in the majority of mothers at 7–10 d after the final dose, but the viral load had returned to pretreatment levels by 6 wk postpartum.

The limitations of this study include that it was conducted in predominantly HIV clade C-infected participants in South Africa, although NNRTI resistance has been shown to be more common following sdNVP with this rather than other clades [23]. The exclusion of women with viral loads of less than 2,000 copies/ml may have resulted in a greater effect of CBV in preventing resistance mutations than if all women were randomised irrespective of their viral load. Furthermore, by chance, there were higher rates of baseline resistance in the CBV4 group than in the other groups. We used a relatively insensitive method of detecting resistance; use of more sensitive methods able to detect minority populations of resistant virus [9],[24], arguably may show less of an effect if higher rates of resistance were to be found in all arms. None of the women we included in this study had received AZT prior to receipt of study drugs; we are therefore unable to show the combined effect of several weeks of AZT given prenatally in conjunction with sdNVP and 4 or 7 d of AZT plus 3TC started at the onset of labour. Finally, the study was not powered to discriminate between rates of resistance we found in the two CBV-containing arms.

This trial, however, provides clear evidence that the selection of HIV-1 NNRTI resistance mutations by sdNVP can safely be reduced in mothers and HIV-infected infants by the addition of 4 or 7 d of AZT plus 3TC started at the time of the NVP dose.

## Supporting Information

Text S1 Protocol prior to amendments.
(0.49 MB DOC)

Text S2 Protocol amendment 1.
(0.20 MB DOC)

Text S3 Protocol amendment 2.
(0.24 MB DOC)

Text S4 Protocol amendment 3.
(0.25 MB DOC)

Text S5 Protocol amendment 4.
(0.07 MB DOC)

Text S6 Protocol amendment 5.
(0.06 MB DOC)

Text S7 Protocol amendment 6.
(0.14 MB DOC)

Text S8 Protocol amendment 7.
(0.25 MB DOC)

Text S9 CONSORT checklist.
(0.06 MB DOC)

## Abbreviations

AZT: zidovudine
3TC: lamivudine
CBV: combivir
CI: confidence interval
HAART: highly active antiretroviral therapy
IQR: interquartile range
MTCT: mother-to-child transmission
NNRTI: non-nucleoside reverse-transcriptase inhibitor
NVP: nevirapine
PMTCT: prevention of mother-to-child transmission
sdNVP: single-dose nevirapine
