# Supplementary material for: Efficacy of Short-Course AZT Plus 3TC to Reduce Nevirapine Resistance in the Prevention of Mother-to-Child HIV Transmission: A Randomized Clinical Trial
Source: PLoS Med. 2009 Oct 27;6(10):e1000172. doi: 10.1371/journal.pmed.1000172 (PMC2760761; doi:10.1371/journal.pmed.1000172)
Supplement: Text S1 — Protocol prior to amendments. (0.49 MB DOC) [file pmed.1000172.s001.doc]

| Boehringer Ingelheim (Pty) Ltd, South Africa | | | ABCD | |
| --- | --- | --- | --- | --- |
| Clinical Trial Protocol | | |  | Boehringer Ingelheim (Pty) Ltd, South Africa, 404 Main Avenue, Randburg, 2125, South Africa |
|  | BI Trial No.: | 1100.1413 | | |
|  | Investigational: product(s) | Nevirapine | | |
|  | Title: | An Open-label Study evaluating the Resistance profile of Single dose Nevirapine(NVP) when combined with a 4 or 7 day course of Combivir® (ZDV/3TC) compared to Single dose Nevirapine for the Prevention of Mother to Child Transmission (pMTCT) of HIV - Treatment Options Preservation Study (T.O.P.S.) | | |
|  | Clinical Phase: | III | | |
|  | Trial Clinical Monitor: | Dr J. Steytler  404 Main Avenue  Ferndale, Randburg  South Africa  2194 | | |
|  | Coordinating Investigator: | Prof. James McIntyre | | |
|  | Institute/ Department(s): | HIV Perinatal Research Unit  Chris-Hani Baragwanath Hospital  Soweto | | |
|  | Date of Protocol: | 19 June 02 | | |
|  | Planned Dates of Trial: | October 2002 – June 2004 | | |
|  |  | Page 1 of 66 | | |
|  | Confidential | Ó Boehringer Ingelheim This protocol is the property of Boehringer Ingelheim and may not - in full or in part - be  passed on, reproduced, published or otherwise use without the express permission of  Boehringer Ingelheim | | |

**LOCAL INFORMATION**

This page provides important local information, such as local Boehringer Ingelheim OPU, CML, (principal) investigator at the site as well as more information to be decided by the local Boehringer Ingelheim OPU, on basis of local laws and regulations, and Boehringer Ingelheim OPU local requirements.

| **Local Boehringer Ingelheim OPU:** | Boehringer Ingelheim (Pty) Ltd  404 Main Avenue  Ferndale, Randburg  2194 |
| --- | --- |
| **Clinical Monitor Local: (to be contacted in the event of an emergency)** | Dr J. Steytler  404 Main Avenue  Ferndale, Randburg, 2125  South Africa  Tel : +27-11-3482661  Cell : 082-4129548 |
| **Investigator:** |  |
| **Institute/ Department:** |  |
| **Local Extra: (function)** |  |
| **Institute/ Department:** |  |

# ABBREVIATIONS

| µg/mL | Micrograms per millilitre |
| --- | --- |
| 3TC | Lamivudine |
| ACTG | Aids Clinical Trials Group |
| AE | Adverse event |
| AIDS | Acquired Immune Deficiency Syndrome |
| ALP | Alkaline Phosphatase |
| ALT | Alanine Aminotransferase( Serum Glutamate-Pyruvate-Transaminase) |
| ARV | Antiretroviral |
| AST | Aspartate Aminotransferase( Serum Glutamate-Oxaloacetate-Transaminase) |
| BID | Twice daily |
| CD4 | Lymphocyte expressing CD4 surface marker |
| CML | Clinical monitor local |
| CRA | Clinical Research Associate |
| CRF | Case report file |
| DAIDS | Division of AIDS |
| DNA | Deoxyribonucleic acid |
| EC | Ethics Committee |
| ELISA | Enzyme linked immunosorbent assay |
| FDA | Federal Drug Administration |
| g/dl | Gram per decilitre |
| g/mL | Grams per millilitre |
| GCP | Good Clinical Practice |
| HAART | Highly active antiretroviral therapy |
| HIV | Human Immunodeficiency Virus |
| HIV-1 | Human Immunodeficiency Virus type one |
| IC_50_ | The concentration producing 50% inhibition |
| ICH | International Conference of Harmonisation |
| IEC | Institutional Ethics Committee |
| IRB | Institutional Review Board |
| LFT | Liver function test |
| LFTS | Liver function tests |
| mg/kg | Milligram per kilogram |
| mL | millilitre |
| mm3 | Cubic millimeter |
| MTCT | Mother to child transmission |
| nM | nanomolar |
| NNRTI | Non Nucleoside Reverse Transcriptase Inhibitor |
| NRTI | Nucleoside Reverse Transcriptase Inhibitor |
| NVP | Nevirapine |
| OPU | Operating unit |
| PCR | Polymerase chain reaction |
| pMTCT | Prevention of mother to child transmission |
| RNA | Ribonucleic acid |
| RT | Reverse Transcriptase (enzyme) |
| SAE | Serious Adverse Event |
| SJS | Stevens Johnson Syndrome |
| SOP | Standard Operating Procedure |
| ULN | Upper limit of normal |
| ZDV | Zidovudine |

# CLINICAL TRIAL PROTOCOL SUMMARY

| Name of company: Boehringer Ingelheim | | | Tabulated Study Protocol | |  |
| --- | --- | --- | --- | --- | --- |
| Name of finished product: VIRAMUNE®(nevirapine) | | |  |  |  |
| Name of active ingredient: Nevirapine | | |  |  |  |
| Protocol date:  19 June 2002 | | Trial Number: 1100.1413 | Planned Study period: 12 months | |  |
| Title of study: An Open-label Study evaluating the Resistance profile of Single dose Nevirapine (NVP) when combined with a 4 or 7 day course of Combivir® (ZDV/3TC) compared to Single dose Nevirapine for the Prevention of Mother to Child Transmission(pMTCT) of HIV- Treatment Options Preservation Study (T.O.P.S.) | | | | | |
| Investigators: Multicentre | | | | | |
| Study centre(s) : South Africa | | | | | |
| Clinical phase : III | | | | | |
| Objectives: To determine whether a regimen of single dose nevirapine combined with either 4 or 7 days of Combivir®, compared to a regimen of single dose nevirapine, for the prevention of mother to child transmission can reduce the rate of development of drug resistant mutations of HIV-1, in HIV-1 infected pregnant women, who have not received antiretroviral therapy previously. | | | | | |
| Methodology: This is an open-label, randomised, multicentre study | | | | | |
| No. of subjects:  total:  each treatment: | 300 HIV infected pregnant women and their infants randomised to a 1:1:1 ratio | | | | |
| Diagnosis and main criteria for inclusion: HIV-infected pregnant women with a HIV RNA viral load measurement >2000 copies/mL | | | | | |
| Test product:  Dose:    mode of admin.: | 1) Nevirapine tablets and Paediatric suspension  2) Combivir® tablets  3) Zidovudine syrup and 3 TC solution  1) Nevirapine 200mg single dose to mothers, infants 2mg/kg single dose  2) Combivir® one tablet twice daily to mother  3) Zidovudine 12mg twice daily and 3TC 6 mg twice daily to infants.  Oral | | | | |
| Duration of treatment: | Mothers to receive a single dose of nevirapine or a single dose of nevirapine and either 4 or 7 days of Combivir®.  Infants to receive a single dose of nevirapine or a single dose of nevirapine and either 4 or 7 days of Zidovudine and 3TC | | | | |
| Reference therapy:  dose:  mode of admin.: | None | | | | |
| Criteria for efficacy | The percent of mothers with HIV-1 isolates with new NNRTI drug resistance mutations identified by genotypic resistance testing and the percent of infants with evidence of HIV-1 infection at 6 weeks post partum defined as a positive HIV DNA or RNA PCR confirmed on >= 2 visits. The percentage of infants with genotypic NNRTI resistance mutations. | | | | |
| Criteria for safety: | Adverse events, vital signs, physical examination and laboratory evaluations | | | | |
| Statistical methods: Statistical models for primary analysis will compare treatment groups without adjustment for covariates. | | | | | |

# FLOW CHART

MOTHER

| Visit Number | 1 | 2 | 3 | 4 | **5^2, 3^** | **6**** | **7**** | **8^5, 8^**** |  |
| --- | --- | --- | --- | --- | --- | --- | --- | --- | --- |
|  | Screening  (Prenatal) | Enrollment  (Labour and delivery) |  |  |  |  |  | Final Visit | End of trial |
| Day | -14 to -1 | 0 | 1-2 | 14 (± 2) | 42 (± 6) | 90  ± 10) | 168  (± 10) | 336  (± 14) |  |
| Informed Consent | X |  |  |  |  |  |  |  |  |
| HIV ELISA Test | X^9^ |  |  |  |  |  |  |  |  |
| Demographics | X |  |  |  |  |  |  |  |  |
| Review Inclusion/Exclusion criteria | X | X |  |  |  |  |  |  |  |
| Medical History | X |  |  |  |  |  |  |  |  |
| Randomisation |  | X |  |  |  |  |  |  |  |
| Post delivery history |  |  | X |  |  |  |  |  |  |
| HIV related symptoms and signs | X |  | X |  | X | X | X | X |  |
| Physical Examination | X |  | X |  | X | X | X | X |  |
| Viral load PCR (RNA) | X | X | X | X | X | X | X | X |  |
| CD4 | X | X | X | X | X | X | X | X |  |
| Laboratory Tests ^4^ | X |  | X | X | X |  |  |  |  |
| Labour and Delivery History |  | X |  |  |  |  |  |  |  |
| Adverse Events | X | X | X | X | X | X | X | X |  |
| Concomitant Therapy | X | X | X | X | X | X | X | X |  |
| Drug Administration |  | X^1^ | X^1^ |  |  |  |  |  |  |
| Drug accountability/compliance |  | X | X | X |  |  |  |  |  |
| Sampling for Resistance testing | X | X | X | X | X | X | X | X |  |
| End of trial admin/trial completion |  |  |  |  |  |  |  |  | X^2,3,5^ |

INFANT

| **Visit Number** | **2** | **3** | **4** | **5^2 ,3^** | **5.1*** | **6**** | **7**** | **8^58^**** |  |
| --- | --- | --- | --- | --- | --- | --- | --- | --- | --- |

| **Day** | **0** | **1−2**  **(within 0-72 hrs)** | **14**  **(± 2)** | **42**  **(± 6)** | **49**  **(± 6)** | **90**  **(± 10)** | **168**  **(± 10)** | **336**  **(± 14)** | **End of trial** |
| --- | --- | --- | --- | --- | --- | --- | --- | --- | --- |

| Neonatal History (Including Apgar score). |  | X |  |  |  |  |  |  |  |
| --- | --- | --- | --- | --- | --- | --- | --- | --- | --- |
| Patient Demographics |  | X |  |  |  |  |  |  |  |
| Eligibility criteria |  | X |  |  |  |  |  |  |  |
| Record infant feeding method |  | X | X | X |  | X | X | X |  |
| Physical Examination |  | X | X | X |  | X | X | X |  |
| HIV related symptoms and signs |  |  |  | X |  | X |  | X |  |
| Laboratory Tests^4^ |  | X | X | X |  |  |  |  |  |
| PCR (RNA) |  | X | X | X | X | X | X | X |  |
| PCR (DNA) |  | X^7^ | X | X² | X |  |  |  |  |
| Drug Administration |  | X^1^, ^6^ |  |  |  |  |  |  |  |
| Drug Accountability/compliance |  | X | X |  |  |  |  |  |  |
| Adverse Events |  | X | X | X |  | X | X | X |  |
| Concomitant Therapy |  | X | X | X |  | X | X | X |  |
| Sampling for Resistance testing |  | X | X | X | X | X | X | X |  |
| End of trial admin/trial completion |  |  |  |  |  |  |  |  | X^2,3,5^ |

1. All mothers to receive a single dose of nevirapine in labour and will be randomised to either no Combivir® or 4 or 7 days of Combivir® , also to be administered while in labour. Infants to receive the same treatment as mother.
2. Visit 5 concludes patient participation if mother and infant do not demonstrate resistance or the infant remains HIV DNA PCR negative.
3. Visit 5 also to be completed ,if possible, for all dropouts and withdrawals prior to visit 5.
4. Laboratory tests refers to : Full blood count, serum creatinine, AST, ALT, ALP, Total bilirubin, amylase.
5. Visit 8 concludes mother and infant participation in the trial for those mothers and infants with demonstrated resistance.
6. Retrovir® and 3TC® administered to infant within 24 hours after birth , nevirapine , to be administered 24-72 hours after birth.
7. Initial HIV DNA PCR to be performed within 48 hours.
8. Visit 8 also to be completed, if possible, for all dropouts and withdrawals after visit 5.
9. Unless a positive HIV-1 ELISA test is documented.

* Extra visit for infants who test HIV DNA PCR positive for first time at visit 5.

****** **Only for those patients with resistant virus**

# TABLE OF CONTENTS

ABBREVIATIONS [3](#__RefHeading___Toc503678816)

CLINICAL TRIAL PROTOCOL SUMMARY [3](#__RefHeading___Toc503678817)

FLOW CHART [3](#__RefHeading___Toc503678818)

TABLE OF CONTENTS [3](#__RefHeading___Toc503678819)

1. introduction [3](#__RefHeading___Toc503678820)

1.1 Medical Background [3](#__RefHeading___Toc503678821)

1.2 Drug Profile [3](#__RefHeading___Toc503678822)

1.3 Description and Rational for Performing the Trial [3](#__RefHeading___Toc503678823)

1.4 Benefit / Risk Assessment [3](#__RefHeading___Toc503678824)

2. study objectives [3](#__RefHeading___Toc503678825)

2.1 GENERAL AIM / PRIMARY OBJECTIVE [3](#__RefHeading___Toc503678826)

2.2 PRIMARY ENDPOINT(S) [3](#__RefHeading___Toc503678827)

2.3 SECONDARY ENDPOINTS [3](#__RefHeading___Toc503678828)

3. Study population [3](#__RefHeading___Toc503678829)

3.1 NUMBER OF SUBJECTS PLANNED [3](#__RefHeading___Toc503678830)

3.2 INCLUSION CRITERIA [3](#__RefHeading___Toc503678831)

3.3 EXCLUSION CRITERIA [3](#__RefHeading___Toc503678832)

4. treatments [3](#__RefHeading___Toc503678833)

4.1 TREATMENTS TO BE COMPARED 20

4.1.1 Investigational product 20

4.1.2 Comparator drugs(s) or intervention(s) 20

4.1.3 Dosage and treatment schedule 21

4.1.4 Packaging, labeling and resupply 21

4.1.5 Storage conditions [3](#__RefHeading___Toc503678839)

4.2 CONCOMITANT THERAPY [3](#__RefHeading___Toc503678840)

4.2.1 Rescue medication and additional treatment(s) [3](#__RefHeading___Toc503678841)

4.2.2 Restrictions [3](#__RefHeading___Toc503678842)

4.3 TREATMENT COMPLIANCE [3](#__RefHeading___Toc503678843)

5. observations [3](#__RefHeading___Toc503678844)

5.1 EFFICACY [3](#__RefHeading___Toc503678845)

5.1.1 Primary endpoint(s) [3](#__RefHeading___Toc503678846)

5.1.2 Secondary endpoints [3](#__RefHeading___Toc503678847)

5.2 SAFETY [3](#__RefHeading___Toc503678848)

5.3 OTHERS 19

6. investigational plan [3](#__RefHeading___Toc503678850)

6.1 STUDY DESIGN AND PLAN [3](#__RefHeading___Toc503678851)

6.2 STUDY PROCEDURES AT EACH VISIT [3](#__RefHeading___Toc503678852)

6.3 ADHERENCE TO PROTOCOL [3](#__RefHeading___Toc503678853)

6.3.1 Visit schedule [3](#__RefHeading___Toc503678854)

6.3.2 Criteria and rules for stopping subject treatment [3](#__RefHeading___Toc503678855)

7. statistics [3](#__RefHeading___Toc503678856)

7.1 STATISTICAL DESIGN / MODEL [3](#__RefHeading___Toc503678857)

7.2 NULL AND ALTERNATIVE HYPOTHESES [3](#__RefHeading___Toc503678858)

7.3 PLANNED ANALYSES [3](#__RefHeading___Toc503678859)

7.3.1 Primary analyses [3](#__RefHeading___Toc503678860)

7.3.2 Secondary analyses [3](#__RefHeading___Toc503678861)

7.3.3 Safety analyses [3](#__RefHeading___Toc503678862)

7.3.4 Interim analyses [3](#__RefHeading___Toc503678863)

7.4 HANDLING OF MISSING DATA [3](#__RefHeading___Toc503678864)

7.5 RANDOMISATION [3](#__RefHeading___Toc503678865)

7.6 SAMPLE SIZE ISSUES [3](#__RefHeading___Toc503678866)

8. ADMINISTRATIVE MATTERS [3](#__RefHeading___Toc503678867)

8.1 ETHICS [3](#__RefHeading___Toc503678868)

8.1.1 Institutional Review Board or Independent Ethics Committee [3](#__RefHeading___Toc503678869)

8.1.2 Informed Consent and Subject Information [3](#__RefHeading___Toc503678870)

8.2 RECORDS [3](#__RefHeading___Toc503678871)

8.2.1 Drug Accountability [3](#__RefHeading___Toc503678872)

8.2.2 Emergency code break [3](#__RefHeading___Toc503678873)

8.2.3 Case Report Forms [3](#__RefHeading___Toc503678874)

8.2.4 Source documents [3](#__RefHeading___Toc503678875)

8.2.5 Direct access to source data / documents [3](#__RefHeading___Toc503678876)

8.3 QUALITY ASSURANCE AUDIT [3](#__RefHeading___Toc503678877)

8.4 PROCEDURES [3](#__RefHeading___Toc503678878)

8.4.1 Adverse Events [3](#__RefHeading___Toc503678879)

8.4.2 Emergency procedures [3](#__RefHeading___Toc503678880)

8.5 RULES FOR AMENDING PROTOCOL [3](#__RefHeading___Toc503678881)

8.6 DISCONTINUATION OF THE TRIAL BY THE SPONSOR [3](#__RefHeading___Toc503678882)

8.7 STATEMENT OF CONFIDENTIALITY [3](#__RefHeading___Toc503678883)

8.8 PUBLICATION POLICY [3](#__RefHeading___Toc503678884)

9. SIGNATURE PAGE(S) [3](#__RefHeading___Toc503678885)

10. references [3](#__RefHeading___Toc503678886)

11. Appendices [3](#__RefHeading___Toc503678887)

11.1 Appendix I - Viramune® (Nevirapine) Clinical

Trials Hepatotoxicity Management Schema 51

11.2 Rash Management Guidelines 53

11.3 ACTG TABLE FOR GRADING ADULT ADVERSE EXPERIENCES 54

11.4 Division of Aids (DAIDS) Toxicity Table for

Grading Severity of Paediatric (≤ 3 months and >3months of age) adverse experiences 62

# 1. introduction

## Medical Background

### The rate of mother to child transmission of HIV can be as high as 40 % in developing countries. The risk of such transmission is highest during labour and delivery. The likelihood of transmission of virus is dependent on a number of factors including, but not limited to, the concentration of virus in genital tract secretions, the length of exposure of the new-born to such secretions and the viral load of the mother.

### High levels of circulating HIV in maternal blood at time of delivery increases the risk of transmission to the infant. Antiretroviral treatment can result in significant short term reductions in viral load when given to mothers in labour. Treatment of the infant can further offer protection against transmission of the virus. (R01- 1178, P99-02572).

### The presence of an antiretroviral drug in a patient can change the viral ecology dramatically. If the therapy allows continued viral replication,(as all single drug and most dual therapies do),HIV variants with drug resistant mutations may become selected as the dominant species as long as the drug is administered. The rate at which mutant virus replaces the original dominant variant (wildtype) is a function of the overall replication rate of HIV in the person and the degree of growth advantage the mutation confers.(P99-12030).

Evaluation of HIV-1 resistance profiles in prevention of MTCT trials have demonstrated the emergence of isolates with nevirapine –specific genotypic mutations in treatment naïve mothers who have received a single dose(or two dose) 200mg regimen.(P01-06388 , U01-3282).The clinical significance of this resistance is yet to be determined and since nevirapine has a long half life , this long exposure to functional “monotherapy” concentrations of nevirapine may increase the risk of resistance. This study is to evaluate whether the addition of Combivir® ,thus avoiding functional monotherapy, could decrease the emergence of nevirapine resistance.

## Drug Profile

### Nevirapine(NVP), a dipyridodiazepinone, is a potent non nucleoside HIV –1 Reverse Transcriptase inhibitor with an IC_50_ of 40 nM (0.01 µg/mL), and a high therapeutic index.(P 90-4449)It is an agent with high bioavailability,( 93% absolute bioavailability for the tablet formulation) that is active in the native state and has a long half-life resulting in a simple dose regimen with no food effects. (R 00-1002) Pharmacokinetic studies in HIV-1 infected women confirm that nevirapine readily crosses the placental barrier and is found in breastmilk.(P98-7078) .

### The most frequently reported adverse events related to nevirapine are rash, fever, nausea, headache and abnormal liver function tests (LFTS) in patients receiving chronic dosing.

### The major clinical toxicity of nevirapine is rash .Nevirapine attributable rash occurred in 16% of patients in combination regimes in phase II/III controlled studies . Nevirapine – associated rashes are usually mild to moderate with the majority of severe rashes occurring after multiple doses within the first 28 days of treatment. Severe or life threatening rashes (grade 3 or 4) occurred in 6.6% of nevirapine treated patients compared with 1.3% in control groups.(R01- 1197).

### Of 2861 patients exposed to multiple dose regimens of nevirapine there have been 9 confirmed cases of Stevens-Johnson Syndrome(SJS) , giving an incidence of 0.3%.(U91-0312).

### Abnormal liver function tests, including hepatitis, have been reported with nevirapine, some in the first few weeks of therapy. The single dose of nevirapine given to mothers in this study is unlikely to aggravate pre- existing liver pathology, however patients with LFT abnormalities will be carefully monitored. (APPENDIX 1).

### HIV-1 isolates exhibiting reduced susceptibility to nevirapine appeared within 4 weeks during tissue culture selection in the presence of nevirapine. The only mutation selected for in vitro was a change at amino acid 181 which conferred a 100 fold reduction in nevirapine susceptibility.(P93-7261).

### A number of additional mutations have been identified in HIV-1 clinical isolates obtained from nevirapine experienced patients. These mutations occur either singly or in combination and map to the nevirapine binding pocket at RT amino acids 103,106,108,181,188 and 190). (P94-5965,P94-6218).

### The effect of multiple mutations is usually an additive reduction in susceptibility to nevirapine. Resistance is apparently caused by either a reduction in binding affinity of the enzyme for the drug or by inducing steric hindrance, which prevents nevirapine from docking into the binding site.(P94-6421).

### Genotyping of clinical isolates has clearly shown that circulating virus is a mixture of HIV quasi species influenced by both past and current drug therapy. During nevirapine monotherapy mutation at position 181 is predominant, while changes at other residues 103, 108, 188 and 190 occur less frequently.(P94-5965, P94-6218).Mutations at residues 103, 106, 188 and 190 occurs most frequently during nevirapine and zidovudine (ZDV)dual therapy.

### Nevirapine experience in HIV infected pregnant women (HIVNET 006/012) has demonstrated that nevirapine prophylaxis during labour is a simple inexpensive regimen which can dramatically reduce the risk of HIV-1 vertical transmission especially in the setting of developing countries.(P99-00967, P99-02572).

### However it has also been suggested (HIVNET 012,) that resistance to NNRTI drugs may be induced in the setting of nevirapine prophylaxis in mother to child transmission after a single dose even at a low level of viral replication(viral load < 10000 copies/mL).This study demonstrated that the rate of development of nevirapine resistance is approximately 18% after a single dose at 6 weeks after exposure, but by approximately 18 months post delivery, there were no resistance mutations detected.(P01-06388).

### The SAINT study (1100.1287) in which 2 doses of nevirapine were administered to pregnant mothers has in fact suggested a higher rate of nevirapine resistance especially for the K103N mutation. (U01-3282).

### Six mutations associated with zidovudine resistance have been identified in HIV-1 RT , at codons 41, 67, 70, 210, 215 and 219.The sequential appearance of these mutations is associated with increasing levels of resistance. Strains with the first mutation to appear (usually at position 70 ) are at most 8-fold less susceptible to zidovudine , whereas isolates which have acquired 4 mutations(at positions 41,67,70 and 215) are over 100- fold less susceptible¹.

### Lamuvidine has a unique resistance profile. Two mutations that were described early in its development that confer resistance both involve a single amino acid change at codon 184 of RT. Mutation M184I appears early in the course of treatment , but is rapidly replaced by M184V , which confers high level resistance with mono- or dual therapy. However with HAART, selection of M184V is significantly delayed. Furthermore M184V has been shown to suppress the effects of the M41I and T215Y zidovudine resistance mutations , effectively sensitising virus to zidovudine¹.

### Two further mutations , E44D and V118I , which can emerge in NRTI – experienced subjects confer modest lamuvidine resistance¹.

### Because nevirapine (as with other NNRTI‘s) is a drug with a low genetic barrier(a single mutation in the RT genome induces high level phenotypic resistance) it should be used for treatment as part of a potent antiretroviral combination to ensure suppression of viral replication thus circumventing the rapid selection of (cross)resistant variants(P00-14835).

### This raises the question: can resistance be avoided by combining nevirapine with CBV during the 4-7 day period following nevirapine single dose administration for the prevention of MTCT?

## Description and RationalE for Performing the Trial

### Numerous trials have already demonstrated the safety and efficacy of ZDV alone, ZDV + 3TC, and nevirapine (ACTG 076, Thai, PETRA , HIVNET 006/012, SAINT) in reducing the rate of transmission of HIV from mother to child.

Evaluations of HIV-1 resistance patterns in trials of pMTCT have demonstrated nevirapine resistant HIV-1 isolates in approximately 15-20% of mothers 4-6 weeks after receiving either a single or two dose 200mg nevirapine regimen. Although the ability to detect these genotypic mutations decreases to 0% by about 18 months, it is not clear whether this resistance is clinically significant.(HIVNET 012).

Empirically then it would seem useful to develop a strategy to diminish the emergence of this early resistance, therefore this study is proposed to evaluate whether the effect of 4 or 7 days of Combivir®(3TC+ ZDV) added to a single dose nevirapine regimen for the prevention of MTCT will prevent the emergence of resistance to nevirapine. In addition infants will receive the same treatment regimen as their mother.

The study will be conducted as an open label three arm study involving a population of antiretroviral naïve HIV infected pregnant women and their newborn infants. Only mothers with a viral load of > 2000 copies/mL will be included in the study.

##### **BENEFIT / RISK ASSESSMENT**

The safety and efficacy of nevirapine , ZDV and 3TC in studies on the prevention of mother to child transmission has already been established. It is postulated that this treatment options preservation study will demonstrate a reduced rate of development of drug resistance utilising the combined nevirapine/ZDV&3TC regimens.

# 2. study objectives

## GENERAL AIM / PRIMARY OBJECTIVE

### To determine whether a regimen of single dose nevirapine combined with either 4 or 7 days of Combivir®, compared to a regimen of single dose nevirapine, for the prevention of mother to child transmission can reduce the rate of development of drug resistant mutations of HIV-1, in HIV-1 infected pregnant women, who have not received antiretroviral therapy previously.

### PRIMARY ENDPOINT

The percentage of mothers with HIV-1 isolates with new NNRTI drug resistant mutations identified by genotypic sequencing of specimens collected within six weeks following delivery.

## SECONDARY ENDPOINTS

1. The percentage of infants with evidence of HIV-1 infection at 6weeks.

Infants who test positive for HIV DNA by PCR within 48 hours of birth will be defined as having been infected during the intrauterine period and will be excluded from the primary analysis. An infant will be considered HIV positive if the initial HIV PCR (within 48 hrs) is negative and 2 consecutive HIV PCR tests at least 1 week apart after 14 days of life are positive. An infant will be considered not intrauterine or peripartum infected if the HIV PCR remains negative at 6 weeks post partum.

1. The percentage of infected infants with NNRTI resistance-associated mutations will be stratified according to intrauterine or peripartum infected status.
2. The safety endpoint will be the safety measurements for mothers and infants through 6 weeks on each of the regimens.
3. The frequency of late resistance mutations in mothers and infants who demonstrated resistance mutations at 6 weeks.

# 3. Study population

## NUMBER OF SUBJECTS PLANNED

The total trial population will consist of 300 HIV-1-infected pregnant women, who meet the inclusion and exclusion criteria, and their neonates to achieve 240 mother-infant pairs who are evaluable. Patient participation will be for 6weeks unless resistance is demonstrated in which case patients will be followed up for 12 months. 5 study centres in South Africa are envisaged to provide a total of 300 patients.

## INCLUSION CRITERIA

### Pregnant women who present at antenatal clinics after 36 weeks gestation and are antiretroviral drug naïve.

### Mothers with a documented positive HIV ELISA test.

- Mother to have a baseline viral load of > 2000 RNA copies/mL.
- Written informed consent from the mother and father (if known and available) prior to the performance of any trial related procedures.
  1. **EXCLUSION CRITERIA**
- Mothers who, in the opinion of the investigator ,cannot be relied on to return with their infants for postnatal visits.
- Mothers who have received any antiretroviral drugs previously.
- Mothers who are not able to take oral medication.
- Mothers who present with an obstetric emergency, the sequelae of which in the investigators opinion will not permit oral dosing of study medication.
- Unwillingness or inability to reasonably comply with the protocol requirements.
- Use of any other investigational product during the pregnancy and for the duration of the study period.
- Patients with a recent history of pancreatitis or peripheral neuropathy.
- Patients with renal failure requiring dialysis.
- Patients with evidence of hepatic dysfunction as measured by total bilirubin > 2.5 times ULN or AST/ALT > 5 times ULN.
- Patients with any one of the following additional laboratory abnormalities at screening :

Haemoglobin concentration < 9.0 g/dl.

Neutrophil count < 750 cells/mm3.

Platelet count < 75,000 cells/mm3.

Serum amylase > 2 x ULN.

- A recent history ( during the pregnancy) of drug abuse or alcoholism.
- Mothers who will undergo elective caesarean section.
- If known prior to delivery, mothers with foetuses with anomalies incompatible with life.
- Mothers in whom amniocentesis is indicated.

**Note** : Infants born with malformations incompatible with life will not receive treatment but the mother –infant pair will remain in the trial. Infants with life threatening perinatal conditions which do not allow oral therapy will not receive treatment but remain in the trial. Mothers of these infants will continue to receive treatment and will remain in the study.

**4. TREATMENTS**

**4.1 TREATMENTS TO BE COMPARED**

**4.1.1** **Investigational product**

Nevirapine (Viramune ®) 200mg tablets and oral suspension 50mg/5ml.

**4.1.2 Comparator drugs or interventions**

Combivir® tablets (Zidovudine 300mg and 3TC 150mg).

Retrovir® oral syrup 50mg/5ml and 3TC® oral solution 10mg/ml.

### Dosage and treatment schedule

Mothers will be randomised to receive one of three regimens with initial dosing only permitted in the hospital ward :

1. A single dose of a nevirapine 200mg tablet during labour.
2. A single dose of a nevirapine 200mg tablet **plus** Combivir® (ZDV 300mg + 3TC 150mg) one tablet during labour, and repeated 12 hourly where necessary, followed by Combivir® tablets given as one tablet BID, for 4 days, commencing after delivery.
3. A single dose of a nevirapine 200mg tablet **plus** Combivir®(ZDV 300mg + 3TC 150mg) one tablet during labour , and repeated 12 hourly where necessary, followed by Combivir® tablets given as one tablet BID for 7 days.

Should a mother have been randomised to a CBV containing treatment arm and experience prolonged labour (>= 12 hours) , a further Combivir® tablet is dispensed at the beginning of each subsequent 12 hour period. Should a mother have been randomised to any treatment arm in false labour , and later discharged, she is to receive the same previously randomised treatment ,when presenting in active labour , with the clock reset to time zero.

Mothers randomised to the nevirapine only arm in false labour > 72 hours before presenting in active labour, will receive a second dose of nevirapine when presenting in active labour, but will be excluded from the primary analyses. Mothers with prolonged labour will not receive a second dose of nevirapine.

Mothers who do not retain administered medication ,within 1 hour of dose administration, will be given a repeat dose. Mothers who develop an obstetric emergency, after enrollment, which will not permit oral dosing of study medication , will continue to be followed up from a safety point of view until resolution of the emergency, but will not continue with the study. Infants who are able to take oral medication, born to these mothers will continue to participate in the study.

In situations of multiple births, all neonates born to the same mother, will receive the same randomised treatment as the mother.

Neonates will receive the same treatment as their mother, either:

1. A single dose of 2mg/kg nevirapine suspension within 24- 72 hours after birth.
2. A single dose of nevirapine (2mg/kg) administered within 24-72 hours after birth plus Retrovir® oral suspension 12 mg bid and 3 TC® oral suspension 6mg bid for 4 days commencing within 24 hrs after birth
3. A single dose of nevirapine (2mg/kg) administered within 24-72 hours after birth plus Retrovir® oral suspension 12 mg bid and 3 TC® oral suspension 6mg bid for 7 days commencing within 24 hrs after birth.

The neonatal dose will be rounded off to the nearest 0.1ml and administered using a 1ml syringe. Should the initial dose of medication not be retained within one hour of administration, a second dose will be administered. The neonate will be administered a 2mg/kg dose of nevirapine immediately post delivery if the mother was dosed within 2 hours of delivery or did not receive any medication prior to delivery and given a second dose 24-72 hours after birth. The neonate will receive 3TC® and Retrovir® immediately after delivery should the mother not receive Combivir® or receive a dose < 2 hours prior to delivery.

**Note:** For infants weighing < 2kg the dose of Retrovir® will be 4mg/kg bid and that of 3TC 2mg/kg bid.

### Packaging, labeling and resupply

#### Nevirapine 200mg tablets will be packaged in bottles containing 60 tablets. Combivir® obtained from commercial supply as bottles containing 60 tablets , with each tablet containing 300mg Retrovir® and 150mg 3TC , will be repackaged to provide either a 4 or 7 day supply of Combivir® to the patients. The Viramune® suspension will be supplied as 240ml bottles containing 50mg /5ml nevirapine. Retrovir® oral syrup will be supplied as 200ml bottles containing 50mg/5ml zidovudine while 3TC® oral solution will be supplied in bottles of 240 ml containing 10mg/ml lamivudine. Individual bottles will be labelled with drug name, lot no., expiry date, strength, dosing and storage instructions, protocol number, treatment number and caution statements in accordance with regulatory requirements.

### Storage conditions

#### All study medication should be stored in a secured area away from direct sunlight at the investigators site, at room temperature between 15°C and 25°C with a daily temperature log being maintained at the site. Access to the drug supply should be limited to individuals designated by the clinical investigator and approved by the sponsor for the purposes of preparing the study drug(s) and completing the drug accountability.

## 4.2 CONCOMITANT THERAPY

### 4.2.1 Concomitant medication and additional treatment(s)

All concomitant medication administered to a patient during study participation must be recorded on the appropriate pages in the patient’s CRF. All concomitant medication used in the last month prior to the screening visit and which is continuing at that time will be recorded in the CRF.

### 4.2.2 Restrictions

Other antiretroviral medication and all investigational drugs are excluded during the study and the current pregnancy.

- 1. **tREATMENT COMPLIANCE**

**Mothers:**

To assess compliance in mothers on the Combivir® arms, the number of Combivir® tablets and the dose and number of doses of nevirapine tablets used during the hospital stay will be recorded in the CRF.

The compliance percentage (of Combivir® dispensed after hospital discharge) will be determined as follows:

The number of tablets of Combivir® taken as indicated by a pill count X 100

The number of tablets which should have been taken as determined from the dosing schedule.

**INFANTS:**

In all cases the dose and number of doses of nevirapine received will be recorded in the CRF.

To assess compliance in those subjects on the zidovudine/lamuvidine arm the full bottle of medicine will be weighed prior to dispensing and the mass recorded in the CRF. The bottle and its contents will again be weighed when the patient returns at visit 4 and the mass recorded in the CRF.

The **volume of medicine** **administered** will be determined and recorded in the CRF using this formula:

Difference in recorded mass of bottle and contents (g)

Density of the syrup/solution (g/mL)

Compliance will be recorded as a percentage using the formula:

Volume of medicine administered to infant determined above x 100

Volume of medicine the infant should have consumed according to the dosing schedule.

**Note :**

The density of all study medication to be used will be obtained from the manufacturer and be provided to each site when supplies are delivered. The density of the batch used should be recorded in the CRF to facilitate automated calculations in the database.

# 5. observations

## 5.1 EFFICACY

**5.1.1 Primary endpoint(s)**

#### The primary efficacy variable for this study will be:

#### The percentage of mothers with HIV-1 isolates with new NNRTI drug resistance associated mutations identified by genotype testing at 6 weeks following delivery. New NNRTI drug resistance-associated mutations will be defined as the presence of mutations recognised as nevirapine resistant mutations and not present at baseline(screening).

### 5.1.2 Secondary endpoints

The secondary endpoints will be :

1. The percentage of infants with evidence of HIV-1 infection at 6weeks.

Infants who test positive for HIV DNA by PCR within 48 hours of birth will be defined as having been infected during the intrauterine period and will be excluded from the primary analysis. An infant will be considered HIV positive if the initial HIV PCR (within 48 hrs) is negative and 2 consecutive HIV PCR tests at least 1 week apart after 14 days of life are positive. An infant will be considered not intrauterine or peripartum infected if the HIV PCR remains negative at 6 weeks post partum.

1. The percentage of infected infants with NNRTI resistance-associated mutations will be stratified according to intrauterine or peripartum infected status.
2. The safety endpoint will be the safety measurements for mothers and infants through 6 weeks on each of the regimens.
3. The frequency of late resistance mutations in mothers and infants who demonstrated resistance mutations at 6 weeks.

## 5.2 SAFETY

Safety will be assessed through regular laboratory tests, physical examination and adverse event reporting. As the commonest adverse events with nevirapine are rash and liver toxicity, guidelines for liver toxicity and rash management are included as Appendix 1 and Appendix 2 respectively. All adverse events must be recorded according to the procedures noted in Section 8.4.1.

Adverse events

At each visit, all adverse events, regardless of causality, will be recorded on the adverse event CRF page after discussion with the patient. Adverse events will be recorded on CRFs as non-serious or serious adverse events (SAEs). Serious adverse events, whether or not considered related to the investigational drug, will be recorded on the Serious Adverse Event form located in the ISF and faxed to the Clinical Monitor Local as soon as site personnel are aware of the event. Every attempt should be made to collect discharge summaries for each hospitalisation to provide further details. Any adverse events, which are continuing at the time the patient discontinues from the study, must be followed until deemed sufficient by the investigator and the Boehringer Ingelheim clinical monitor or until the event has resolved In addition should the investigator become aware of any serious adverse drug related event after the observational period , this should be reported to the Sponsor. Additionally, all serious adverse events, regardless of their relationship to drug, that occur within 30 days after the patient terminates study medication must be reported according to Boehringer Ingelheim SAE procedure.

The definition of an adverse event for the purposes of this trial shall be any untoward change from the baseline condition of the mother, or infant, including intercurrent illness which occurs following administration of study drug whether it is considered drug related or not.

Division of AIDS (DAIDS) toxicity gradings (Appendix 3) will be used as a guideline to assess and manage laboratory and clinical adverse events. Adverse events of DAIDS grades 3 (severe) and 4 (potentially life threatening) should be reported as BI intensity “3”(severe). In general the following guidelines apply:

- Subjects who develop grade 1 or 2 AE / toxicity may continue study medications without dose adjustment.
- Grade 3 or 4 adverse events are more complex and will usually lead to discontinuation of study drug for the purposes of this study unless, the investigator has compelling evidence that the AE is not caused by study drug , the AE is not considered clinically relevant , or the benefits of therapy outweigh the importance of the AE. In these cases the dose of study drug will remain unaltered.

For the purposes of this study any event ,which leads to a caesarean section being performed, that is in the investigators opinion **drug related ,** or results in a **fatality,** will be reported as a **serious adverse event.** All other caesarean sections performed due to an obstetric complication will be regarded only as a **severe** adverse event.

Hepatotoxicity and rash are defined adverse events associated with nevirapine. Management guidelines have been included. (Appendices 1,2 and 3).

Clinical Laboratory Testing

Clinical laboratory testing for mothers which will include serum creatinine ,ALT, AST, ALP, Total Bilirubin, amylase, full blood count , CD4+ count, HIV RNA PCR (viral load), and HIV-1 resistance testing will be conducted on all patients at the screening visit (Visit 1) and repeated at all visits up to visit 5, excluding visit 2 for mothers (labour and enrollment), but including a withdrawal visit if this occurs during the initial 6 week follow up period. For all subsequent visits and for dropouts and withdrawals after visit 5 , blood should be collected wherever possible for CD4,viral load (HIV RNA PCR) and resistance and other HIV-1 virologic testing only.

Clinical laboratory testing for infants which will include, serum creatinine ,ALT, AST, ALP, Total Bilirubin, amylase, full blood count , HIV DNA PCR and HIV RNA PCR . HIV-1 resistance and other HIV-1 virologic testing should be performed **only if** the infant is HIV-infected. All these laboratory tests will be conducted at all visits up to visit 5 including wherever possible any withdrawal visit in that time period. At all subsequent visits, and any withdrawal visit after visit 5, laboratory testing will only include HIV RNA PCR , and HIV-1 resistance and other HIV-1 virologic testing. At visit 5.1 laboratory testing will include HIV DNA PCR , and only if the infant is HIV-1 infected will blood be submitted for HIV RNA PCR , and HIV-1 resistance and other HIV-1 virologic testing.

An explanation of the etiology of abnormal laboratory findings must be made on the case report form for any abnormalities for which an etiology is known. No dose adjustments to study medication will be allowed during the study based on abnormal laboratory findings, however, significant abnormal laboratory findings may result in a patient being excluded from participating in the study as indicated in section 3.3 (exclusion criteria) or may result in early withdrawal from the study as described in section 6.3.3. If any laboratory evaluation is repeated, for clarification of an abnormal finding, these results must also be recorded in the CRF. All laboratory tests will be performed at a local laboratory , the requirements for the handling of blood specimens will be contained in the laboratory manual provided to all the investigators participating in the study.

Physical Examination

A targeted physical examination will be completed on all mothers at the screening visit (Visit 1) and repeated at visit 3 (1-2 days post delivery) and visits 5-8 . The targeted physical examination will include measurements of pulse rate, temperature and weight, and examinations of the following systems : Cardiovascular , Respiratory, Central Nervous System, Abdomen , ENT and the integument. In addition mothers will have their blood pressure recorded. Infants will have a complete physical examination from head to toe , thereafter a targeted physical examination at all visits. All clinically significant findings at baseline will be recorded on the Medical History/Concomitant Diagnoses page in the patient’s case report form (CRF). New clinically significant findings detected at follow-up physical examinations will be recorded as adverse events on the appropriate CRF page.

# 6. investigational plan

## 6.1 STUDY DESIGN AND PLAN

A prospective randomised open label treatment options preservation study to determine whether a regimen of a single dose of 200mg of nevirapine combined with either 4 or 7 days of Combivir® can reduce the development of drug resistant HIV-1 in mothers who are antiretroviral drug naïve. Mothers will be enrolled during labour if all inclusion criteria have been met and will be randomised to one of the three treatment arms. Women in labour after 36 weeks gestation will receive either:

1. A single dose of a nevirapine 200mg tablet during labour.
2. A single dose of a nevirapine 200mg tablet **plus** Combivir® (ZDV 300mg + 3TC 150mg) one tablet during labour , and repeated 12 hourly where necessary, followed by Combivir® tablets, given as one tablet BID , for 4 days, commencing after delivery.
3. A single dose of a nevirapine 200mg tablet **plus** Combivir® (ZDV 300mg + 3TC 150mg) one tablet during labour , and repeated 12 hourly where necessary ,followed by Combivir® tablets, given as one tablet BID , for 7 days, commencing after delivery.

Should a mother have been randomised to a CBV containing treatment arm and experience prolonged labour (>= 12 hours) , a further Combivir® tablet is dispensed at the beginning of each subsequent 12 hour period. Should a mother have been randomised to any treatment arm in false labour , and later discharged, she is to receive the same previously randomised treatment ,when presenting in active labour , with the clock reset to time zero.

Mothers randomised to the nevirapine only arm in false labour > 72 hours before presenting in active labour, will receive a second dose of nevirapine when presenting in active labour, but will be excluded from the primary analyses. Mothers with prolonged labour will not receive a second dose of nevirapine.

Mothers who do not retain administered medication, within 1 hour of dose administration, will be given a repeat dose. Mothers who develop an obstetric emergency, after enrollment which will not permit oral dosing of study medication, will continue to be followed up from a safety point of view until resolution of the emergency, but will not continue with the study. Infants who are able to take oral medication, born to these mothers, will continue to participate in the study.

In situations of multiple births, all neonates born to the same mother, will receive the same randomised treatment as the mother.

Neonates will receive the same treatment as their mother, either :

1. A single dose of 2mg/kg nevirapine suspension within 24- 72 hours after birth.
2. A single dose of nevirapine(2mg/kg) administered within 24-72 hours after birth plus zidovudine oral suspension 12 mg bid and 3 TC® oral suspension 6mg bid for 4 days commencing within 24 hrs after birth.
3. A single dose of nevirapine(2mg/kg) administered within 24-72 hours after birth plus zidovudine oral suspension 12 mg bid and 3 TC® oral suspension 6mg bid for 7 days commencing within 24 hrs after birth.

Infants with a weight of < 2kg will have their doses of ZDV and 3TC adjusted to 4mg/kg bid and 2mg/kg bid respectively.

The neonatal dose will be rounded off to the nearest 0.1ml and administered using a 1ml syringe. Should the initial dose of medication not be retained within one hour of administration, a second dose will be administered. The neonate will be administered a 2mg/kg dose of nevirapine immediately post delivery if the mother was dosed within 2 hours of delivery or did not receive any medication prior to delivery and given a second dose 24-72 hours after birth. The neonate will also receive Retrovir® and 3TC immediately after delivery, should the mother not receive a dose of Combivir® or receive a dose < 2 hours prior to delivery.

- 1. **STUDY PROCEDURES AT EACH VISIT**

**Mothers : (refer to flow chart)**

**Visit 1: Day –14 to -1 (prenatal screening)**

- Review inclusion and exclusion criteria.
- Perform an HIV Elisa test, with written informed consent and appropriate counselling, if no such result is documented.
- Obtain written informed consent from the subject prior to the initiation of any screening procedures.
- Provide adequate counselling regarding risks and benefits of breastfeeding.
- Obtain a relevant medical history for the duration of the pregnancy.
- Obtain a HIV related and medication history from the patient with particular reference to prior antiretroviral drug exposure.
- Record patient demographic data.
- Perform targeted physical examination which will include measurements of blood pressure, pulse rate, temperature and weight, and examinations of the following systems: Cardiovascular, Respiratory, Central Nervous System, Abdominal, ENT and the integument.
- Perform laboratory tests : serum creatinine, ALT, AST, ALP, Total Bilirubin, amylase, full blood count, CD4+ count, HIV RNA PCR (viral load).
- Collect and submit blood sample for resistance and other HIV-1 virologic testing once HIV result confirmed as being positive.
- Record all concomitant therapies and adverse events.

**Visit 2: Day 0 (enrollment, labour and delivery)**

- Review inclusion and exclusion criteria.
- Patients will be randomised and receive a patient number when they present in labour after 36 weeks gestation.
- Collect blood for CD4 and viral load( HIV RNA PCR).
- Collect and submit blood sample for resistance and other HIV-1 virologic testing.
- Record any adverse events and concomitant therapies.
- Administer study medication and record medication administered in the CRF.
- Record labour and delivery history details in CRF.

**Visit 3: (day 1-2)**

- This will take place 24-48 hours after delivery.
- Record post delivery history and any HIV related physical signs and symptoms in the mother.
- Perform targeted physical examination (as with visit 1).
- Record any adverse events and concomitant therapy.
- Perform laboratory tests as with visit 1 (screening visit).
- Collect blood sample for resistance and other HIV-1 virologic testing.
- Dispense further study medication to mother (depending on treatment arm) if she is to be discharged as indicated in section 6.1 and record in the CRF.

**Visit 4: (14 days post partum: ± 2 days)**

- Record any adverse events which have occurred in the mother as well as concomitant therapy used.
- Collect blood from mother for laboratory tests as with screening visit, including blood sample for resistance and other HIV-1 virologic testing.
- Collect all study medication used by the mother and assess subject compliance.

**Visit 5 : (day 42: ± 6 days)**

- Assess mother for any HIV related signs and symptoms
- Perform a targeted physical examination
- Record any adverse events and concomitant therapies in the mother
- Perform laboratory tests as with previous visits , including resistance and other HIV-1 virologic testing on the mother. If the mother fails to demonstrate any resistance mutations on the blood sample from this visit, this then concludes her further participation in the study.

**Visits 6 and 7 : (day 90 and 168: ± 10 days)**

All the subjects for further evaluation, should be HIV positive with demonstrated resistance mutations

- Assess mother for any HIV related signs and symptoms
- Perform a targeted physical examination as before
- Record any adverse events and concomitant therapies
- Perform laboratory tests for CD4 , viral load , resistance and other HIV-1 virologic testing

**Visit 8: (final visit day 336: ± 14 days)**

- Assess mother for any HIV related signs and symptoms
- Perform a targeted physical examination
- Record any adverse events and concomitant therapies
- Perform laboratory tests for CD4 , viral load , resistance and other HIV-1 virologic testing
- This visit concludes participation in the study by the mother

**INFANT:**

**Visit 2 : Delivery (Day 0)**

- No assessments.

**Visit 3 : (day 1-2)**

- Collect patient demographic data.
- Review eligibility criteria.
- Record neonatal history and perform a complete physical examination , recording all abnormalities in the CRF.
- Perform the following laboratory tests on the neonate : Full blood count, creatinine , Total bilirubin, AST, ALT ,ALP , amylase, HIV DNA PCR, HIV RNA PCR.
- Collect sample from neonate and store for possible resistance testing and other HIV-1 virologic testing pending result of DNA PCR.
- Record any adverse events and concomitant therapy in the neonate.
- Record the method of infant feeding to be used.
- Administer randomised study medication to infant and record medication in the CRF.
- Dispense any further study medication, to be used by neonate as indicated in section 6.1, to mother and record in the CRF such dispensed medication.

**Visit 4 : ( 14 days post partum) ± 2days**

- Perform a targeted physical examination on the infant
- Record all adverse events experienced by the infant as well as concomitant therapies
- Collect blood for laboratory tests as with visit 3 above
- Collect a blood sample for resistance testing and other HIV-1 virologic testing as with visit 3 above
- Collect all study medication used by infant and assess infant compliance
- Record the method of infant feeding or any change therein if applicable

**Visit 5 : (day 42 ) ± 6 days**

- Perform a targeted physical examination on the infant
- Assess the infant for HIV related signs and symptoms
- Record any adverse events and all concomitant therapies in the infant
- Record the method of infant feeding
- Collect blood for laboratory tests as with visit 3 above
- Collect a blood sample for resistance and other HIV-1 virologic testing if the infant HIV DNA PCR is positive
- This visit will conclude the infant participation if the infant is HIV negative or if the infant is HIV positive ( see section 5.1.2 for definitions) but no genotypic resistance mutations are identified .

**Note : For infants who test HIV DNA PCR positive for the first time at this visit , a follow up visit 5.1 , must be arranged in an attempt to clarify their HIV status.**

**Visit 5.1 (day49) ± 6 days**

- Collect and submit blood sample for repeat HIV DNA PCR
- Collect blood sample for viral load ,(HIV RNA PCR), and resistance and other HIV-1 virologic testing. **Note** only submit these samples for testing if the HIV DNA PCR from this visit is positive.
- If no genotypic resistance mutations are identified this concludes the infants participation in this study

**Visit 6 (day 90) ± 10 days**

- Perform a targeted physical examination on the infant
- Assess the infant for HIV related signs and symptoms
- Record any adverse events and all concomitant therapies in the infant
- Record the method of infant feeding
- Collect blood samples for viral load measurement(HIV RNA PCR) and resistance and other HIV-1 virologic testing

**Visits 7 (day 168) ± 10 days**

- Perform a targeted physical examination on the infant
- Record any adverse events and all concomitant therapies in the infant
- Record the method of infant feeding
- Collect blood samples for viral load measurement (HIV RNA PCR) and resistance and other HIV-1 virologic testing

**Visit 8 : ( final visit day 336) ± 14 days**

- Perform a targeted physical examination on the infant
- Assess the infant for HIV related signs and symptoms
- Record any adverse events and all concomitant therapies in the infant
- Record the method of infant feeding
- Collect blood samples for viral load measurement (HIV RNA PCR) and resistance and other HIV-1 virologic testing
- This visit concludes participation in the study by the infant

## ADHERENCE TO PROTOCOL

### Protocol violations are any deviations from the protocol, which include, but are not limited to, the ingestion of disallowed medication or non-compliance with study medication or visit procedures and schedules.

### 6.3.1 Visit schedule

Patients should be instructed to return for their scheduled appointments. If a visit must be rescheduled, subsequent visits should follow the original visit schedule as set out in the protocol flow chart.(i.e. the active treatment period is not shortened or lengthened by rescheduling of visits). For reasons of flexibility due to unforeseen circumstances a period of 2 days for visit 4 , 6 days for visit 5 and 5.1, 10 days for visits 6 and 7 and 14 days for visit 8, will be allowed as the time frame for rescheduling visits. Interim visits to assess safety (as with adverse events) can be conducted at the investigators discretion.

### 6.3.2 Criteria and rules for stopping subject treatment

#### Patients may be discontinued at any time during the study for the following reasons :

##### Intercurrent disease or necessary therapy not allowing further participation.

##### Withdrawal of patients consent.

##### If the trial is discontinued prematurely at the discretion of the Sponsor.

##### Any other reason as determined by the investigator in collaboration with the Clinical Monitor.

**6.3.3 Dropouts and withdrawals**

Patients have the right to withdraw from the study at any time for any reason.

The investigator has the right to remove patients from the study for the reasons

described in section 6.3.2, for administrative or for other reasons. It is understood by all concerned that an excessive rate of withdrawals can render the study uninterpretable; therefore, unnecessary withdrawal of patients should be avoided. Any patient withdrawal should be discussed with the Boehringer Ingelheim clinical monitor prior to the patient leaving the study.

If a patient is removed from the study due to an adverse event or an abnormal laboratory test result, the patient must be followed until complete resolution of the event or laboratory abnormality, or for follow up deemed sufficient by the e investigator and the Boehringer Ingelheim clinical monitor.

For all randomised patients, the date of the last dose of study drug and the reason for patient discontinuation must be recorded in the CRF. All patients who prematurely discontinue randomised treatment should have a complete set of visit 5 clinical evaluations as described in section 6.2 , study procedures at each visit.

Any adverse events, which are continuing at the time the patient discontinues from the study, must be followed until deemed sufficient by the investigator and the Boehringer Ingelheim clinical monitor or until the event has resolved . In addition should the investigator become aware of any serious adverse drug related event after the observational period , this should be reported to the Sponsor.

# 7. statistics

## 7.1 STATISTICAL DESIGN / MODEL

Statistical models for primary analysis will compare treatment groups without adjustment for covariates.

Timing of maternal exposure, relative to delivery, is not expected to be a factor in selection for nevirapine resistance-associated mutations.

## 7.2 NULL AND ALTERNATIVE HYPOTHESES

The null hypothesis is that differences in development of resistance-associated mutations are a result of random variation and that the expected proportion of mothers developing one or more resistance associated reverse transcriptase (RT) mutation is the same for all three treatment groups. Alternate hypotheses, tested sequentially, are that 7 day Combivir® treatment has a lower proportion of mothers developing reverse transcriptase mutations than no Combivir®, followed by 4 day Combivir® treatment has a lower proportion than no Combivir®.

## 7.3 PLANNED ANALYSES

### 7.3.1 Primary analyses

Treatment groups will be compared using the Fisher's Exact test to perform pairwise comparisons. All mothers with genotypic testing results at least 2 weeks and no more than 8 weeks after delivery will be included in the analysis.

### 7.3.2 Secondary analyses

Logistic regression methods will be applied to explore the relationship between development of nevirapine-associated mutations and baseline maternal CD4 count, viral load, viral subtype, and treatment. Infection of infants will be estimated by period and overall, using the Kaplan-Meier algorithm. Similarly, infection-free survival will be estimated. Risk factors including baseline maternal CD4 count and HIV-1 RNA level, emergency caesarean-section, breast-feeding, and timing of nevirapine dose relative to delivery will be explored with logistic regression models.

### 7.3.3 Safety analyses

All mothers are being treated with nevirapine. Adverse events and laboratory results for all treatment groups will be presented descriptively. Special attention will be given to any rashes occurring in the 14 days after receipt of the nevirapine dose and to haemoglobin results in those mothers receiving Combivir®.

### 7.3.4 Interim analyses

No interim analyses are planned.

## 7.4 HANDLING OF MISSING DATA

Follow-up through the 6 week visit is critical to evaluability of selection for resistance. Mothers who miss this visit and return later can be evaluated if they have nevirapine-associated mutations. If they do not have nevirapine-associated mutations, then they may have had them and lost them or they may have never developed mutations. Any mothers who have missed the 6 week visit will be evaluated without knowledge of treatment assignment to determine how they can be handled in analyses.

Similarly, follow-up is critical to evaluability of the infants with respect to infection. All available data will be used in estimation of rates of infection.

## 7.5 RANDOMISATION

One randomisation sequence , maintaining a one to one balance between treatment arms will be generated .Treatment assignments will be maintained blinded until randomisation, only unblinding each mother/infant pair immediately before dosing of the mother. Mothers will be assigned to treatment with 7 days Combivir®, 4 days Combivir®, or no Combivir®.

## 7.6 SAMPLE SIZE ISSUES

With a different genotyping process and different subtypes of HIV-1, resistance-associated mutations were seen in approximately 20% of mothers. With a different dosing regimen, 2 doses of nevirapine, HIV-1 resistance-associated mutations were seen in approximately 60% of mothers. Sample size of 80 evaluable mothers per treatment group was selected based on considering control rates of 20-30%.At 20% rate for the nevirapine-only arm , 80% power is achieved for an alpha-0.05(2-sided) test if Combivir® results in an 80% reduction to 4% in the proportion of mothers with nevirapine-associated mutations. At 25% rate for the nevirapine-only arm , 92% power is achieved for an alpha-0.05(2-sided) test if Combivir® results in an 80% reduction to 5% in the proportion of mothers with nevirapine-associated mutations. At 30% rate for the nevirapine-only arm , 85% power is achieved for an alpha-0.05(2-sided) test if Combivir® results in a 66% reduction to 10% in the proportion of mothers with nevirapine-associated mutations.

# 8. ADMINISTRATIVE MATTERS

The trial will be carried out in compliance with the protocol, the principles laid down in the Declaration of Helsinki, last revised version, in accordance with the ICH Harmonised Tripartite Guideline for Good Clinical Practice (GCP) and in accordance with applicable regulatory requirements.

## 8.1 ETHICS

### 8.1.1 Institutional Review Board or Independent Ethics Committee

The trial will not be initiated before the protocol and informed consent and subject information form have been reviewed and received approval / favourable opinion from the local Institutional Review Board (IRB) or an Independent Ethics Committee (IEC). Should a protocol amendment be made that needs IRB / IEC approval, the changes in the protocol will not be instituted until the amendment and revised informed consent (if appropriate) has been reviewed and received approval / favourable opinion from the local IRB or IEC. A protocol amendment intended to eliminate an apparent immediate hazard to subjects may be implemented immediately providing that the appropriate regulatory authorities and IRB / EC are notified as soon as possible and an approval is requested. Protocol amendments only for logistical or administrative changes may be implemented immediately; the IRB /IEC needs to be informed only.

The constitution of the IRB or IEC must meet the requirements of the participating country(ies). A list of the IRB / IEC members, with names and qualifications, will be requested. If such a list is unavailable, the investigator must provide the name and address of the IRB/IEC along with a statement from the IRB/IEC that it is organised according to GCP and the applicable laws and regulations. The IRB or IEC must also perform all duties outlined by the requirements of the participating country(ies). For studies conducted under a US IND, the requirements outlined in the US Code of Federal Regulations must also be met.

### 8.1.2 Informed Consent and Subject Information

Prior to subject participation in the trial, written informed consent will be obtained from each subject (or the subject’s legally accepted representative) according to the regulatory and legal requirements of the participating country. Each signature must be dated by each signatory and the informed consent and any additional subject information form retained by the investigator as part of the study records. A signed copy of the informed consent and any additional subject information must be given to each subject or the subject’s legally authorised representative.

The subject must be informed that his / her medical records may be examined by authorised monitors or Clinical Quality Assurance auditors appointed by Boehringer Ingelheim, by appropriate IEC / IRB members and by inspectors from regulatory authorities.

Should a protocol amendment be made, the subject consent form and subject information form may need to be revised to reflect the changes to the protocol. It is the responsibility of the investigator to ensure that an amended consent form is reviewed and received approval / favourable opinion from the IRB or IEC, and that it is signed by all subjects subsequently entered in the trial and those currently in the trial, if affected by the amendment.

## 8.2 RECORDS

### 8.2.1 Drug Accountability

Drug supplies, which will be provided by the sponsor, must be kept in a secure, limited access storage area under the storage conditions defined by the sponsor. Where necessary, a temperature log must be maintained to make certain that the drug supplies are stored at the correct temperature.

The investigator and/or pharmacist must maintain records of the product’s delivery to the trial site, the inventory at the site, the use by each subject, and the return to the sponsor or alternative disposition of unused product(s). These records will include dates, quantities, batch/serial numbers, expiration dates (if applicable), and the unique code numbers assigned to the investigational product(s) and trial subjects. Investigators will maintain records that document adequately that the subjects were provided the doses specified by the protocol and reconcile all investigational product(s) received from the sponsor. At the time of return to the sponsor, the investigator must verify that all unused or partially used drug supplies have been returned by the clinical trial subject and that no remaining supplies are in the investigator’s possession.

### Emergency code break

### Not applicable.

### 8.2.3 Case Report Forms

Case report forms for individual subjects will be provided by the sponsor. A copy remains with the investigator as a permanent record, and the original will be returned to the sponsor.

Case report forms are used to record clinical trial data and are an integral part of the trial and subsequent reports. The case report forms, therefore, must be legible and complete. All forms must be filled in using a black ballpoint pen. Errors must be lined out but not obliterated and the correction inserted, initialled and dated.

The investigator must sign a declaration ensuring accuracy of data recorded in the case report forms.

Case reports forms must be kept current to reflect subject status at each phase during the course of trial. Subjects are not to be identified on the case report form by name. Appropriate coded identification (e.g. Subject Number) and subject initials must be used. The investigator must make a separate confidential record of these details (subject identification code list) to permit identification of all subjects enrolled in a clinical trial in case follow-up is required.

Relevant medical history prior to enrollment will be documented at the baseline visit. Thereafter during the trial narrative statements relative to the subject’s progress during the trial will be maintained. See also 8.2.4.

The investigator will be responsible for retaining all records pertaining to the trial as specified in the contract appropriate contract.

### 8.2.4 Source documents

Source documents provide evidence for the existence of the subject and substantiate the integrity of the data collected. Source documents are filed at the investigator’s site. Data reported on the Case Report Forms that are derived from source documents must be consistent with the source documents or the discrepancies must be explained.

The investigator may need to request previous medical records or transfer records, depending on the trial; also current medical records – not just shadow charts – must be available.

The following data to be reported on the CRF should be included and derived from the source documents:

Subject identification (initials, gender, data of birth)
Subject participation in the trial (substance, trial number, patient number, date informed consent given)
Dates of subject’s visits
Medical history
Medication history
Adverse events (AE onset and end)
Serious adverse events (SAE onset and end)
Originals or copies of laboratory results
Originals or copies of X-rays and Ultrasound findings, ECG results, EEG results, pneumological findings, endoscopic findings and other results based on hard copies of medical machinery
Conclusion of subject’s participation in the trial.

### 8.2.5 Direct access to source data / documents

The investigator / institution will permit trial related monitoring, audits, IRB / IEC review and regulatory inspection, providing direct access to all related source data / documents. Case report forms and all source documents, including progress notes and copies of laboratory and medical test results must be available at all times for review by the sponsor’s clinical trial monitor and inspection by health authorities (e.g., FDA). The CRA/on‑site monitor may review all case report forms, and written informed consents. The accuracy of the data will be verified by reviewing the documents described in Section 8.2.4.

## 8.3 QUALITY ASSURANCE AUDIT

A quality assurance audit of this trial may be conducted by the sponsor or sponsor’s designees. The quality assurance auditor will have access to all medical records, the investigator’s trial related files and correspondence, and the informed consent documentation that is relevant to this clinical trial.

## 8.4 PROCEDURES

### 8.4.1 Adverse Events

All adverse events occurring during the course of the clinical trial (i.e., from signing the informed consent onwards) will be collected, documented and reported to the sponsor by the investigator according to the specific definitions and instructions detailed in the “Adverse Event Reporting” section of the Investigator Site File.

An adverse event is defined as any untoward medical occurrence in a clinical investigation subject administered a pharmaceutical product and which does not necessarily have to have a causal relationship with this treatment.

A serious adverse event is defined as any adverse event which results in death, is immediately life-threatening, results in persistent or significant disability / incapacity, requires or prolongs patient hospitalisation, is a congenital anomaly / birth defect, is to be deemed serious for any other reason representing a significant hazard, which is comparable to the aforementioned criteria.

All adverse events, serious and non-serious, will be fully documented on the appropriate case report form(s). For each adverse event, the investigator will provide the onset, duration, intensity, treatment required, outcome and action taken with the investigational product. The investigator will determine the relationship of the investigational product to all adverse events as defined in the “Adverse Event Reporting” Section of the Investigator Site File.

The investigator has the responsibility to report adverse events occurring in a period after a subject completes the trial, as defined and described in the “Adverse Event Reporting” section of the Investigator Site File.

### 8.4.2 Emergency procedures

Any serious or significant adverse event, whether or not considered related to the investigational product, and whether or not the investigational product has been administered, must be reported immediately by telephone / fax to the sponsor. Details regarding this reporting procedure are provided in the Investigator Site File.

Following all such telephone / fax reports the Clinical Monitor must provide a written report of the adverse event and any sequelae to Corporate Drug Safety according to the appropriate Corporate SOP. These narratives, which confirm the information collected by telephone and may give additional information not available at the time of the initial report.

## 8.5 RULES FOR AMENDING PROTOCOL

All amendments must be documented, dated and signed by all signatories (or their successors) of the original protocol.

## 8.6 DISCONTINUATION OF THE TRIAL BY THE SPONSOR

Boehringer Ingelheim reserves the right to discontinue this trial at any time for failure to meet expected enrollment goals, for safety or any other administrative reasons. The investigator will be reimbursed for reasonable expenses incurred if it is necessary to terminate the trial.

## 8.7 STATEMENT OF CONFIDENTIALITY

Individual subject medical information obtained as a result of this study is considered confidential and disclosure to third parties is prohibited with the exceptions noted below. Subject confidentiality will be further ensured by utilising subject identification code numbers to correspond to treatment data in the computer files.

Such medical information may be given to the subject’s personal physician or to other appropriate medical personnel responsible for the subject’s welfare.

Data generated as a result of this trial are to be available for inspection on request by the participating physicians, the sponsor’s representatives, by the IRB or IEC and the regulatory health authorities.

## 8.8 PUBLICATION POLICY

Boehringer Ingelheim is as much as possible dedicated to support process of free exchange of relevant scientific information. Any publication of the result of this trial must be consistent with the Boehringer Ingelheim publication policy. The rights of the investigator and of the sponsor with regard to publication of the results of this trial are described in the investigator contract.

# 9. SIGNATURE PAGE(S)

| Trial Clinical Monitor: |  |  |  |  |
| --- | --- | --- | --- | --- |
| Name |  | Date |  | John Steytler |
| Organisation/Department |  |  |  | Boehringer Ingelheim (Pty) Ltd / Medical Dept. |
| Trial Statistician: (indicate early information on signature, if applicable) |  |  |  |  |
| Name |  | Date |  | David Hall |
| Organisation/Department |  |  |  | Boehringer Ingelheim Pharmaceuticals, Inc./ Biometrics and Data Management |
| Medical Director: |  |  |  |  |
| Name |  | Date |  | Lynette Boshoff |
| Organisation/Department |  |  |  | Boehringer Ingelheim (Pty) Ltd / Medical Dept. |
| Team Member Medicine: (indicate early information on approval, if applicable |  |  |  |  |
| Name |  | Date |  | Patrick Robinson |
| Organisation/Department |  |  |  | Boehringer Ingelheim Pharmaceuticals, Inc. / Clinical Research |
|  |  |  |  |  |
| I hereby certify that I agree to adhere to the protocol and to all the documents referenced in the protocol. | | | | |
| Investigator: |  |  |  |  |
| Name |  | Date |  |  |
| Organisation/Department |  |  |  |  |

# references

1. Richman D and Staszewski S . A practical guide to HIV drug resistance and its implications for antiretroviral treatment strategies. 2^ND^ Edition. 2000 .International Medical Press Ltd. ; pp 17 – 20.

2. R01-1178: Mock PA , Schaffer N , Bhadrakom C , Siriwasin W , Chotpitayasunondh T, Chearskul S , Young NL , Roongpisuthipong A , Chinayon P , Kalish ML ,Parekh B , Mastro TD., Bangkok Collaborative Perinatal HIV Transmission Study Group. Maternal viral load and timing of mother-to-child HIV transmission , Bangkok, Thailand. **AIDS**(Phila) 1999; 13 : 407-414

3. P99-02572 : Guay LA , Musoke P , Fleming T , Bagenda D , Allen M, Nakabiito C, et al. Intrapartum and neonatal single dose nevirapine compared with zidovudine for prevention of mother to child transmission of HIV-1 in Kampala, Uganda : HIVNET 012 randomised trial. **Lancet** 1999 ; 354 (9181) : 795-802.

1. P99-12030 : Richman DD. Drug resistance and its implications in the management of HIV infection. **Antiviral Therapy** 2 (Supplement 4) , 41-58 (1997).

5. P90-4449 : Merluzzi VJ, Hargrave KD, Lababia M, et al. Inhibition of HIV-1 replication by a non–nucleoside reverse transcriptase inhibitor. **Science** 1990 ;250 : 1411-1413.

6. R00-1002 : Package insert for Viramune® Tablets and oral suspension, U.S., revised 08 July 1999 and implemented 26 October 1999.

7. P98-7078 : Mirochnick M , Fenton T , Gagnier P , Pav J, Gwynne M , Siminski S, et al. Pharmacokinetics of nevirapine in human immunodeficiency virus type 1-infected pregnant women and their neonates. **J Infect Dis** 1998 ;178 : 368-374.

8. R01–1197 : VIRAMUNE®(nevirapine) Tablets and Oral Suspension Package Insert and Patient Information. Boehringer Ingelheim Roxanne Laboratories, Revised 11/10/00 ; 4077435/US/3.

9. U91-0312 : Investigator’s Brochure Nevirapine (BI–RG-587) VIRAMUNE®.Boehringer Ingelheim , Revised March 2000.

10. P93-7261 : Richman D, Shih CK , Lowy I , Rose J, Prodanovich P, Goff S, Griffin J Human immunodeficiency virus type 1 mutants resistant to nonnucleoside inhibitors of reverse transcriptase arise in tissue culture. **Proc Natl Acad Sci USA** 88(24) , 11241-11245(1991).

11. P94–5965 : Richman DD, Havlir D, Corbeil J , et al. Nevirapine resistance mutations of human immunodeficiency virus type 1 selected during therapy. **J Virol** 68 (3) , 1660 –1666 (1994)

12. P94-6218 : Richman DD. Resistance of clinical isolates of human immunodeficiency virus to antiretroviral agents. Antimicrob Agents Chemother 37(6) , 1207-1213 (1993)

13. P94-6421 : Smerdon SJ , Jäger J, Wang J , Kohlstaedt LA , Chirino AJ , Friedman JM, et al. Structure of the binding site for non-nucleoside inhibitors of the reverse transcriptase of human immunodeficiency virus type 1. **Proc Natl Acad Sci** 1994 ; 91 : 3911-3915.

14. P99-00967: Musoke P , et al. A phase I/II Study of Safety and Pharmacokinetics of Nevirapine in HIV-1 infected Pregnant Ugandan Women and Their Neonates(HIVNET006). **AIDS** 1999 ; 13 : 479-486

15. P01-06388: Eshleman SH , Mracna M ,Guay L , Deseyve M , Cunningham S , Musoke P , et al. Selection of Nevirapine Resistance (NVPR) Mutations in Ugandan Women and Infants Receiving nevirapine Prophylaxis To Prevent HIV–1 Vertical Transmission (HIVNET012). **8^th^ Conference on Retroviruses and Opportunistic Infections** , Chicago.February 2001 ; Abstract 516.

16. U01-3282 : Boshoff L, Hall D, Carr P , Robinson P , Frank M, Gigliotti M , Hu P. **Clinical Trial Report BI 1100.1287.** October 2001.A prospective randomised open label clinical trial to determine the efficacy of nevirapine , compared with a combination of ZDV + 3TC, in decreasing the peripartum mother to child transmission of HIV. Women who present after 38 weeks gestation or are in labour after 35 weeks gestation and who are antiretroviral naïve, will be included.

17. P00-14835 : Joly V , Yeni P . Non-nucleoside reverse transcriptase inhibitors. Ann Med Interne 2000 ; 151: (4) , 260-267

# Appendices

**Appendix 1 :**

# Viramune® (Nevirapine) Clinical Trials Hepatotoxicity Management Schema

Introduction :

The first 12 weeks of therapy with Viramune® are a critical period during which intensive monitoring of patients is required to detect potentially life-threatening hepatic events and skin reactions. Liver function tests (LFTs) should be performed if a patient experiences signs or symptoms suggestive of hepatitis and/or hypersensitivity reaction. Clinicians and patients should be vigilant for the appearance of signs or symptoms of hepatitis, such as fatigue, malaise, anorexia, nausea, jaundice, bilirubinuria, acholic stools, liver tenderness or hepatomegaly. The diagnosis of hepatotoxicity should be considered in this setting, even if liver function tests are initially normal or alternative diagnoses are possible.

Inclusion criteria may permit enrollment of anyone with asymptomatic hepatic history and LFTs < Grade 3. Patients with asymptomatic chronic Hepatitis B or C are generally not excluded but may be at increased risk regardless of antiretroviral regimen.²

Exclusion criteria should prohibit enrollment of anyone with clinical symptomatic hepatitis, regardless of liver function test grade, or if asymptomatic with Grade 3 or greater.

Study patients who develop, from baseline, asymptomatic Grade 1 or 2 AST / ALT may continue with study treatment but should be carefully observed and evaluated.

Study patients who develop, from baseline, asymptomatic Grade 3 or 4 AST / ALT must immediately discontinue study treatment and be carefully observed and evaluated.^3^

Any study patient with clinical symptomatic hepatitis or clinical evidence of a hypersensitivity reaction (HSR)³, regardless of LFT grade, should immediately discontinues study treatment and be carefully observed and evaluated.^3^

Viramune® Package Insert.

² Asymptomatic elevations in GGT or bilirubin levels, without elevations in other liver function tests, are not a contraindication to beginning or continuing Viramune therapy.

³Hypersensitivity Reactions (HSR) characterized by rash, constitutional findings, and organ dysfunction.

| LIVER TRANSAMINASE LEVELS (LFTs) | | | | |
| --- | --- | --- | --- | --- |
|  | Grade 1 | Grade 2 | Grade 3 | Grade 4 |
| AST (SGOT) | 1.25-2.5 x ULN | >2.5-5.0 x ULN | >5.0-7.5 x ULN | >7.5 x ULN |
| ALT (SGPT) | 1.25-2.5 x ULN | >2.5-5.0 x ULN | >5.0-7.5 x ULN | >7.5 x ULN |

## APPEndix table 2

IF DESPITE THE BELOW GUIDELINES, YOU ARE IN DOUBT ABOUT WHAT YOU SHOULD DO WITH YOUR PATIENT, PLEASE CONSULT WITH A REPRESENTATIVE OF THE SPONSOR (please mention the patient number and short history)

| **RASH MANAGEMENT GUIDELINES** | |
| --- | --- |
| **Rash Description** | **Action with nevirapine** |
| GRADE I or IIa without associated constitutional findings  Mild/Moderate Rash  (may include pruritus)  Erythema  Diffuse erythematous macular or maculopapular cutaneous eruption | Can continue dosing without interruption.  If rash occurs during lead-in, the dose should not be escalated until the rash resolves  If nevirapine is interrupted for >7 days, reintroduce with 200mg/d lead-in |
| GRADE IIb without associated constitutional findings   - Urticaria | nevirapine can be continued as above; however, IF nevirapine is interrupted, do not reintroduce |
| GRADE I, IIa or IIb RASH with associated constitutional findings such as:  Fever >39ºC  Blistering  Oral lesions  Conjunctivitis  Facial edema  Myalgia/arthralgia  General malaise | Immediate and permanent discontinuation |
| GRADE III or IV with or without associated constitutional findings   - Severe Rash   Extensive erythematous or maculopapular rash or moist  desquamation  Angioedema  Serum sickness-like reactions  Stevens-Johnson syndrome (SJS)  Toxic epidermal necrolysis (TEN) | Immediate and permanent discontinuation |

Any rash with associated constitutional

findings and evidence of acute organ dysfunction such as hepatitis,

granulocytopenia, eosinophilia, or renal dysfunction.

Immediate and permanent discontinuation.

## APPENDIX 3 :

## ACTG Table for grading adult adverse experiences

ABBREVIATIONS: Abbreviations utilized in the Table:

ULN = Upper Limit of Normal LLN = Lower Limit of Normal

Rx = Therapy Req = Required

Mod = Moderate IV = Intravenous

ADL = Activities of Daily Living Dec = Decreased

ESTIMATING SEVERITY GRADE

For abnormalities NOT found elsewhere on the Tox Table use the scale below to estimate grade of severity:

**GRADE 1 Mild** Transient or mild discomfort; no limitation in activity; no medical intervention/therapy required

**GRADE 2 Moderate** Mild to moderate limitation in activity - some assistance may be needed; no or minimal medical intervention/therapy required

**GRADE 3 Severe** Marked limitation in activity, some assistance usually required; medical intervention/therapy required, hospitalizations possible

**GRADE 4 Life-** Extreme limitation in activity, significant assistance

**threatening** required; significant medical intervention/therapy required, hospitalization or hospice care probable

SERIOUS OR LIFE-THREATENING AEs

ANY clinical event deemed by the clinician to be serious or life‑threatening should be considered a grade 4 adverse experience. Clinical events considered to be serious or life‑threatening include, but are not limited to:

seizures, coma, tetany, diabetic ketoacidosis, disseminated intravascular coagulation, diffuse petechiae, paralysis, acute psychosis

MISCELLANEOUS

> When two values are used to define the criteria for each parameter, the lowest values will appear first.

> Parameters are generally grouped by body system.

> Some protocols may have additional protocol specific grading criteria.

**PARAMETER GRADE 1 GRADE 2 GRADE 3 GRADE 4**

MILD MODERATE SEVERE POTENTIALLY LIFE THREATENING

HEMATOLOGY

Hemoglobin 8.0 g/dL - 9.4 g/dl 7.0 g/dL - 7.9 g/dL 6.5 g/dL - 6.9 g/dL <6.5 g/dL

Absolute Neutrophil 1000 - 1500/mm^3^ 750 - 999/mm^3^ 500 - 749/mm^3^ <500/mm^3^

Count

Platelets 75,000 - 99,000/mm^3^ 50,000 - 74,999/mm^3^ 20,000 - 49,999/mm^3^ <20,000/mm^3^

Prothrombin Time (PT) >1.0 - 1.25 X ULN >1.25 - 1.5 X ULN >1.5 - 3.0 X ULN >3 X ULN

PTT >1.0 - 1.66 x ULN >1.66 - 2.33 x ULN >2.33 - 3.0 x ULN >3.0 x ULN

Methemoglobin 5.0 - 10.0% 10.1 - 15.0% 15.1 - 20.0% >20%

CHEMISTRIES

SODIUM

Hyponatremia 130 - 135 meq/L 123 - 129 meq/L 116 - 122 meq/L <116 meq/L

Hypernatremia 146 - 150 meq/L 151 - 157 meq/L 158 - 165 meq/L >165 meq/L

POTASSIUM

Hyperkalemia 5.6 - 6.0 meq/L 6.1 - 6.5 meq/L 6.6 - 7.0 meq/L >7.0 meq/L

Hypokalemia 3.0 - 3.4 meq/L 2.5 - 2.9 meq/L 2.0 - 2.4 meq/L <2.0 meq/L

PHOSPHATE

Hypophosphatemia 2.0 - 2.4 mg/dL 1.5 - 1.9 mg/dL 1.0 - 1.4 mg/dL <1.0 mg/dL

**PARAMETER GRADE 1 GRADE 2 GRADE 3 GRADE 4**

MILD MODERATE SEVERE POTENTIALLY LIFE THREATENING

CALCIUM - (corrected for albumin)

Hypocalcemia 7.8 - 8.4 mg/dL 7.0 - 7.7 mg/dL 6.1 - 6.9 mg/dL <6.1 mg/dL

Hypercalcemia 10.6 - 11.5 mg/dL 11.6 - 12.5 mg/dL 12.6 - 13.5 mg/dL >13.5 mg/dL

MAGNESIUM

Hypomagnesemia 1.2 - 1.4 meq/L 0.9 - 1.1 meq/L 0.6 - 0.8 meq/L <0.6 meq/L

BILIRUBIN

Hyperbilirubinemia >1.0 - 1.5 x ULN >1.5 - 2.5 x ULN >2.5 - 5 x ULN >5 x ULN

GLUCOSE

Hypoglycemia 55 - 64 mg/dL 40 - 54 mg/dL 30 - 39 mg/dL <30 mg/dL

Hyperglycemia 116 - 160 mg/dL 161 - 250 mg/dL 251 - 500 mg/dL >500 mg/dL

(nonfasting and

no prior diabetes)

Triglycerides ________ 400 - 750 mg/dL 751 - 1200 mg/dL >1200 mg/dL

Creatinine >1.0 - 1.5 x ULN >1.5 - 3.0 x ULN >3.0 - 6.0 x ULN >6.0 x ULN

URIC ACID

Hyperuricemia 7.5 - 10.0 mg/dL 10.1 - 12.0 mg/dL 12.1 - 15.0 mg/dL >15.0 mg/dL

**PARAMETER GRADE 1 GRADE 2 GRADE 3 GRADE 4**

MILD MODERATE SEVERE POTENTIALLY LIFE THREATENING

LIVER TRANSAMINASE (LFTs)

AST (SGOT) 1.25 - 2.5 x ULN >2.5 - 5.0 x ULN >5.0 - 10.0 x ULN >10.0 x ULN

ALT (SGPT) 1.25 - 2.5 x ULN >2.5 - 5.0 x ULN >5.0 - 10.0 x ULN >10.0 x ULN

GGT 1.25 - 2.5 x ULN >2.5 - 5.0 x ULN >5.0 - 10.0 x ULN >10.0 x ULN

Alk Phos 1.25 - 2.5 x ULN >2.5 - 5.0 x ULN >5.0 - 10.0 x ULN >10.0 x ULN

PANCREATIC ENZYMES

Amylase >1.0 - 1.5 x ULN >1.5 - 2.0 x ULN >2.0 - 5.0 x ULN >5.0 x ULN

Pancreatic amylase >1.0 - 1.5 x ULN >1.5 - 2.0 x ULN >2.0 - 5.0 x ULN >5.0 x ULN

Lipase >1.0 - 1.5 x ULN >1.5 - 2.0 x ULN >2.0 - 5.0 x ULN >5.0 x ULN

CARDIOVASCULAR

Cardiac Arrhythmia ________ Asymptomatic; Recurrent/persistent Unstable dysrhythmia,

transient dysrhythmia, dysrhythmia; symptomatic hospitalization and no Rx req Rx req Rx req

Hypertension Transient, increase Recurrent; chronic Acute Rx req; outpatient Hospitalization req

>20 mm/Hg; no Rx increase >20 mm/Hg, hospitalization possible

Rx req

Hypotension Transient orthostatic Symptoms correctable IV fluid req, no Hospitalization req

hypotension, no Rx with oral fluid Rx hospitalization req

Pericarditis Minimal effusion Mild/mod asymptomatic Symptomatic effusion, Tamponade OR pericardi-

effusion, no Rx pain, EKG changes iocentesis OR surgery req

Hemorrhage, blood ________ Mildly symptomatic, Gross blood loss OR Massive blood loss OR

Loss no Rx required 1-2 units transfused >2 units transfused

**PARAMETER GRADE 1 GRADE 2 GRADE 3 GRADE 4**

MILD MODERATE SEVERE POTENTIALLY LIFE THREATENING

GASTROINTESTINAL

Nausea Mild OR transient; Mod discomfort OR Severe discomfort OR Hospitalization req

reasonable intake intake decreased minimal intake for

maintained for <3 days >3 days

Vomiting Mild OR transient; Mod OR persistent; Severe vomiting of all Hypotensive shock OR

2-3 episodes per day 4-5 episodes per day; food/fluids in 24 hrs OR hospitalization req for

OR mild vomiting OR vomiting lasting orthostatic hypotension IV Rx req

lasting <1 week >1 week OR IV Rx req

Diarrhea Mild OR transient; Mod OR persistent; Bloody diarrhea; OR Hypotensive shock OR

3-4 loose stools per 5-7 loose stools per orthostatic hypotension hospitalization req

day OR mild diarrhea day OR diarrhea OR >7 loose stools/day

lasting <1 week lasting >1 week OR IV Rx required

Oral Discomfort/ Mild discomfort, Difficulty swallowing Unable to swallow solids Unable to drink fluids;

Dysphagia no difficulty but able to eat and IV fluids req

swallowing drink

Constipation Mild Moderate Severe Distention with

vomiting

RESPIRATORY

Cough (for Transient; no Rx Treatment associated Uncontrolled cough; ________

aerosol studies) cough; inhaled systemic Rx req

bronchodilator

Bronchospasm Acute Transient; no Rx; Rx req; normalizes No normalization Cyanosis; FEV1 <25%

FEV1 <80% - 70% with bronchodilator; with bronchodilator; (or peak flow) OR

(or peak flow) FEV1 50%-<70% (or FEV1 25% - <50% (or intubated

peak flow) peak flow), retractions

Dyspnea Dyspnea on Dyspnea with normal Dyspnea at rest Dyspnea requiring

exertion activity O_2_ therapy

**PARAMETER GRADE 1 GRADE 2 GRADE 3 GRADE 4**

MILD MODERATE SEVERE POTENTIALLY LIFE THREATENING

NEUROLOGIC

Neuro-cerebellar Slight incoordination Intention tremor OR Ataxia requiring Unable to stand

OR dysdiadochokinesia dysmetria OR slurred assistance to walk or

speech OR nystagmus arm incoordination

interfering with ADLs

Neuro-psych/mood ________ _________ Severe mood changes Acute psychosis req

requiring medical hospitalization

intervention

Paresthesia Mild discomfort; no Mod discomfort; Severe discomfort; OR Incapacitating; OR

(burning, Rx req non-narcotic narcotic analgesia req not responsive to

tingling, etc) analgesia req with symptomatic narcotic analgesia

improvement

Neuro-motor Mild weakness in Mod weakness in feet Marked distal weakness Confined to bed or

muscle of feet but (unable to walk on (unable to dorsiflex wheel chair because of

able to walk and/or heels and/or toes), toes or foot drop), and muscle weakness

mild increase or mild weakness in mod proximal weakness

decrease in reflexes hands, still able to e.g., in hands interfering

do most hand tasks with ADLs and/or requiring

and/or loss of assistance to walk and/or

previously present unable to rise from chair

reflex or development unassisted

of hyperreflexia and/or

unable to do deep knee

bends due to weakness

Neuro-sensory Mild impairment (dec Mod impairment (mod Severe impairment Sensory loss involves

sensation, e.g., dec sensation, e.g., (dec or loss of limbs and trunk.

vibratory, pinprick, vibratory, pinprick, sensation to knees or

hot/cold in great toes) hot/cold to ankles) wrists) or loss of

in focal area or and/or joint position sensation of at least

symmetrical distri- or mild impairment mod degree in multiple

bution that is not different body areas

symmetrical (i.e., upper and lower

extremities)

**PARAMETER GRADE 1 GRADE 2 GRADE 3 GRADE 4**

MILD MODERATE SEVERE POTENTIALLY LIFE THREATENING

URINALYSIS

Proteinuria

Spot urine 1+ 2 - 3+ 4+ Nephrotic syndrome

24 hour urine 200 mg-1 g loss/day OR 1 - 2 g loss/day OR 2 - 3.5 g loss/day OR Nephrotic syndrome OR

<0.3% OR 0.3 - 1.0% OR >1.0% OR >3.5 g loss/day

<3 g/l 3 - 10 g/l >10 g/l

Gross Hematuria Microscopic only Gross, no clots Gross plus clots Obstructive OR

transfusion req

MISCELLANEOUS

Fever 37.7 - 38.5C OR 38.6 - 39.5C OR 39.6 - 40.5C OR >40.5C OR

oral >12 hours 100.0 - 101.5F 101.6 - 102.9F 103 - 105F >105F

Headache Mild; no Rx req Mod; or non-narcotic Severe; OR responds to Intractable; OR requiring

analgesia Rx initial narcotic Rx repeated narcotic Rx

Allergic Reaction Pruritus without Localized urticaria Generalized urticaria Anaphylaxis

rash angioedema

Cutaneous/Rash/ Erythema, pruritus Diffuse maculopapular Vesiculation OR moist ANY ONE: mucous membrane

Dermatitis rash OR dry desquamation OR involvement, suspected

desquamation ulceration Stevens-Johnson (TEN),

erythema multiforme,

necrosis req surgery,

exfoliative dermatitis

Local Reaction Erythema Induration <10mm OR Induration >10mm OR Necrosis of skin

(2^o^ parenteral Rx - inflammation OR ulceration

not vaccination or phlebitis

skin test

Fatigue Normal activity Normal activity Normal activity reduced Unable to care for

reduced <25% reduced 25-50% >

**Appendix 4 :**

**DIVISION OF AIDS (DAIDS)**

**TOXICITY TABLE FOR GRADING SEVERITY OF**

**PEDIATRIC (≤ 3 MONTHS OF AGE) ADVERSE EXPERIENCES**

For other findings, the toxicity table for children > 3 months of age (September 1993) is applicable.

All values here are for term newborns.

Preterm infants should be judged by a comparison of local normal ranges and the newborn ranges identified here.

| PARAMETER | GRADE 1 | GRADE 2 | GRADE 3 | GRADE 4 |
| --- | --- | --- | --- | --- |
| HEMATOLOGY |  |  |  |  |
| Hemoglobin |  |  |  |  |
| 1-7 days old | 13.0-14.0 | 12.0-12.9 | <12 | Cardiac Failure  2ndary to Anemia |
| 8-21 days old | 12.0-13.0 | 10.0-11.9 | <10.0 | Cardiac Failure  2ndary to Anemia |
| 22-35 days old | 9.5-10.5 | 8.0-9.4 | <8.0 | Cardiac Failure  2ndary to Anemia |
| 36-56 days old | 8.5-9.4 | 7.0-8.4 | <7.0 | Cardiac Failure  2ndary to Anemia |
| 57-90 days old | 9.0-9.9 | 7.0-8.9 | <7.0 | Cardiac Failure  2ndary to Anemia |
| Abs Neutrophil Ct | | | | |
| 1 day old | 5000-7000 | 3000-4999 | 1500-2999 | <1500 |
| 2-7 days old | 1750-2500 | 1250-1749 | 750-1249 | <750 |
| 8-56 days old | 1200-1800 | 900-1199 | 500-899 | <500 |
| 57-90 days old | 750-1200 | 400-749 | 250-399 | <250 |
| Bilirubin | | | | |
| <7 days old |  | 20-25 | 26-30 | >30 |
| 7-60 days old | 1.1-1.9xN | 2.0-2.9xN | 3.0-7.5xN | >7.5xN |
| 61-90 days old | 1.1-1.9xN | 2.0-2.9xN | 3.0-7.5xN | >7.5xN |
| Creatinine | | | | |
| <7 days old | 1.0-1.7 | 1.8-2.4 | 2.5-3.0 | >3.0 |
| 7-60 days old | 0.5-0.9 | 1.0-1.4 | 1.5-2.0 | >2.0 |
| 61-90 days old | 0.6-0.8 | 0.9-1.1 | 1.2-1.5 | >1.5 |
| Cr Clearance | | | | |
| <7 days old | 35-40 | 30-34 | 25-29 | <25 |
| 7-60 days old | 45-50 | 40-44 | 35-39 | <35 |
| 61-90 days old | 60-75 | 50-59 | 35-49 | <35 |
|  |  |  |  |  |
| Low Calcium | | | | |
| <7 days old | 6.5-6.9 | 6.0-6.4 | 5.5-5.9 | <5.5 |
| 7-60 days old | 7.6-8.0 | 7.0-7.5 | 6.0-6.9 | <6.0 |
| 61-90 days old | 7.8-8.4 | 7.0-7.7 | 6.0-6.9 | <6.0 |
| High Calcium | | | | |
| <7 days old | 12.0-12.4 | 12.5-12.9 | 13.0-13.5 | >13.5 |
| 7-60 days old | 10.5-11.2 | 11.3-11.9 | 12.0-13.0 | >13.0 |
| 61-90 days old | 10.5-11.2 | 11.3-11.9 | 12.0-12.9 | >= 13.0 |

TOXICITY TABLE for GRADING SEVERITY of PEDIATRIC (> 3 MONTHS OF AGE) ADVERSE EXPERIENCES

April, 1994 (Division of AIDS)

| PARAMETER | GRADE 1 | GRADE 2 | GRADE 3 | GRADE 4 |
| --- | --- | --- | --- | --- |
| HAEMATOLOGY |  |  |  |  |
| Haemoglobin  > 3 mo.- < 2 y.o. | 9.0-9.9 | 7.0-8.9 | <7.0 | Cardiac Failure  2ndary to anaemia |
| Hemoglobin > = 2 y.o. | 10-10.9 | 7.0-9.9 | <7.0 | Cardiac Failure  2ndary to anaemia |
| Abs Neutrophil Ct | 750-1200 | 400-749 | 250-399 | <250 |
| Platelets |  | 50,000-75,000 | 25,000-49,999 | <25,000 or bleeding |
| PT | 1.1-1.25xN | 1.26-1.5xN | 1.51-3.0xN | >3xN |
| PTT | 1.1-1.66xN | 1.67-2.33xN | 2.34-3.0xN | >3xN |
| GASTROINTESTINAL |  |  |  |  |
| Bilirubin | 1.1-1.9xN | 2.0-2.9xN | 3.0-7.5xN | >7.5xN |
| AST (SGOT) | 1.1-4.9xN | 5.0-9.9xN | 10.0-15.0xN | >15.0xN |
| ALT (SGPT) | 1.1-4.9xN | 5.0-9.9xN | 10.0-15.0xN | >15.0xN |
| GGT | 1.1-4.9xN | 5.0-9.9xN | 10.0-15.0xN | >15.0xN |
| Pancreatic Amylase | 1.1-1.4xN | 1.5-1.9xN | 2.0-3.0xN | >3.0xN |
| Total Amylase +  Lipase* | 1.1-1.4xN | 1.5-2.4xN | 2.5-5.0xN | >5.0xN |
| Uric Acid | 7.5-9.9 | 10-12.4 | 12.5-15.0 | >15.0 or Gout |
| CPK | See Neuromuscular Toxicity | | | |
| Abdominal Pain | Mild | Moderate-  No Rx Needed | Moderate-  Rx Needed | Severe-  Hospital and Rx |
| Diarrhoea | Soft stools | Liquid stools | Liquid Stools & Mild Dehydration Bloody stools | Dehydration requiring IV therapy or Hypotensive Shock |
| Constipation | Mild | Moderate | Severe | Distention and Vomiting |
| Nausea | Mild | Moderate-  Decreased po intake | Severe-Little  po intake | Unable to ingest food  or fluid for >24 hours |
| Vomiting | <1 episode/day | 1-3 episodes/day or duration >3d | >3 episodes/day or duration >7d | Intractable Vomiting |

Comments:

^*^Both amylase and lipase must be elevated to the same grade or higher (i.e. if total amylase is Grade 4, but lipase is only Grade 1, the Toxicity Grade is 1.

In paediatric HIV patients, the most common source of serum amylase is the salivary glands. Salivary amylase elevations are generally not clinically significant. When amylase is released from damaged pancreatic cells, it can be a marker of pancreatitis. In most cases of clinical pancreatitis, lipase will also be elevated. However, lipase is also a non-specific marker. Combined elevation of amylase and lipase (each >5 x normal) often indicateancreatic disease and requires evaluation. However, in the absence of pancreatic disease, drug can be resumed even at Grade 3 and 4 toxicities.

| PARAMETER | GRADE 1 | GRADE 2 | GRADE 3 | GRADE 4 |
| --- | --- | --- | --- | --- |
| RENAL AND ELECTROLYTES | | | | |
| CREATININE | | | | |
| 2 Month-2 Years | 0.6-0.8 | 0.9-1.1 | 1.2-1.5 | >1.5 |
| 2 Years-Adolescent | 0.7-1.0 | 1.1-1.6 | 1.7-2.0 | >2.0 |
| Adolescents | 1.0-1.7 | 1.8-2.4 | 2.5-3.5 | >3.5 |
| Creatinine Clearance | 60-75  cc/min/1.73 m^2^ | 50-59  cc/min/1.73 m^2^ | 35-49  cc/min/1.73 m^2^ | <35  cc/min/1.73 m^2^ |
| ELECTROLYTES |  |  |  |  |
| High Sodium | 145-149 |  | 150-155 | >155 or mental status changes |
| Low Sodium | 130-135 |  | 129-124 | <124 or mental status changes |
| High Potassium | 5.0-5.9 | 6.0-6.4 | 6.5-7.0 | >7.0 or Cardiac arrhythmias |
| Low Potassium | 3.0-3.5 | 2.5-2.9 | 2.0-2.4 | <2.0 |
| High Calcium | 10.5-11.2 | 11.3-11.9 | 12.0-12.9 | >=13.0 |
| Low Calcium | 7.8-8.4 | 7.0-7.7 | 6.0-6.9 | <6.0 |
| Low Magnesium | 1.2-1.4 | 0.9-1.1 | 0.6-0.8 | <0.6 or Cardiac arrhythmias |
| Hypoglycaemia | 55-65 | 40-54 | 30-39 | <30 or Mental status changes |
| Hyperglycaemia | 116-159 | 160-249 | 250-400 | >400 or Ketoacidosis |
| Proteinuria | Tr-1+  <150 mg/day | 2+  150-499 mg/day | 3+  500-1000 mg/day | 4+, or nephrotic syndrome  >1000 mg/day |
| Haematuria | Microscopic  <25 cells/hpf | Microscopic  >=25 cells/hpf | Gross | Obstruction or Transfusion requirement |
| Comments  Calcium values are corrected for albumin concentration. CrCl values do not apply to infants <2 months old. | | | | |
| OTHER | | | | |
| Allergy | Pruritis without Rash | Pruritic Rash | Mild Urticaria | Severe Urticaria  Anaphylaxis, Angioedema |
| Drug Fever (Rectal) |  | 38.5-40 | >40 | Sustained Fever:  >40, >5 days |
| Cutaneous |  | Diffuse maculo-  papular rash, dry desquamation | Vesiculation, ulcers | Exfoliative dermatitis, Stevens-Johnson or Erythema multiforme, Moist desquamation |
| Stomatitis | Mild discomfort | Painful, difficulty swallowing, but able to eat and drink | Painful: unable  to swallow solids | Painful: requires IV fluids |

| SYMPTOM | GRADE 1 | GRADE 2 | GRADE 3 | GRADE 4 |
| --- | --- | --- | --- | --- |
| CENTRAL NERVOUS SYSTEM | | | | |
| Seizures | None | 1 Uncomplicated Sz  +/- Temp Elevation | 1 Sz/Month for  >=2 Consecutive Months Or 3 Sz over 6 Months; No Temp Elevation | >1 Sz/Month;  No Temp Elevation;  No Decrease in Sz Frequency Despite dose reduction |
| Seizures are a ubiquitous symptom of numerous systemic or CNS disturbances; alternative explanations should be vigorously sought and eliminated. Status epilepticus represents a severe end of the seizure spectrum, but should be considered as a single seizure event. The need for chronic or acute anticonvulsant medication should be made on a clinical basis. Seizures as a manifestation of drug toxicity are usually primarily generalised. Focal (partial onset) seizures are suggestive of focal central nervous system pathology and should be appropriately investigated, although they may be a manifestation of drug toxicity. Beware of focal seizures which secondarily generalise; these should be approached diagnostically as partial onset seizures. Children with underlying epileptic conditions who experience persistent breakthrough seizures despite maximal anticonvulsant therapy coincident with beginning the trial medication should be considered Grade 4. | | | | |
| Headache | <=1/Month  <2 Hrs duration  Mild | >1/Month  >2 Hrs Duration  Moderate to Severe  Responds to non-narcotic  analgesia or prophylaxis | >2/Month  >2 Hrs Duration  Moderate to Severe  Responds to narcotic analgesia, or  does not respond  to prophylaxis | >4/Month;  >2 Hrs Duration;  Moderate to Severe;  Non-Responsive to narcotic  Analgesia; or persistently  Recurrent despite prophylaxis  No decrease in frequency or  Severity despite dose reduction |
| Headache is a non-specific symptom, but may be a symptom of CNS/intracranial pathology. Appropriate diagnostic measures should be pursued. Duration refers to the waxing and peak phases, not to the resolution/waning phases of the headache. Mild refers to a grade of headache pain which does not affect function or activity. Moderate to severe refers to a grade of headache which affects function or activity. | | | | |
| Mental Status  And Behaviour | Changes which do not Affect Function | Changes requiring pharmacologic or other therapy; or mild lethargy, sedation or somnolence which resolves with rest | Changes not improved by drugs or other therapies; or onset of confusion, memory impairment, lethargy, sedation, or somnolence which does not respond to rest | Onset of delirium, obtundation, coma, or psychosis, or Grade 3 toxicity which does not respond to dose reduction |
| Behaviour refers to the development of attention deficits with or without hyperactivity, depression, mania, agitation, sleep disorders, phobias, obsessive-compulsive behaviours, or anxiety. Mental status refers to the level of consciousness, memory function, language and analytical operations, and non-dominant hemisphere functioning. Alternative explanations should be sought. | | | | |
| Balance & Posture | None | None | Ataxia, dizziness, vertigo, tremor, impaired postural balance | Onset of movement disorder; or Grade 3 toxicity which does not respond to dosage adjustment |
| "Ataxia" can be mistakenly diagnosed in the face of central weakness or peripheral neuropathy, which should not be considered a drug toxicity of this category. Movement disorders refer to tardive or other dyskinesias, dystonias, chorea, or ballismus. Alternative explanations should be sought. | | | | |

| SYMPTOM | GRADE 1 | GRADE 2 | GRADE 3 | GRADE 4 |
| --- | --- | --- | --- | --- |
| Visual | None | Blurriness, diplopia, or horizontal nystagmus of < 1 hour duration, with spontaneous resolution | > = 1 episode of Grade 2 symptoms per week, or an episode of Grade 2 Sx lasting 1 hour with spontaneous resolution by 4 hours or vertical nystagmus | Decrease in visual acuity, visual field deficit, or oculogyric crisis, or Grade 3 Sx which persist after dose reduction |
| Many of the symptoms in this category can be the result of CNS pathology, or alternatively can be an external (i.e., non-CNS) neuro-ophthalmologic disorder. Appropriate diagnostic investigations should be pursued. | | | | |
| Myelopathy | None | None | None | Myelopathic/spinal cord symptoms, such as: Pyramidal tract weakness and disinhibition, sensory level, loss of proprioception, bladder/bowel dysfunction |
| HIV can cause spinal cord syndromes rarely in children. Other infectious agents can cause myelopathies as well. Alternative explanations should be sought. | | | | |
| PERIPHERAL NERVOUS SYSTEM | | | | |
| Neuropathy/  Lower Motor Neuronopathy | None | Mild transient  Paresthesia only | Persistent or progressive paresthesias, burning sensation in feet, or mild dysesthesia; no weakness; mild to moderate deep tendon reflex changes; no sensory loss | Onset of significant weakness, decrease or loss of DTRs, sensory loss in "stocking glove" distribution, radicular sensory loss, multiple cranial nerve involvement; bladder or bowel dysfunction, fasciculations, respiratory embarrassment from chest wall weakness. Grade 3 symptoms which do not resolve with dose reduction |
| Infectious agents other than HIV can precipitate a neuropathy and should be considered, especially CMV. Neuropathies which do not resolve after dose reduction or discontinuation should be pursued for alternative infectious or non-infectious etiologies, since drug-related neuropathies will usually resolve after dose reduction or drug discontinuation. It should be borne in mind that many patients will worsen for up to one month after drug discontinuation prior to improvement ("coasting"). Abnormalities should be confirmed by nerve conduction studies (NCS) +/- electromyographic studies (EMG). | | | | |
| Myopathy or Neuromuscular Junction Impairment | Normal or mild (<2 x N) CPK elevation | Mild proximal weakness and/or atrophy not affecting gross motor function. Mild myalgias, +/- mild CPK elevation (<2 x N) | Proximal muscle weakness and/or atrophy affecting motor function +/- CPK elevation; or severe myalgias with CPK >2 x N; Consider confirmatory EMG and/or muscle bx | Onset of myasthenia-like symptoms (fatiguable weakness with external, variable ophthalmoplegia and/or ptosis), or neuromuscular junction blockade (acute paralysis) symptoms (confirm with EMG); or Grade 3 symptoms which do not resolve on dose adjustment; confirm with muscle bx |
| HIV can produce a myopathy, and should be differentiated. Drug-induced myopathy can be accompanied by normal CPK levels. On occasion, neuropathic or central weakness can mimic myopathic weakness. | | | | |

| SYMPTOM | GRADE 1 | GRADE 2 | GRADE 3 | GRADE 4 |
| --- | --- | --- | --- | --- |
| Clinical symptoms *not otherwise specified* in this table | No therapy; monitor condition | May require minimal intervention and monitoring | Requires medical care and possible hospitalisation | Requires active medical intervention, hospitalisation, or hospice care |
| Laboratory values *not otherwise specified* in this table | Abnormal, but requiring no immediate intervention; follow | Sufficiently abnormal to require evaluation as to causality and perhaps mild therapeutic intervention, but not of sufficient severity to warrant immediate changes in study drug | Sufficiently severe to require evaluation and treatment, including at least temporary suspension of study drug | Life-threatening severity. Requires immediate evaluation, treatment, and usually hospitalisation. Study drug must be stopped immediately and should not be restarted until the abnormality is clearly felt to be caused by some other mechanism that study drug. |
