# Supplementary material for: Efficacy of Short-Course AZT Plus 3TC to Reduce Nevirapine Resistance in the Prevention of Mother-to-Child HIV Transmission: A Randomized Clinical Trial
Source: PLoS Med. 2009 Oct 27;6(10):e1000172. doi: 10.1371/journal.pmed.1000172 (PMC2760761; doi:10.1371/journal.pmed.1000172)
Supplement: Text S2 — Protocol amendment 1. (0.20 MB DOC) [file pmed.1000172.s002.doc]

Boehringer Ingelheim (Pty), Ltd.

ABCD

Boehringer Ingelheim (Pty), Ltd

404 Main Ave, Ferndale, Randburg, South Africa

**Clinical Trial Protocol** **Amendment**

| **Amendment Number:**  **Date:** | | | | 1 | | |  | | | |
| --- | --- | --- | --- | --- | --- | --- | --- | --- | --- | --- |
|  |  |  |  | 10 September 2002 | | |  | | |  |
| Trial No.: | | | 1100.1413 | | | ⌧ | | | Implemented only after documented approval of IRB / IEC | |
| Test Substance(s) | | | Nevirapine | | |  | | | Implemented immediately in order to eliminate immediate hazard IRB / IEC to be notified of change with request for approval | |
|  | | |  | | |  | | | Implemented immediately as changes involve only logistical or administrative aspects. IRB / IEC notified of changes only | |
| Title: | | | An Open-label Study evaluating the Resistance profile of Single dose Nevirapine(NVP) when combined with a 4 or 7 day course of Combivir® (ZDV/3TC) compared to Single dose Nevirapine for the Prevention of Mother to Child Transmission(pMTCT) of HIV - Treatment Options Preservation Study (T.O.P.S.) | | | | | | | |
| Changes: | | | Please see attached pages | | | | | | | |
| Reason For Change: | | 1. To extend the follow up period to 18 months for any patient who demonstrates genotypic resistance to nevirapine at 12 months. 2. To remove the HIV-1 resistance testing at the screening visit. 3. To add an additional exclusion criterion for the study. 4. To indicate the change from using a local laboratory to that of a central laboratory in South Africa 5. To correct all typographical errors. | | | | | | | | |
|  | | | | |  | | | **Page 1 of 11** | | |
| Confidential | © Boehringer Ingelheim  This protocol is the property of Boehringer Ingelheim and may not - in full or in part - be passed on, reproduced, published or otherwise used without the express permission of Boehringer Ingelheim | | | | | | | | | |

# PROTOCOL AMENDMENT SIGNATURE PAGE

| **BI Trial No.:** | | 1100.1413 | | | | |  | | | | | |
| --- | --- | --- | --- | --- | --- | --- | --- | --- | --- | --- | --- | --- |
| **Amendment No.:** | | 1 | | | | |  | | | | | |
| Trial Clinical Monitor: | | |  | |  | | |  | |  | |  |
| Name | | |  | | Date | | |  | | John Steytler | |  |
| Organisation/Department | | |  | |  | | |  | | Boehringer Ingelheim (Pty) Ltd / Medical Dept | |  |
| Trial Statistician: (indicate early information on signature, if applicable) | | |  | |  | | |  | |  | |  |
| Name | | |  | | Date | | |  | | David Hall | |  |
| Organisation/Department | | |  | |  | | |  | | Boehringer Ingelheim Pharmaceuticals, Inc./ Biometrics and Data Management | |  |
| Medical Director: | | |  | |  | | |  | |  | |  |
| Name | | |  | | Date | | |  | | Lynette Boshoff | |  |
| Organisation/Department | | |  | |  | | |  | | Boehringer Ingelheim (Pty) Ltd / Medical Dept | |  |
| Team Member Medicine: (indicate early information on approval, if applicable | | |  | |  | | |  | |  | |  |
| Name | | |  | | Date | | |  | | Patrick Robinson for M. Imperiale | |  |
| Organisation/Department | | |  | |  | | |  | | Boehringer Ingelheim Pharmaceuticals, Inc. / Clinical Research | |  |
|  | I hereby certify that I agree to adhere to the protocol and to all the documents referenced in the protocol. | | | | | | | | | | | |
|  | Investigator: | | |  | |  | | |  | |  | |
|  | Name | | |  | | Date | | |  | |  | |
|  | Organisation/Department | | |  | |  | | |  | |  | |

| **Page**  **(Section Number)** | **Changes** | **Reason for Change** |
| --- | --- | --- |
| TP 5 and 6 | **See attachment 1 for flow chart amendments** | Extension of follow up period to 18 months, if patients demonstrate resistance at 12 months. |
| TP 6  Footnote 5 | Visit 8 concludes mother and infant participation in the trial for those mothers and infants with demonstrated resistance  **Amended to:**  Visit 8 concludes mother and infant participation in the trial for those mothers and infants with demonstrated resistance after visit 5, but with no demonstrated resistance at visit 8 | Extension of follow up period to 18 months, if patients demonstrate resistance at 12 months. |
| TP 6  Footnote 8 | Visit 8 also to be completed, if possible, for all dropouts and withdrawals  **Amended to:**  Visit 8 also to be completed, if possible, for all dropouts and withdrawals, between visits 5 to 8 | Extension of follow up period to 18 months, if patients demonstrate resistance at 12 months. |
| TP 6  Footnotes | Added:  Footnote 10  Visit 9 will conclude participation in the trial for all mothers and infants with demonstrated resistance at visit 8 | Extension of follow up period to 18 months, if patients demonstrate resistance at 12 months. |
| TP 17  Section 3.1  No. of subjects planned | Patient participation will be for 6weeks unless resistance is demonstrated in which case patients will be followed up for 12 months. 5 study centres in South Africa are envisaged to provide a total of 300 patients.  **Amended to:**  Patient participation will be for 6 weeks unless resistance is demonstrated in which case patients will be followed up for 12 months. All patients demonstrating resistance after 12 months will be followed for a further 6 month period. Five study centres in South Africa are envisaged to provide a total of 300 patients. | Extension of follow up period to 18 months, if patients demonstrate resistance at 12 months. |
| TP 18  Exclusion criteria | **Added:**  Clinical suspicion of intra-uterine foetal death | The randomisation of a mother when the foetus has already died would be unethical. |

| **Page**  **(Section Number)** | **Changes** | **Reason for Change** |
| --- | --- | --- |
| TP 27  Section 5.2 Safety  Heading: Clinical laboratory testing.  Paragraph 2 | All laboratory tests will be performed at a local laboratory, the requirements for the handling of blood specimens will be contained in the laboratory manual provided to all the investigators participating in the study.  **Amended to:**  All laboratory tests will be performed at a central laboratory located in South Africa, the requirements for the handling of blood specimens will be contained in the laboratory manual provided to all the investigators participating in the study. | A central laboratory with the necessary capacity to perform all the required tests will be used. |
| TP 30  Section 6.2  Study procedures at each visit.  Visit 1, mothers | Collect and submit blood sample for resistance and other HIV-1 virologic testing once HIV result confirmed as being positive.  **Deleted:**  ~~Collect and submit blood sample for resistance and other HIV-1 virologic testing once HIV result confirmed as being positive.~~ | Resistance testing removed at screening visit. |
| TP 32  Section 6.2  Study procedures at each visit.  Visit 8, mothers | Visit 8: (final visit day 336: ± 14 days)  **Amended to:**  Visit 8: (day 336: ± 14 days) | Extension of follow up period to 18 months, if patients demonstrate resistance at 12 months. Visit 8 may no longer be the final visit. |
| TP 33  Section 6.2  Study procedures at each visit.  Visit 8, mothers | This visit concludes participation in the study by the mother  **Amended to:**  This visit concludes participation in the study by the mother if there is no demonstrated genotypic resistance at this visit | Extension of follow up period to 18 months, if patients demonstrate resistance at 12 months. Visit 8 may no longer be the final visit. |
| TP 33  Section 6.2  Study procedures at each visit, mothers | **Added**:  Visit 9: (final visit, day 504 ± 14 days)  Assess mother for any HIV related signs and symptoms  Perform a targeted physical examination  Record any adverse events and concomitant therapies  Perform laboratory tests for CD4 , viral load , resistance and other HIV-1 virologic testing  This visit concludes participation in the study by the mother | Extension of follow up period to 18 months, if patients demonstrate resistance at 12 months. |

| **Page**  **(Section Number)** | **Changes** | **Reason for Change** |
| --- | --- | --- |
| TP 36  Section 6.2  Study procedures at each visit, infants. | Visit 8 : ( final visit day 336) ± 14 days  **Amended to:**  Visit 8: (day 336: ± 14 days) | Extension of follow up period to 18 months, if patients demonstrate resistance at 12 months. Visit 8 may no longer be the final visit. |
| TP 36  Section 6.2  Study procedures at each visit, infants. | This visit concludes participation in the study by the infant  **Amended to:**  This visit concludes participation in the study by the infant if there is no demonstrated genotypic resistance at this visit | Extension of follow up period to 18 months, if patients demonstrate resistance at 12 months. Visit 8 may no longer be the final visit. |
| TP 36  Section 6.2  Study procedures at each visit, infants | **Added**:  Visit 9 ( final visit, day 504 ± 14 days)  Perform a targeted physical examination on the infant  Assess the infant for HIV related signs and symptoms  Record any adverse events and all concomitant therapies in the infant  Record the method of infant feeding  Collect blood samples for viral load measurement (HIV RNA PCR) and resistance and other HIV-1 virologic testing  This visit concludes participation in the study by the infant | Extension of follow up period to 18 months, if patients demonstrate resistance at 12 months |
| TP 36  Section 6.3.1 | For reasons of flexibility due to unforeseen circumstances a period of 2 days for visit 4, 6 days for visit 5 and 5.1, 10 days for visits 6 and 7 and 14 days for visit 8, will be allowed as the time frame for rescheduling visits.  **Amended to:**  For reasons of flexibility due to unforeseen circumstances a period of 2 days for visit 4, 6 days for visit 5 and 5.1, 10 days for visits 6 and 7 and 14 days for all subsequent visits, will be allowed as the time frame for rescheduling visits |  |

**ATTACHMENT 1: FLOW CHART AND FOOTNOTE CHANGES**

**FLOW CHART:**

MOTHER

| Visit Number | 1 | 2 | 3 | 4 | **5^2, 3^** | **6**** | **7**** | **8^5, 8^**** |  |
| --- | --- | --- | --- | --- | --- | --- | --- | --- | --- |
|  | Screening  (Prenatal) | Enrolment  (Labour and delivery) |  |  |  |  |  | Final Visit | End of trial |
| Day | -14 to -1 | 0 | 1-2 | 14 (± 2) | 42 (± 6) | 90  ± 10) | 168  (± 10) | 336  (± 14) |  |
| Informed Consent | X |  |  |  |  |  |  |  |  |
| HIV ELISA Test | X^9^ |  |  |  |  |  |  |  |  |
| Demographics | X |  |  |  |  |  |  |  |  |
| Review Inclusion/Exclusion criteria | X | X |  |  |  |  |  |  |  |
| Medical History | X |  |  |  |  |  |  |  |  |
| Randomisation |  | X |  |  |  |  |  |  |  |
| Post delivery history |  |  | X |  |  |  |  |  |  |
| HIV related symptoms and signs | X |  | X |  | X | X | X | X |  |
| Physical Examination | X |  | X |  | X | X | X | X |  |
| Viral load PCR (RNA) | X | X | X | X | X | X | X | X |  |
| CD4 | X | X | X | X | X | X | X | X |  |
| Laboratory Tests ^4^ | X |  | X | X | X |  |  |  |  |
| Labour and Delivery History |  | X |  |  |  |  |  |  |  |
| Adverse Events | X | X | X | X | X | X | X | X |  |
| Concomitant Therapy | X | X | X | X | X | X | X | X |  |
| Drug Administration |  | X^1^ | X^1^ |  |  |  |  |  |  |
| Drug accountability/compliance |  | X | X | X |  |  |  |  |  |
| Sampling for Resistance testing | X | X | X | X | X | X | X | X |  |
| End of trial admin/trial completion |  |  |  |  |  |  |  |  | X^2,3,5^ |

**Amended to:**

MOTHER

| Visit Number | 1 | 2 | 3 | 4 | **5^2, 3^** | **6**** | **7**** | **8^5, 8^**** | 9*** |  |
| --- | --- | --- | --- | --- | --- | --- | --- | --- | --- | --- |
|  | Screening  (Prenatal) | Enrolment  (Labour and delivery) |  |  |  |  |  |  |  | End of trial |
| Day | -14 to -1 | 0 | 1-2 | 14  (± 2) | 42  (± 6) | 90  (± 10) | 168  (± 10) | 336  (± 14) | 504  (± 14) |  |
| Informed Consent | X |  |  |  |  |  |  |  |  |  |
| HIV ELISA Test | X^9^ |  |  |  |  |  |  |  |  |  |
| Demographics | X |  |  |  |  |  |  |  |  |  |
| Review Inclusion/Exclusion criteria | X | X |  |  |  |  |  |  |  |  |
| Medical History | X |  |  |  |  |  |  |  |  |  |
| Randomisation |  | X |  |  |  |  |  |  |  |  |
| Post delivery history |  |  | X |  |  |  |  |  |  |  |
| HIV related symptoms and signs | X |  | X |  | X | X | X | X | X |  |
| Physical Examination | X |  | X |  | X | X | X | X | X |  |
| Viral load PCR (RNA) | X | X | X | X | X | X | X | X | X |  |
| CD4 | X | X | X | X | X | X | X | X | X |  |
| Laboratory Tests ^4^ | X |  | X | X | X |  |  |  |  |  |
| Labour and Delivery History |  | X |  |  |  |  |  |  |  |  |
| Adverse Events | X | X | X | X | X | X | X | X | X |  |
| Concomitant Therapy | X | X | X | X | X | X | X | X | X |  |
| Drug Administration |  | X^1^ | X^1^ |  |  |  |  |  |  |  |
| Drug accountability/compliance |  | X | X | X |  |  |  |  |  |  |
| Sampling for Resistance testing | ~~X~~ | X | X | X | X | X | X | X | X |  |
| End of trial admin/trial completion |  |  |  |  |  |  |  |  |  | X^2,3,5,10^ |

INFANT

| **Visit Number** | **2** | **3** | **4** | **5^2 ,3^** | **5.1*** | **6**** | **7**** | **8^58^**** |  |
| --- | --- | --- | --- | --- | --- | --- | --- | --- | --- |
| **Day** | **0** | **1−2**  **(within 0-72 hrs)** | **14**  **(± 2)** | **42**  **(± 6)** | **49**  **(± 6)** | **90**  **(± 10)** | **168**  **(± 10)** | **336**  **(± 14)** | **End of trial** |
| Neonatal History (Including Apgar score). |  | X |  |  |  |  |  |  |  |
| Patient Demographics |  | X |  |  |  |  |  |  |  |
| Eligibility criteria |  | X |  |  |  |  |  |  |  |
| Record infant feeding method |  | X | X | X |  | X | X | X |  |
| Physical Examination |  | X | X | X |  | X | X | X |  |
| HIV related symptoms and signs |  |  |  | X |  | X |  | X |  |
| Laboratory Tests^4^ |  | X | X | X |  |  |  |  |  |
| PCR (RNA) |  | X | X | X | X | X | X | X |  |
| PCR (DNA) |  | X^7^ | X | X² | X |  |  |  |  |
| Drug Administration |  | X^1^, ^6^ |  |  |  |  |  |  |  |
| Drug Accountability/compliance |  | X | X |  |  |  |  |  |  |
| Adverse Events |  | X | X | X |  | X | X | X |  |
| Concomitant Therapy |  | X | X | X |  | X | X | X |  |
| Sampling for Resistance testing |  | X | X | X | X | X | X | X |  |
| End of trial admin/trial completion |  |  |  |  |  |  |  |  | X^2,3,5^ |

1. All mothers to receive a single dose of nevirapine in labour and will be randomised to either no Combivir® or 4 or 7 days of Combivir® , also to be administered while in labour. Infants to receive the same treatment as mother.
2. Visit 5 concludes patient participation if mother and infant do not demonstrate resistance or the infant remains HIV DNA PCR negative.
3. Visit 5 also to be completed ,if possible, for all dropouts and withdrawals prior to visit 5.
4. Laboratory tests refers to : Full blood count, serum creatinine, AST, ALT, ALP, Total bilirubin, amylase.
5. Visit 8 concludes mother and infant participation in the trial for those mothers and infants with demonstrated resistance.
6. Retrovir® and 3TC® administered to infant within 24 hours after birth , nevirapine , to be administered 24-72 hours after birth.
7. Initial HIV DNA PCR to be performed within 48 hours.
8. Visit 8 also to be completed, if possible, for all dropouts and withdrawals after visit 5.
9. Unless a positive HIV-1 ELISA test is documented.

* Extra visit for infants who test HIV DNA PCR positive for first time at visit 5.

****** **Only for those patients with resistant virus**

**Amended to:**

INFANT

| **Visit Number** | **2** | **** | **4** | **5^2 ,3^** | **5.1*** | **6**** | **7**** | **8^58^**** | **9***** |  |
| --- | --- | --- | --- | --- | --- | --- | --- | --- | --- | --- |
| **Day** | **0** | **2**  **(within 0-72 hrs)** | **14**  **(± 2)** | **42**  **(± 6)** | **49**  **(± 6)** | **90**  **(± 10)** | **168**  **(± 10)** | **336**  **(± 14)** | **504**  **(± 14)** | **End of Trial** |
| Neonatal History (Including Apgar score). |  | X |  |  |  |  |  |  |  |  |
| Patient Demographics |  | X |  |  |  |  |  |  |  |  |
| Eligibility criteria |  | X |  |  |  |  |  |  |  |  |
| Record infant feeding method |  | X | X | X |  | X | X | X | X |  |
| Physical Examination |  | X | X | X |  | X | X | X | X |  |
| HIV related symptoms and signs |  |  |  | X |  | X |  | X | X |  |
| Laboratory Tests^4^ |  | X | X | X |  |  |  |  |  |  |
| PCR (RNA) |  | X | X | X | X | X | X | X | X |  |
| PCR (DNA) |  | X^7^ | X | X² | X |  |  |  |  |  |
| Drug Administration |  | X^1^, ^6^ |  |  |  |  |  |  |  |  |
| Drug Accountability/compliance |  | X | X |  |  |  |  |  |  |  |
| Adverse Events |  | X | X | X |  | X | X | X | X |  |
| Concomitant Therapy |  | X | X | X |  | X | X | X | X |  |
| Sampling for Resistance testing |  | X | X | X | X | X | X | X | X |  |
| End of trial admin/trial completion |  |  |  |  |  |  |  |  |  | X^2,3,.5,10^ |

1. All mothers to receive a single dose of nevirapine in labour and will be randomised to either no Combivir® or 4 or 7 days of Combivir® , also to be administered while in labour. Infants to receive the same treatment as mother.
2. Visit 5 concludes patient participation if mother and infant do not demonstrate resistance or the infant remains HIV DNA PCR negative.
3. Visit 5 also to be completed, if possible, for all dropouts and withdrawals prior to visit 5.
4. Laboratory tests refer to: Full blood count, serum creatinine, AST, ALT, ALP, Total bilirubin, amylase.
5. Visit 8 concludes mother and infant participation in the trial for those mothers and infants with demonstrated resistance after visit 5, but with no demonstrated resistance at visit 8.
6. Retrovir® and 3TC® administered to infant within 24 hours after birth, nevirapine to be administered 24-72 hours after birth.
7. Initial HIV DNA PCR to be performed within 48 hours.
8. Visit 8 also to be completed, if possible, for all dropouts and withdrawals between visits 5 to 8
9. Unless a positive HIV-1 ELISA test is documented.
10. Visit 9 will conclude participation in the trial for all mothers and infants with demonstrated resistance at visit 8.

* Extra visit for infants who test HIV DNA PCR positive for first time at visit 5

****** Only for those patients with resistant virus

******* Visit 9 is intended for those patients with demonstrated genotypic resistance at visit 8
