# Supplementary material for: Efficacy of Short-Course AZT Plus 3TC to Reduce Nevirapine Resistance in the Prevention of Mother-to-Child HIV Transmission: A Randomized Clinical Trial
Source: PLoS Med. 2009 Oct 27;6(10):e1000172. doi: 10.1371/journal.pmed.1000172 (PMC2760761; doi:10.1371/journal.pmed.1000172)
Supplement: Text S3 — Protocol amendment 2. (0.24 MB DOC) [file pmed.1000172.s003.doc]

Boehringer Ingelheim (Pty), Ltd.

ABCD

Boehringer Ingelheim (Pty), Ltd

404 Main Ave, Ferndale, Randburg, South Africa

**Clinical Trial Protocol** **Amendment**

| **Amendment Number:**  **Date:** | | | | 2 | | |  | | | |
| --- | --- | --- | --- | --- | --- | --- | --- | --- | --- | --- |
|  |  |  |  | 06 November 2002 | | |  | | |  |
| Trial No.: | | | 1100.1413 | | | ⌧ | | | Implemented only after documented approval of IRB / IEC | |
| Test Substance(s) | | | Nevirapine | | |  | | | Implemented immediately in order to eliminate immediate hazard IRB / IEC to be notified of change with request for approval | |
|  | | |  | | |  | | | Implemented immediately as changes involve only logistical or administrative aspects. IRB / IEC notified of changes only | |
| Title: | | | An Open-label Study evaluating the Resistance profile of Single dose Nevirapine (NVP) when combined with a 4 or 7 day course of Combivir® (ZDV/3TC) compared to Single dose Nevirapine for the Prevention of Mother to Child Transmission (pMTCT) of HIV - Treatment Options Preservation Study (T.O.P.S.) | | | | | | | |
| Changes: | | | Please see attached pages | | | | | | | |
| Reason For Change: | | 1. To allow infant dosing of nevirapine suspension 2mg/kg just prior to discharge, due to rapid patient turnover times in some hospitals. 2. To allow sufficient time for careful selection of patients presenting at antenatal clinics during the screening period. 3. To make a minor adjustment to 3 inclusion criteria and one exclusion criterion. 4. To repeat safety laboratory tests at baseline, visit 2. 5. Required blood tests during initial visits clarified. 6. To add an interim visit, 1.1, to allow for adequate review of laboratory results in order to determine patient eligibility. 7. To add an interim visit, 3.1, 7 days after delivery, for drug accountability purposes.   6. To add an additional visit, 7.1, 9 months after delivery to facilitate cohort retention. | | | | | | | | |
|  | | | | |  | | | **Page 1 of 14** | | |
| Confidential | © Boehringer Ingelheim  This protocol is the property of Boehringer Ingelheim and may not - in full or in part - be passed on, reproduced, published or otherwise used without the express permission of Boehringer Ingelheim | | | | | | | | | |

# PROTOCOL AMENDMENT SIGNATURE PAGE

| **BI Trial No.:** | 1100.1413 | | |  | | |
| --- | --- | --- | --- | --- | --- | --- |
| **Amendment No.:** | 2 | | |  | | |
| Trial Clinical Monitor: | |  |  | |  |  |
| Name  Organisation/Department | |  | date | |  | Dr. John Steytler  Boehringer Ingelheim (Pty) Ltd / Medical Dept. |
| Trial Statistician: (indicate early information on signature, if applicable) | |  |  | |  |  |
| Name  Organisation/Department | |  | date | |  | Dr. David Hall  Boehringer Ingelheim Pharmaceuticals, Inc./ Biometrics  and Data Management |
| Medical Director: | |  |  | |  |  |
| Name  Organisation/Department | |  | date | |  | Dr. Lynette Boshoff  Boehringer Ingelheim (Pty) Ltd / Medical Dept. |
| Team Member Medicine: (indicate early information on approval, if applicable) | |  |  | |  |  |
| Name  Organisation/Department | |  | date | |  | Dr. Patrick A. Robinson, for Dr. Michael Imperiale  Boehringer Ingelheim Pharmaceuticals, Inc. / Clinical Research |
| I herewith certify that I agree to adhere to the amended trial protocol and to all documents referenced in the amended trial protocol. | | | | | | |
| Investigator: | |  |  | |  |  |
| Name | |  | date | |  |  |
| Organisation/Department | |  |  | |  |  |

| **Page**  **(Section Number)** | **Changes** | **Reason for Change** |
| --- | --- | --- |
| TP 5  Flow chart changes | **See attachment # 1**  **Additional visits and footnotes 11 and 12 added.** | To allow sufficient time, for careful selection of patients presenting at antenatal clinics prior to presenting in labour, and for the receipt all laboratory test results. The inclusion of interim visits to facilitate better cohort retention. |
| TP 6  Footnote 6 | 6. Retrovir® and 3TC® administered to infant within 24 hours after birth, nevirapine to be administered 24-72 hours after birth.  **Amended to:**  6. Retrovir® and 3TC® administered to infant within 24 hours after birth, nevirapine to be administered 24-72 hours after birth or just prior to discharge if hospital stay is less than 24 hours. | To allow infant dosing of nevirapine suspension 2mg/kg just prior to discharge in cases where hospital stay is less than 24 hours, due to rapid patient turnover times in some academic hospitals. |
| TP 17  Section 3.2  Inclusion criteria | Pregnant women who present at antenatal clinics after 36 weeks gestation and are antiretroviral drug naïve.  **Amended to:**  Pregnant women identified at antenatal clinics from 34 weeks gestation and who are antiretroviral drug naïve. | To facilitate the recruitment process during the screening period. |
| TP 17  Section 3.2 Inclusion criteria | Mothers with a documented positive HIV ELISA test.  **Amended to:**  Mothers with a documented positive HIV Rapid test confirmed by a detectable HIV-1 RNA PCR (viral load). | Most antenatal clinics preferentially use the HIV Rapid test as apposed to the Elisa test |

| **Page**  **(Section Number)** | **Changes** | **Reason for Change** |
| --- | --- | --- |
| TP 17  Section 3.2 Inclusion criteria | Mothers to have a baseline viral load > 2000 RNA copies/mL.  **Amended to:**  Mothers to have a screening viral load > 2000 RNA copies/mL. | The decision to randomise would be determined by screening laboratory test results. |
| TP 18  Section 3.3  Exclusion criteria | Patients with evidence of hepatic dysfunction as measured by total bilirubin > 2.5 times ULN or AST/ALT > 5 times ULN.  **Amended to:**  Patients with evidence of hepatic dysfunction as measured by total bilirubin > 2.5 times ULN or AST/ALT > 5 times ULN at the screening visit. | The decision to randomise would be determined by screening laboratory test results. |
| TP 20 Section 4.1.3, Paragraph 3  AND  TP 29, Section 6.1 Paragraph 3 | Neonates will receive the same treatment as their mother, either:   1. A single dose of 2mg/kg nevirapine suspension within 24-72 hours after birth. 2. A single dose of nevirapine (2mg/kg) administered within 24-72 hours after birth plus Retrovir® oral suspension 12 mg bid and 3TC® oral suspension 6mg bid for 4 days commencing within 24 hrs after birth. 3. A single dose of nevirapine (2mg/kg) administered within 24-72 hours after birth plus Retrovir® oral suspension 12 mg bid and 3TC® oral suspension 6mg bid for 7 days commencing within 24 hrs after birth.   **Amended to:**  Neonates will receive the same treatment as their mother, either:  1. A single dose of 2mg/kg nevirapine suspension within 24- 72 hours after birth or just prior to discharge if hospital stay is less than 24 hours.   1. A single dose of nevirapine (2mg/kg) administered within 24-72 hours after birth, or just prior to discharge if hospital stay is less than 24 hours, plus Retrovir® oral suspension 12 mg bid and 3TC® oral suspension 6mg bid for 4 days commencing within 24 hrs after birth.   3. A single dose of nevirapine (2mg/kg) administered within 24-72 hours after birth, or just prior to discharge if hospital stay is less than 24 hours, plus Retrovir® oral suspension 12mg bid and 3TC® oral suspension 6mg bid for 7 days commencing within 24 hours after birth. | To allow infant dosing of nevirapine suspension 2mg/kg just prior to discharge in cases where hospital stay is less than 24 hours, due to rapid patient turnover times in some academic hospitals. |
| TP26  Section 5.2 Heading: Clinical laboratory testing  First paragraph | Clinical laboratory testing for mothers which will include serum creatinine, ALT, AST, ALP, Total Bilirubin, amylase, full blood count , CD4+ count, HIV RNA PCR (viral load), and HIV-1 resistance testing will be conducted on all patients at the screening visit (Visit 1) and repeated at all visits up to visit 5, excluding visit 2 for mothers (labour and enrollment), but including a withdrawal visit if this occurs during the initial 6 week follow up period.  **Amended to:**  Clinical laboratory testing for mothers which will include serum creatinine, ALT, AST, ALP, Total Bilirubin, amylase, full blood count, CD4+ count, HIV RNA PCR (viral load) will be conducted on all patients at the screening visit. At visit 2 blood will be collected for serum creatinine, ALT, AST, ALP, Total Bilirubin, amylase, full blood count, CD4+ count, HIV RNA PCR and HIV-1 resistance and other HIV-1 virologic testing.  At visits 3-5, and for any withdrawal visit during the initial 6 week follow up period, blood will be collected for serum creatinine, ALT, AST, ALP, Total Bilirubin, amylase, full blood count, CD4+ count, HIV RNA PCR (viral load) and HIV-1 resistance testing. | Resistance testing removed at screening visit with amendment 1, dated 10 September 2002. Required blood tests clarified for initial visits. Additional safety measure for mothers who may have had screening blood tests more than 4 weeks prior to randomisation visit. |

| TP 31 Section 6.2  Study procedures at each visit, mothers.  Visit 2: Day 0 (enrollment, labour and delivery) | **Added:**  Collect blood for the following laboratory tests: serum creatinine, ALT, AST, ALP, Total Bilirubin, amylase and full blood count. | Additional safety measure for mothers who may have been screened more than 4 weeks prior to randomisation visit. |
| --- | --- | --- |

| **Page**  **(Section Number)** | **Changes** | **Reason for Change** |
| --- | --- | --- |
| TP 30  Section 6.2  Study procedures at each visit, Mothers | Visit 1: Day –14 to -1 (prenatal screening)  **Amended to :**  Visit 1: ≥ Gestational age 34 weeks (prenatal screening) | To allow sufficient time, for careful selection of patients presenting at antenatal clinics. and for the receipt of virology test results which require a longer turnaround time than safety laboratory tests. |
| TP 30  Section 6.2  Study procedures at each visit, Mothers | **Added:**  Visit 1.1: > Gestational age 34 weeks to day  -0 (screening eligibility visit)   - Review inclusion/exclusion criteria. - Record any adverse events and all concomitant therapies. | To facilitate the eligibility evaluation. |
| TP 31  Section 6.2  Study procedures at each visit, Mothers | **Added:**  Visit 3.1: Day 8 ±1 (drug accountability visit)   - Perform drug accountability/compliance assessment. - Record any adverse events and all concomitant therapies. | This visit added from a safety and compliance point of view. |
| TP 32  Section 6.2  Study procedures at each visit, Mothers | **Added:**  Visit 7.1: Day 252 ±10 days   - Assess mother for any HIV related signs and symptoms. - Perform a targeted physical examination as before. - Record any adverse events and concomitant therapies. - Collect blood samples for CD4+, viral load measurement (HIV RNA PCR) and possible resistance and other HIV-1 virologic testing. | To facilitate better cohort retention. |

| **Page**  **(Section Number)** | **Changes** | **Reason for Change** |
| --- | --- | --- |
| TP 34  Section 6.2  Study procedures at each visit, Infants | **Added:**  Visit 3.1: Day 8 ±1 (drug accountability visit)   - Perform drug accountability/compliance assessment. - Record any adverse events and all concomitant therapies. | This visit added from a safety and compliance point of view. |
| TP 36  Section 6.2  Study procedures at each visit, Infants | **Added:**  Visit 7.1: Day 252 ±10 days   - Perform a targeted physical examination on the infant. - Record any adverse events and all concomitant therapies in the infant. - Record the method of infant feeding. - Collect blood samples for viral load measurement (HIV RNA PCR) and resistance and other HIV-1 virologic testing. | Additional visit added at 9 months post - delivery to facilitate better cohort retention. |

**Attachment 1:**

**Flow chart changes**

MOTHER

| Visit Number | 1 | 2 | 3 | 4 | **5^2, 3^** | **6**** | **7**** | **8^5, 8^**** | 9*** |  |
| --- | --- | --- | --- | --- | --- | --- | --- | --- | --- | --- |
|  | Screening  (Prenatal) | Enrollment  (Labour and delivery) |  |  |  |  |  |  |  | End of trial |
| Day | -14 to -1 | 0 | 1-2 | 14  (± 2) | 42  (± 6) | 90  (± 10) | 168  (± 10) | 336  (± 14) | 504  (± 14) |  |
| Informed Consent | X |  |  |  |  |  |  |  |  |  |
| HIV ELISA Test | X^9^ |  |  |  |  |  |  |  |  |  |
| Demographics | X |  |  |  |  |  |  |  |  |  |
| Review Inclusion/Exclusion criteria | X | X |  |  |  |  |  |  |  |  |
| Medical History | X |  |  |  |  |  |  |  |  |  |
| Randomisation |  | X |  |  |  |  |  |  |  |  |
| Post delivery history |  |  | X |  |  |  |  |  |  |  |
| HIV related symptoms and signs | X |  | X |  | X | X | X | X | X |  |
| Physical Examination | X |  | X |  | X | X | X | X | X |  |
| Viral load PCR (RNA) | X | X | X | X | X | X | X | X | X |  |
| CD4 | X | X | X | X | X | X | X | X | X |  |
| Laboratory Tests ^4^ | X |  | X | X | X |  |  |  |  |  |
| Labour and Delivery History |  | X |  |  |  |  |  |  |  |  |
| Adverse Events | X | X | X | X | X | X | X | X | X |  |
| Concomitant Therapy | X | X | X | X | X | X | X | X | X |  |
| Drug Administration |  | X^1^ | X^1^ |  |  |  |  |  |  |  |
| Drug accountability/ compliance |  | X | X | X |  |  |  |  |  |  |
| Sampling for Resistance testing |  | X | X | X | X | X | X | X | X |  |
| End of trial admin/trial completion |  |  |  |  |  |  |  |  |  | X^2,3,5,10^ |

**Amended to:**

MOTHER

| Visit Number | 1 | 1.1 | 2 | 3 | 3.1 ^12^ | 4 | **5^2, 3^** | **6**** | **7**** | **7.1** | **8^5, 8^**** | 9*** |  |
| --- | --- | --- | --- | --- | --- | --- | --- | --- | --- | --- | --- | --- | --- |
|  | Screening  (Prenatal) | Screening Eligi-  bility  Visit | Enrollment  (Labour and delivery) |  | Drug Account-ability Visit |  |  |  |  |  |  |  | End of trial |
| Day | ≥ 34 weeks gestation | >Gest. age 34 w to Day 0 | 0 | 1-2 | 8 (±1) | 14  (± 2) | 42  (± 6) | 90  (± 10) | 168  (± 10) | 252  (± 10) | 336  (± 14) | 504  (± 14) |  |
| Informed Consent | X |  |  |  |  |  |  |  |  |  |  |  |  |
| HIV ELISA Test | X^9^ |  |  |  |  |  |  |  |  |  |  |  |  |
| Demographics | X |  |  |  |  |  |  |  |  |  |  |  |  |
| Review Inclusion/ Exclusion criteria | X | X | X |  |  |  |  |  |  |  |  |  |  |
| Medical History | X |  |  |  |  |  |  |  |  |  |  |  |  |
| Randomisation |  |  | X |  |  |  |  |  |  |  |  |  |  |
| Post delivery history |  |  |  | X |  |  |  |  |  |  |  |  |  |
| HIV related symptoms and signs | X |  |  | X |  |  | X | X | X | X | X | X |  |
| Physical Examination | X |  |  | X |  |  | X | X | X | X | X | X |  |
| Viral load PCR (RNA) | X |  | X | X |  | X | X | X | X | X | X | X |  |
| CD4 | X |  | X | X |  | X | X | X | X | X | X | X |  |
| Laboratory Tests ^4^ | X |  | X | X |  | X | X |  |  |  |  |  |  |
| Labour and Delivery History |  |  | X |  |  |  |  |  |  |  |  |  |  |
| Adverse Events | X | X | X | X | X | X | X | X | X | X | X | X |  |
| Concomitant Therapy | X | X | X | X | X | X | X | X | X | X | X | X |  |
| Drug Administration |  |  | X^1^ | X^1^ |  |  |  |  |  |  |  |  |  |
| Drug accountability/ compliance |  |  | X | X | X | X |  |  |  |  |  |  |  |
| Sampling for Resistance testing |  |  | X | X |  | X | X | X | X | X^11^ | X | X |  |
| End of trial admin/trial completion |  |  |  |  |  |  |  |  |  |  |  |  | X^2,3,5,10^ |

INFANT

| **Visit Number** | **2** | **3** | **4** | **5^2 ,3^** | **5.1*** | **6**** | **7**** | **8^58^**** | **9***** |  |
| --- | --- | --- | --- | --- | --- | --- | --- | --- | --- | --- |
| **Day** | **0** | **1-2 −**  **(within 0-72 hrs)** | **14**  **(± 2)** | **42**  **(± 6)** | **49**  **(± 6)** | **90**  **(± 10)** | **168**  **(± 10)** | **336**  **(± 14)** | **504**  **(± 14)** | **End of Trial** |
| Neonatal History (Including Apgar score). |  | X |  |  |  |  |  |  |  |  |
| Patient Demographics |  | X |  |  |  |  |  |  |  |  |
| Eligibility criteria |  | X |  |  |  |  |  |  |  |  |
| Record infant feeding method |  | X | X | X |  | X | X | X | X |  |
| Physical Examination |  | X | X | X |  | X | X | X | X |  |
| HIV related symptoms and signs |  |  |  | X |  | X |  | X | X |  |
| Laboratory Tests^4^ |  | X | X | X |  |  |  |  |  |  |
| PCR (RNA) |  | X | X | X | X | X | X | X | X |  |
| PCR (DNA) |  | X^7^ | X | X² | X |  |  |  |  |  |
| Drug Administration |  | X^1^, ^6^ |  |  |  |  |  |  |  |  |
| Drug Accountability/ compliance |  | X | X |  |  |  |  |  |  |  |
| Adverse Events |  | X | X | X |  | X | X | X | X |  |
| Concomitant Therapy |  | X | X | X |  | X | X | X | X |  |
| Sampling for Resistance testing |  | X | X | X | X | X | X | X | X |  |
| End of trial admin/trial completion |  |  |  |  |  |  |  |  |  | X^2,3,.5,10^ |

1. All mothers to receive a single dose of nevirapine in labour and will be randomised to either no Combivir® or 4 or 7 days of Combivir®, also to be administered while in labour. Infants to receive the same treatment as mother.

1. Visit 5 concludes patient participation if mother and infant do not demonstrate resistance or the infant remains HIV DNA PCR negative.
2. Visit 5 also to be completed, if possible, for all dropouts and withdrawals prior to visit 5.
3. Laboratory tests refer to: Full blood count, serum creatinine, AST, ALT, ALP, Total bilirubin, amylase.
4. Visit 8 concludes mother and infant participation in the trial for those mothers and infants with demonstrated resistance after visit 5, but with no demonstrated resistance at visit 8.
5. Retrovir® and 3TC® administered to infant within 24 hours after birth, nevirapine to be administered 24-72 hours after birth.
6. Initial HIV DNA PCR to be performed within 48 hours.
7. Visit 8 also to be completed, if possible, for all dropouts and withdrawals between visits 5 to 8.
8. Unless a positive HIV-1 ELISA test is documented.
9. Visit 9 will conclude participation in the trial for all mothers and infants with demonstrated resistance at visit 8.

* Extra visit for infants who test HIV DNA PCR positive for first time at visit 5

****** Only for those patients with resistant virus

******* Visit 9 is intended for those patients with demonstrated genotypic resistance at visit 8

**Amended to:**

INFANT

| **Visit Number** | **2** | **3** | **3.1^12^** | **4** | **5^2 ,3^** | **5.1*** | **6**** | **7**** | **7.1** | **8^58^**** | **9***** |  |
| --- | --- | --- | --- | --- | --- | --- | --- | --- | --- | --- | --- | --- |
| **Day** | **0** | **1-2**  **(within 0-72 hrs)** | **8**  **± 1** | **14**  **(± 2)** | **42**  **(± 6)** | **49**  **(± 6)** | **90**  **(± 10)** | **168**  **(± 10)** | **252**  **(± 10)** | **336**  **(± 14)** | **504**  **(± 14)** | **End of Trial** |
| Neonatal History (Including Apgar score) |  | X |  |  |  |  |  |  |  |  |  |  |
| Patient Demographics |  | X |  |  |  |  |  |  |  |  |  |  |
| Eligibility criteria |  | X |  |  |  |  |  |  |  |  |  |  |
| Record infant feeding method |  | X |  | X | X |  | X | X | X | X | X |  |
| Physical Examination |  | X |  | X | X |  | X | X | X | X | X |  |
| HIV related symptoms and signs |  |  |  |  | X |  | X |  |  | X | X |  |
| Laboratory Tests^4^ |  | X |  | X | X |  |  |  |  |  |  |  |
| PCR (RNA) |  | X |  | X | X | X | X | X | X | X | X |  |
| PCR (DNA) |  | X^7^ |  | X | X² | X |  |  |  |  |  |  |
| Drug Administra-tion |  | X^1^, ^6^ |  |  |  |  |  |  |  |  |  |  |
| Drug Accountability/  compliance |  | X | X | X |  |  |  |  |  |  |  |  |
| Adverse Events |  | X | X | X | X |  | X | X | X | X | X |  |
| Concomitant Therapy |  | X | X | X | X |  | X | X | X | X | X |  |
| Sampling for Resistance testing |  | X |  | X | X | X | X | X | X^11^ | X | X |  |
| End of trial admin/trial completion |  |  |  |  |  |  |  |  |  |  |  | X^2,3,.5,10^ |

1. All mothers to receive a single dose of nevirapine in labour and will be randomised to either no Combivir® or 4 or 7 days of Combivir®, also to be administered while in labour. Infants to receive the same treatment as mother.

1. Visit 5 concludes patient participation if mother and infant do not demonstrate resistance or the infant remains HIV DNA PCR negative.
2. Visit 5 also to be completed, if possible, for all dropouts and withdrawals prior to visit 5.

4. Laboratory tests refer to: Full blood count, serum creatinine, AST, ALT, ALP, Total bilirubin, amylase.

5. Visit 8 concludes mother and infant participation in the trial for those mothers and infants with demonstrated resistance after visit 5, but with no demonstrated resistance at visit 8.

6. Retrovir® and 3TC® administered to infant within 24 hours after birth, nevirapine to be administered 24-72 hours after birth, or just prior to discharge if hospital stay is less than 24 hours.

7. Initial HIV DNA PCR to be performed within 48 hours.

8. Visit 8 also to be completed, if possible, for all dropouts and withdrawals between visits 5 to 8.

1. Unless a positive HIV-1 ELISA test is documented.
2. Visit 9 will conclude participation in the trial for all mothers and infants with demonstrated resistance at visit 8.
3. The sample will be stored from this visit, and will be tested for resistance only if the patient is lost to follow up after this visit, or if there was no detectable resistance at visit 8.
4. This visit is applicable only to those patients randomised to either the 4 or 7 day CBV arm.

* Extra visit for infants who test HIV DNA PCR positive for first time at visit 5.

****** Only for those patients with resistant virus.

******* Visit 9 is intended for those patients with demonstrated genotypic resistance at visit 8.
