# Supplementary material for: Efficacy of Short-Course AZT Plus 3TC to Reduce Nevirapine Resistance in the Prevention of Mother-to-Child HIV Transmission: A Randomized Clinical Trial
Source: PLoS Med. 2009 Oct 27;6(10):e1000172. doi: 10.1371/journal.pmed.1000172 (PMC2760761; doi:10.1371/journal.pmed.1000172)
Supplement: Text S4 — Protocol amendment 3. (0.25 MB DOC) [file pmed.1000172.s004.doc]

Boehringer Ingelheim (Pty), Ltd.

ABCD

Boehringer Ingelheim (Pty), Ltd

404 Main Ave, Ferndale, Randburg, South Africa

**Clinical Trial Protocol** **Amendment**

| **Amendment Number:**  **Date:** | | | | 3 | | |  | | | |
| --- | --- | --- | --- | --- | --- | --- | --- | --- | --- | --- |
|  |  |  |  | 17 April 2003 | | |  | | |  |
| Trial No.: | | | 1100.1413 | | | ⌧ | | | Implemented only after documented approval of IRB / IEC | |
| Test Substance(s) | | | Nevirapine | | |  | | | Implemented immediately in order to eliminate immediate hazard IRB / IEC to be notified of change with request for approval | |
|  | | |  | | |  | | | Implemented immediately as changes involve only logistical or administrative aspects. IRB / IEC notified of changes only | |
| Title: | | | An Open-label Study evaluating the Resistance profile of Single dose Nevirapine (NVP) when combined with a 4 or 7 day course of Combivir® (ZDV/3TC) compared to Single dose Nevirapine for the Prevention of Mother to Child Transmission (pMTCT) of HIV - Treatment Options Preservation Study (T.O.P.S.) | | | | | | | |
| Changes: | | | Please see attached pages | | | | | | | |
| Reason For Change: | | 1. To correct a typographical error on page 1 of amendment 2. 2. To amend the flow chart to include changes made to the inclusion criteria with amendment 2, dated 6 November 2002 and changes included in this amendment. 3. To clarify the treatment to be administered to mothers who are randomised in false labour. 4. To clarify the timeframe for infant dosing at sites with rapid patient turnaround times. 5. To allow greater flexibility for the scheduling of visit 3.1, the drug accountability visit. | | | | | | | | |
|  | | | | |  | | | **Page 1 of 4** | | |
| Confidential | © Boehringer Ingelheim  This protocol is the property of Boehringer Ingelheim and may not - in full or in part - be passed on, reproduced, published or otherwise used without the express permission of Boehringer Ingelheim | | | | | | | | | |

# PROTOCOL AMENDMENT SIGNATURE PAGE

| **BI Trial No.:** | 1100.1413 | | |  | | |
| --- | --- | --- | --- | --- | --- | --- |
| **Amendment No.:** | 3 | | |  | | |
| Trial Clinical Monitor: | |  |  | |  |  |
| Name  Organisation/Department | |  | date | |  | Dr. John Steytler  Boehringer Ingelheim (Pty) Ltd / Medical Dept. |
| Trial Statistician: (indicate early information on signature, if applicable) | |  |  | |  |  |
| Name  Organisation/Department | |  | date | |  | Mr. Toshio Kimura  Boehringer Ingelheim Pharmaceuticals, Inc./ Biometrics  and Data Management |
| Medical Director: | |  |  | |  |  |
| Name  Organisation/Department | |  | date | |  | Dr. Lynette Boshoff  Boehringer Ingelheim (Pty) Ltd / Medical Dept. |
| Team Member Medicine: (indicate early information on approval, if applicable) | |  |  | |  |  |
| Name  Organisation/Department | |  | date | |  | Dr. Michael Imperiale  Boehringer Ingelheim Pharmaceuticals, Inc. / Clinical Research |
| I herewith certify that I agree to adhere to the amended trial protocol and to all documents referenced in the amended trial protocol. | | | | | | |
| Investigator: | |  |  | |  |  |
| Name | |  | date | |  |  |
| Organisation/Department | |  |  | |  |  |

| **Page**  **(Section Number)** | **Changes** | **Reason for Change** |
| --- | --- | --- |
| Amendment # 2 AM 1,  Section: Reasons for change | 1. To add an additional visit, 7.1, 9 months after delivery to facilitate cohort retention.   **Amended to:**  8. To add an additional visit, 7.1, 9 months after delivery to facilitate cohort retention. | Incorrect numbering sequence. |
| Flow chart and footnote changes. | **See attachment 1** | The Elisa test is no longer required to be performed.  This inclusion criteria amended with amendment # 2, dated 6 November 2002.  Footnote numbering sequence changes as a result of this deletion. |
| TP 21, Section 4.1.3, Dosage and treatment schedule, paragraph 5, 2^nd^ sentence. | Should a mother have been randomised to any treatment arm in false labour, and later discharged, she is to receive the same previously randomised treatment, when presenting in active labour, with the clock reset to time zero.  **Amended to:**  Should a mother have been randomised to a nevirapine plus Combivir® arm in false labour, and later be discharged, she is to receive the same previously randomised treatment when presenting in active labour, if more than 5 days have passed since the initial presentation. The study clock will reset to time zero and mothers will be given the full course of nevirapine and Combivir®, to which they were previously randomised, i.e, a single dose of nevirapine 200mg and Combivir® given as one tablet bid while in labour, followed by Combivir®, given as one tablet bid, for either 4 or 7 days.  Should a mother however return in active labour within 5 days or less since her initial presentation in false labour, she is to receive Combivir®, given as one tablet for every 12 hours of labour. Following delivery these mothers are to complete the 4 or 7 day course of Combivir® tablets started during the initial presentation in false labour. The Combivir® will be taken as one tablet bid in the period between discharge for false labour and presentation in active labour, as well as during the post delivery period in order to complete the full course of Combivir®(either 4 or 7 days of treatment). If the mother was randomised to the 4 day Combivir® group during her false labour, and has taken the full 4 day course of Combivir® up to and during her active labour, she will not need to continue Combivir® in the post delivery period. | The aim is to provide functional short course antiretroviral triple therapy in an attempt to reduce the rate of resistance mutations in mothers. Due to the long half life of nevirapine, provision of a second dose would only be required if more than 5 days passed between the initial presentation of the mother in false labour and the presentation in active labour. |
| TP 21, Section 4.1.3, Dosage and treatment schedule, paragraph 6. | Mothers randomised to the nevirapine only arm in false labour > 72 hours before presenting in active labour, will receive a second dose of nevirapine when presenting in active labour, but will be excluded from the primary analyses.  **Amended to:**  Mothers randomised to the nevirapine only arm in false labour **will not receive a second dose of nevirapine** when presenting in active labour. **Neonates born to these mothers will receive a dose of nevirapine immediately after delivery and a second dose of nevirapine 24 to 72 hours post delivery or immediately prior to discharge.** | In this setting of short course monotherapy, provision of a second dose of nevirapine to the mother may be associated with an increased rate of resistance, which should be avoided wherever possible. This change brings the protocol more in line with current recommended treatment guidelines in South Africa. |
| TP 22, Section 4.1.3, Dosage and treatment schedule, paragraph 8, 3^rd^ sentence. | The neonate will be administered a 2mg/kg dose of nevirapine immediately post delivery if the mother was dosed within 2 hours of delivery or did not receive any medication prior to delivery and given a second dose 24 to 72 hours after birth.  **Amended to:**  If the mother was dosed within 2 hours of delivery, or if the mother did not receive any medication prior to delivery, the neonate will be administered a 2mg/kg dose of nevirapine immediately post delivery and will be given a second dose of nevirapine 24 to 72 hours after birth **or immediately prior to discharge.** | To clarify that the second dose may be administered just prior to discharge of the patient at academic hospital sites with rapid patient turnaround time. |
| TP 33, Section 6.2, Study procedures at each visit, Mothers | **Visit 3.1: Day 8 ±1 (drug accountability visit)**  **Amended to:**  **Visit 3.1: Day 4 to 9 (drug accountability visit)** | By allowing greater flexibility as to when this visit may be scheduled, patient compliance will be improved. |

**Attachment 1:**

**Flow chart changes**

MOTHER

| Visit Number | 1 | 1.1 | 2 | 3 | 3.1 ^12^ | 4 | **5^2, 3^** | **6**** | **7**** | **7.1** | **8^5, 8^**** | 9*** |  |
| --- | --- | --- | --- | --- | --- | --- | --- | --- | --- | --- | --- | --- | --- |
|  | Screening  (Prenatal) | Screening Eligi-  bility  Visit | Enrollment  (Labour and delivery) |  | Drug Account-ability Visit |  |  |  |  |  |  |  | End of trial |
| Day | ≥ 34 weeks gestation | >Gest. age 34 w to Day 0 | 0 | 1-2 | 8 (±1) | 14  (± 2) | 42  (± 6) | 90  (± 10) | 168  (± 10) | 252  (± 10) | 336  (± 14) | 504  (± 14) |  |
| Informed Consent | X |  |  |  |  |  |  |  |  |  |  |  |  |
| HIV ELISA Test | X^9^ |  |  |  |  |  |  |  |  |  |  |  |  |
| Demographics | X |  |  |  |  |  |  |  |  |  |  |  |  |
| Review Inclusion/ Exclusion criteria | X | X | X |  |  |  |  |  |  |  |  |  |  |
| Medical History | X |  |  |  |  |  |  |  |  |  |  |  |  |
| Randomisation |  |  | X |  |  |  |  |  |  |  |  |  |  |
| Post delivery history |  |  |  | X |  |  |  |  |  |  |  |  |  |
| HIV related symptoms and signs | X |  |  | X |  |  | X | X | X | X | X | X |  |
| Physical Examination | X |  |  | X |  |  | X | X | X | X | X | X |  |
| Viral load PCR (RNA) | X |  | X | X |  | X | X | X | X | X | X | X |  |
| CD4 | X |  | X | X |  | X | X | X | X | X | X | X |  |
| Laboratory Tests ^4^ | X |  | X | X |  | X | X |  |  |  |  |  |  |
| Labour and Delivery History |  |  | X |  |  |  |  |  |  |  |  |  |  |
| Adverse Events | X | X | X | X | X | X | X | X | X | X | X | X |  |
| Concomitant Therapy | X | X | X | X | X | X | X | X | X | X | X | X |  |
| Drug Administration |  |  | X^1^ | X^1^ |  |  |  |  |  |  |  |  |  |
| Drug accountability/ compliance |  |  | X | X | X | X |  |  |  |  |  |  |  |
| Sampling for Resistance testing |  |  | X | X |  | X | X | X | X | X^11^ | X | X |  |
| End of trial admin/trial completion |  |  |  |  |  |  |  |  |  |  |  |  | X^2,3,5,10^ |

**Amended to:**

MOTHER

| Visit Number | 1 | 1.1 | 2 | 3 | 3.1^11^ | 4 | **5^2, 3^** | **6**** | **7**** | **7.1**** | **8^5, 8^**** | 9*** |  |
| --- | --- | --- | --- | --- | --- | --- | --- | --- | --- | --- | --- | --- | --- |
|  | Screening  (Prenatal) | Screening Eligi-  bility  Visit | Enrollment  (Labour and delivery) |  | Drug Account-ability Visit |  |  |  |  |  |  |  | End of trial |
| Day | ≥ 34 weeks gestation | >Gest. age 34 w to Day 0 | 0 | 1-2 | 4 to 9 | 14  (± 2) | 42  (± 6) | 90  (± 10) | 168  (± 10) | 252  (± 10) | 336  (± 14) | 504  (± 14) |  |
| Informed Consent | X |  |  |  |  |  |  |  |  |  |  |  |  |
| Demographics | X |  |  |  |  |  |  |  |  |  |  |  |  |
| Review Inclusion/ Exclusion criteria | X | X | X |  |  |  |  |  |  |  |  |  |  |
| Medical History | X |  |  |  |  |  |  |  |  |  |  |  |  |
| Randomisation |  |  | X |  |  |  |  |  |  |  |  |  |  |
| Post delivery history |  |  |  | X |  |  |  |  |  |  |  |  |  |
| HIV related symptoms and signs | X |  |  | X |  |  | X | X | X | X | X | X |  |
| Physical Examination | X |  |  | X |  |  | X | X | X | X | X | X |  |
| Viral load PCR (RNA) | X |  | X | X |  | X | X | X | X | X | X | X |  |
| CD4 | X |  | X | X |  | X | X | X | X | X | X | X |  |
| Laboratory Tests ^4^ | X |  | X | X |  | X | X |  |  |  |  |  |  |
| Labour and Delivery History |  |  | X |  |  |  |  |  |  |  |  |  |  |
| Adverse Events | X | X | X | X | X | X | X | X | X | X | X | X |  |
| Concomitant Therapy | X | X | X | X | X | X | X | X | X | X | X | X |  |
| Drug Administration |  |  | X^1^ | X^1^ |  |  |  |  |  |  |  |  |  |
| Drug accountability/ compliance |  |  | X | X | X | X |  |  |  |  |  |  |  |
| Sampling for Resistance testing |  |  | X | X |  | X | X | X | X | X^10^ | X | X |  |
| End of trial admin/trial completion |  |  |  |  |  |  |  |  |  |  |  |  | X^2,3,5,9^ |

INFANT

| **Visit Number** | **2** | **3** | **3.1^12^** | **4** | **5^2 ,3^** | **5.1*** | **6**** | **7**** | **7.1** | **8^58^**** | **9***** |  |
| --- | --- | --- | --- | --- | --- | --- | --- | --- | --- | --- | --- | --- |
| **Day** | **0** | **1-2**  **(within 0-72 hrs)** | **8**  **± 1** | **14**  **(± 2)** | **42**  **(± 6)** | **49**  **(± 6)** | **90**  **(± 10)** | **168**  **(± 10)** | **252**  **(± 10)** | **336**  **(± 14)** | **504**  **(± 14)** | **End of Trial** |
| Neonatal History (Including Apgar score) |  | X |  |  |  |  |  |  |  |  |  |  |
| Patient Demographics |  | X |  |  |  |  |  |  |  |  |  |  |
| Eligibility criteria |  | X |  |  |  |  |  |  |  |  |  |  |
| Record infant feeding method |  | X |  | X | X |  | X | X | X | X | X |  |
| Physical Examination |  | X |  | X | X |  | X | X | X | X | X |  |
| HIV related symptoms and signs |  |  |  |  | X |  | X |  |  | X | X |  |
| Laboratory Tests^4^ |  | X |  | X | X |  |  |  |  |  |  |  |
| PCR (RNA) |  | X |  | X | X | X | X | X | X | X | X |  |
| PCR (DNA) |  | X^7^ |  | X | X² | X |  |  |  |  |  |  |
| Drug Administra-tion |  | X^1^, ^6^ |  |  |  |  |  |  |  |  |  |  |
| Drug Accountability/  compliance |  | X | X | X |  |  |  |  |  |  |  |  |
| Adverse Events |  | X | X | X | X |  | X | X | X | X | X |  |
| Concomitant Therapy |  | X | X | X | X |  | X | X | X | X | X |  |
| Sampling for Resistance testing |  | X |  | X | X | X | X | X | X^11^ | X | X |  |
| End of trial admin/trial completion |  |  |  |  |  |  |  |  |  |  |  | X^2,3,.5,10^ |

1. All mothers to receive a single dose of nevirapine in labour and will be randomised to either no Combivir® or 4 or 7 days of Combivir®, also to be administered while in labour. Infants to receive the same treatment as mother.

1. Visit 5 concludes patient participation if mother and infant do not demonstrate resistance or the infant remains HIV DNA PCR negative.
2. Visit 5 also to be completed, if possible, for all dropouts and withdrawals prior to visit 5.

4. Laboratory tests refer to: Full blood count, serum creatinine, AST, ALT, ALP, Total bilirubin, amylase.

5. Visit 8 concludes mother and infant participation in the trial for those mothers and infants with demonstrated resistance after visit 5, but with no demonstrated resistance at visit 8.

6. Retrovir® and 3TC® administered to infant within 24 hours after birth, nevirapine to be administered 24-72 hours after birth, or just prior to discharge if hospital stay is less than 24 hours.

7. Initial HIV DNA PCR to be performed within 48 hours.

8. Visit 8 also to be completed, if possible, for all dropouts and withdrawals between visits 5 to 8.

1. Unless a positive HIV-1 ELISA test is documented.
2. Visit 9 will conclude participation in the trial for all mothers and infants with demonstrated resistance at visit 8.
3. The sample will be stored from this visit, and will be tested for resistance only if the patient is lost to follow up after this visit, or if there was no detectable resistance at visit 8.
4. This visit is applicable only to those patients randomised to either the 4 or 7 day CBV arm.

* Extra visit for infants who test HIV DNA PCR positive for first time at visit 5.

****** Only for those patients with resistant virus.

******* Visit 9 is intended for those patients with demonstrated genotypic resistance at visit 8.

**Amended to:**

INFANT

| **Visit Number** | **2** | **3** | **3.1^11^** | **4** | **5^2 ,3^** | **5.1*** | **6**** | **7**** | **7.1**** | **8^58^**** | **9***** |  |
| --- | --- | --- | --- | --- | --- | --- | --- | --- | --- | --- | --- | --- |
| **Day** | **0** | **1-2**  **(within 0-72 hrs)** | **4 to 9** | **14**  **(± 2)** | **42**  **(± 6)** | **49**  **(± 6)** | **90**  **(± 10)** | **168**  **(± 10)** | **252**  **(± 10)** | **336**  **(± 14)** | **504**  **(± 14)** | **End of Trial** |
| Neonatal History (Including Apgar score) |  | X |  |  |  |  |  |  |  |  |  |  |
| Patient Demographics |  | X |  |  |  |  |  |  |  |  |  |  |
| Eligibility criteria |  | X |  |  |  |  |  |  |  |  |  |  |
| Record infant feeding method |  | X |  | X | X |  | X | X | X | X | X |  |
| Physical Examination |  | X |  | X | X |  | X | X | X | X | X |  |
| HIV related symptoms and signs |  |  |  |  | X |  | X |  |  | X | X |  |
| Laboratory Tests^4^ |  | X |  | X | X |  |  |  |  |  |  |  |
| PCR (RNA) |  | X |  | X | X | X | X | X | X | X | X |  |
| PCR (DNA) |  | X^7^ |  | X | X² | X |  |  |  |  |  |  |
| Drug Administra-tion |  | X^1^, ^6^ |  |  |  |  |  |  |  |  |  |  |
| Drug Accountability/  compliance |  | X | X | X |  |  |  |  |  |  |  |  |
| Adverse Events |  | X | X | X | X |  | X | X | X | X | X |  |
| Concomitant Therapy |  | X | X | X | X |  | X | X | X | X | X |  |
| Sampling for Resistance testing |  | X |  | X | X | X | X | X | X^10^ | X | X |  |
| End of trial admin/trial completion |  |  |  |  |  |  |  |  |  |  |  | X^2,3,.5,9^ |

1. All mothers to receive a single dose of nevirapine in labour and will be randomised to either no Combivir® or 4 or 7 days of Combivir®, also to be administered while in labour. Infants to receive the same treatment as mother.
2. Visit 5 concludes patient participation if mother and infant do not demonstrate resistance or the infant remains HIV DNA PCR negative.
3. Visit 5 also to be completed, if possible, for all dropouts and withdrawals prior to visit 5.
4. Laboratory tests refer to: Full blood count, serum creatinine, AST, ALT, ALP, Total bilirubin, amylase.
5. Visit 8 concludes mother and infant participation in the trial for those mothers and infants with demonstrated resistance after visit 5, but with no demonstrated resistance at visit 8.

6. Retrovir® and 3TC® administered to infant within 24 hours after birth, nevirapine to be administered 24-72 hours after birth, or just prior to discharge if hospital stay is less than 24 hours.

7. Initial HIV DNA PCR to be performed within 48 hours.

8. Visit 8 also to be completed, if possible, for all dropouts and withdrawals between visits 5 to 8.

9. Visit 9 will conclude participation in the trial for all mothers and infants with demonstrated resistance at visit 8.

10. The sample will be stored from this visit, and will be tested for resistance only if the patient is lost to follow up after this visit, or if there was no detectable resistance at visit 8.

11.This visit is applicable only to those patients randomised to either the 4 or 7 day CBV arm.

* Extra visit for infants who test HIV DNA PCR positive for first time at visit 5.

****** Only for those patients with resistant virus.

******* Visit 9 is intended for those patients with demonstrated genotypic resistance at visit 8.
