# Supplementary material for: Efficacy of Short-Course AZT Plus 3TC to Reduce Nevirapine Resistance in the Prevention of Mother-to-Child HIV Transmission: A Randomized Clinical Trial
Source: PLoS Med. 2009 Oct 27;6(10):e1000172. doi: 10.1371/journal.pmed.1000172 (PMC2760761; doi:10.1371/journal.pmed.1000172)
Supplement: Text S5 — Protocol amendment 4. (0.07 MB DOC) [file pmed.1000172.s005.doc]

Boehringer Ingelheim (Pty), Ltd.

ABCD

Boehringer Ingelheim (Pty), Ltd

404 Main Ave, Ferndale, Randburg, South Africa

**Clinical Trial Protocol** **Amendment**

| **Amendment Number:**  **Date:** | | | | 4 | | |  | | | |
| --- | --- | --- | --- | --- | --- | --- | --- | --- | --- | --- |
|  |  |  |  | 12 May 2003 | | |  | | |  |
| Trial No.: | | | 1100.1413 | | | ⌧ | | | Implemented only after documented approval of IRB / IEC | |
| Test Substance(s) | | | Nevirapine | | |  | | | Implemented immediately in order to eliminate immediate hazard IRB / IEC to be notified of change with request for approval | |
|  | | |  | | |  | | | Implemented immediately as changes involve only logistical or administrative aspects. IRB / IEC notified of changes only | |
| Title: | | | An Open-label Study evaluating the Resistance profile of Single dose Nevirapine (NVP) when combined with a 4 or 7 day course of Combivir® (ZDV/3TC) compared to Single dose Nevirapine for the Prevention of Mother to Child Transmission (pMTCT) of HIV - Treatment Options Preservation Study (T.O.P.S.) | | | | | | | |
| Changes: | | | Please see attached pages | | | | | | | |
| Reason For Change: | | 1. To delete 2 exclusion criteria. 2. To further clarify the planned primary analyses. | | | | | | | | |
|  | | | | |  | | | **Page 1 of 4** | | |
| Confidential | © Boehringer Ingelheim  This protocol is the property of Boehringer Ingelheim and may not - in full or in part - be passed on, reproduced, published or otherwise used without the express permission of Boehringer Ingelheim | | | | | | | | | |

# PROTOCOL AMENDMENT SIGNATURE PAGE

| **BI Trial No.:** | 1100.1413 | | |  | | |
| --- | --- | --- | --- | --- | --- | --- |
| **Amendment No.:** | 4 | | |  | | |
| Trial Clinical Monitor: | |  |  | |  |  |
| Name  Organisation/Department | |  | date | |  | Dr. John Steytler  Boehringer Ingelheim (Pty) Ltd / Medical Dept. |
| Trial Statistician: (indicate early information on signature, if applicable) | |  |  | |  |  |
| Name  Organisation/Department | |  | date | |  | Mr. Toshio Kimura  Boehringer Ingelheim Pharmaceuticals, Inc./ Biometrics  and Data Management |
| Medical Director: | |  |  | |  |  |
| Name  Organisation/Department | |  | date | |  | Dr. Lynette Boshoff  Boehringer Ingelheim (Pty) Ltd / Medical Dept. |
| Team Member Medicine: (indicate early information on approval, if applicable) | |  |  | |  |  |
| Name  Organisation/Department | |  | date | |  | Dr. Michael Imperiale  Boehringer Ingelheim Pharmaceuticals, Inc. / Clinical Research |
| I herewith certify that I agree to adhere to the amended trial protocol and to all documents referenced in the amended trial protocol. | | | | | | |
| Investigator: | |  |  | |  |  |
| Name | |  | date | |  |  |
| Organisation/Department | |  |  | |  |  |

| **Page**  **(Section Number)** | **Changes** | **Reason for Change** |
| --- | --- | --- |
| TP 17, Section 3.3,  Exclusion criteria | **Deleted:**  **Mothers who are not able to take oral medication.** | Provision is already made in the protocol for infant dosing immediately post delivery, with a second dose of nevirapine given later, to compensate for the absence of an intrapartum dose to the mother as is currently the practice outside of the clinical trial setting. The infants of these women should not be denied access to study treatment on this basis alone. |
| TP 18, Section 3.3,  Exclusion criteria | **Deleted:**  **Mothers who present with an obstetric emergency, the sequelae of which in the investigators opinion will not permit oral dosing of study medication.** | Provision is already made in the protocol for infant dosing immediately post delivery, with a second dose of nevirapine given later, to compensate for the absence of an intrapartum dose to the mother as is currently the practice outside of the clinical trial setting. The infants of these women should not be denied access to study treatment on this basis alone. |
| TP 38, Section 7.3.1, Primary analyses. | Treatment groups will be compared using the Fisher's Exact test to perform pairwise comparisons. All mothers with genotypic testing results at least 2 weeks and no more than 8 weeks after delivery will be included in the analysis.  **Amended to:**  Treatment groups will be compared using the Fisher's Exact test to perform pairwise comparisons. All mothers who meet the eligibility criteria, completed intrapartum dosing of study medication as per protocol and who have genotypic testing results at least 2 weeks and no more than 8 weeks after delivery will be included in the analysis. | The primary objective is to demonstrate a reduced rate of resistance to nevirapine by adding a Combivir® regimen to nevirapine intrapartum dosing of pregnant women when they present in labour. Hence women who have not received an intrapartum dose of study treatment can logically not be included in the planned analysis. |
