# Supplementary material for: Efficacy of Short-Course AZT Plus 3TC to Reduce Nevirapine Resistance in the Prevention of Mother-to-Child HIV Transmission: A Randomized Clinical Trial
Source: PLoS Med. 2009 Oct 27;6(10):e1000172. doi: 10.1371/journal.pmed.1000172 (PMC2760761; doi:10.1371/journal.pmed.1000172)
Supplement: Text S6 — Protocol amendment 5. (0.06 MB DOC) [file pmed.1000172.s006.doc]

Boehringer Ingelheim (Pty), Ltd.

ABCD

Boehringer Ingelheim (Pty), Ltd

404 Main Ave, Ferndale, Randburg, South Africa

**Clinical Trial Protocol** **Amendment**

| **Amendment Number:**  **Date:** | | | | 5 | | |  | | | |
| --- | --- | --- | --- | --- | --- | --- | --- | --- | --- | --- |
|  |  |  |  | 20 June 2003 | | |  | | |  |
| Trial No.: | | | 1100.1413 | | | ⌧ | | | Implemented only after documented approval of IRB / IEC | |
| Test Substance(s) | | | Nevirapine | | |  | | | Implemented immediately in order to eliminate immediate hazard IRB / IEC to be notified of change with request for approval | |
|  | | |  | | |  | | | Implemented immediately as changes involve only logistical or administrative aspects. IRB / IEC notified of changes only | |
| Title: | | | An Open-label Study evaluating the Resistance profile of Single dose Nevirapine (NVP) when combined with a 4 or 7 day course of Combivir® (ZDV/3TC) compared to Single dose Nevirapine for the Prevention of Mother to Child Transmission (pMTCT) of HIV - Treatment Options Preservation Study (T.O.P.S.) | | | | | | | |
| Changes: | | | Please see attached pages | | | | | | | |
| Reason For Change: | | 1. To amend an exclusion criterion. | | | | | | | | |
|  | | | | |  | | | **Page 1 of 3** | | |
| Confidential | © Boehringer Ingelheim  This protocol is the property of Boehringer Ingelheim and may not - in full or in part - be passed on, reproduced, published or otherwise used without the express permission of Boehringer Ingelheim | | | | | | | | | |

# PROTOCOL AMENDMENT SIGNATURE PAGE

| **BI Trial No.:** | 1100.1413 | | |  | | |
| --- | --- | --- | --- | --- | --- | --- |
| **Amendment No.:** | 5 | | |  | | |
| Trial Clinical Monitor: | |  |  | |  |  |
| Name  Organisation/Department | |  | date | |  | Dr. John Steytler  Boehringer Ingelheim (Pty) Ltd / Medical Dept. |
| Trial Statistician: (indicate early information on signature, if applicable) | |  |  | |  |  |
| Name  Organisation/Department | |  | date | |  | Mr. Toshio Kimura  Boehringer Ingelheim Pharmaceuticals, Inc./ Biometrics  and Data Management |
| Medical Director: | |  |  | |  |  |
| Name  Organisation/Department | |  | date | |  | Dr. Mark Hopley  Boehringer Ingelheim (Pty) Ltd / Medical Dept. |
| Team Member Medicine: (indicate early information on approval, if applicable) | |  |  | |  |  |
| Name  Organisation/Department | |  | date | |  | Dr. Patrick Robinson for Dr. Michael Imperiale  Boehringer Ingelheim Pharmaceuticals, Inc. / Clinical Research |
| I herewith certify that I agree to adhere to the amended trial protocol and to all documents referenced in the amended trial protocol. | | | | | | |
| Investigator: | |  |  | |  |  |
| Name | |  | date | |  |  |
| Organisation/Department | |  |  | |  |  |

| **Page**  **(Section Number)** | **Changes** | **Reason for Change** |
| --- | --- | --- |
| TP 18, Section 3.3,  Exclusion criteria | Patients with any one of the following additional laboratory abnormalities at screening :  Haemoglobin concentration < 9.0 g/dl.  **Amended to:**  Patients with any one of the following additional laboratory abnormalities at screening :  Haemoglobin concentration < 7.5 g/dl. | The safety concern regarding a potential zidovudine induced anaemia is more applicable in the setting of long term therapy for HIV infection. The short course of zidovudine administered in this trial, either 4 or 7 days of therapy, is unlikely to adversely affect pregnant HIV infected women with anaemia. Furthermore the zidovudine induced anaemia has been shown to be fully reversible after withdrawal of the drug. This amendment will bring the haemoglobin exclusion criteria more in line with other similar trials in the setting of the prevention of mother to child transmission of HIV. |
