# Supplementary material for: Efficacy of Short-Course AZT Plus 3TC to Reduce Nevirapine Resistance in the Prevention of Mother-to-Child HIV Transmission: A Randomized Clinical Trial
Source: PLoS Med. 2009 Oct 27;6(10):e1000172. doi: 10.1371/journal.pmed.1000172 (PMC2760761; doi:10.1371/journal.pmed.1000172)
Supplement: Text S7 — Protocol amendment 6. (0.14 MB DOC) [file pmed.1000172.s007.doc]

Boehringer Ingelheim (Pty), Ltd

404 Main Ave, Ferndale, Randburg, South Africa

ABCD

**Clinical Trial Protocol** **Amendment**

| **Amendment Number:**  **Date:** | | | | 6 | | |  | | | |
| --- | --- | --- | --- | --- | --- | --- | --- | --- | --- | --- |
|  |  |  |  | 7 June 2004 | | |  | | |  |
| Trial No.: | | | 1100.1413 | | | ⌧ | | | Implemented only after documented approval of IRB / IEC | |
| Test Substance(s) | | | Nevirapine | | |  | | | Implemented immediately in order to eliminate immediate hazard IRB / IEC to be notified of change with request for approval | |
|  | | |  | | |  | | | Implemented immediately as changes involve only logistical or administrative aspects. IRB / IEC notified of changes only | |
| Title: | | | An Open-label Study evaluating the Resistance profile of Single dose Nevirapine (NVP) when combined with a 4 or 7 day course of Combivir® (ZDV/3TC) compared to Single dose Nevirapine for the Prevention of Mother to Child Transmission (pMTCT) of HIV - Treatment Options Preservation Study (T.O.P.S.) | | | | | | | |
| Changes: | | | Please see attached pages. | | | | | | | |
| Reason For Change: | | Information collected from the first 61 patients included in this clinical trial has shown statistically significantly lower viral resistance in the patients randomised to the combination arms (9%) as compared to the single dose nevirapine arm (50%) This interim analysis shows that the primary endpoint has been met for the single dose nevirapine arm; however the combination therapy of the nevirapine plus Combivir® either for 4 or 7 days still needs to be evaluated. This analysis was not planned but after reviewing information recently disclosed it was deemed necessary to review accumulated data to date ^18^  This amendment aims to terminate randomisation into the single dose nevirapine only arm due to the primary endpoint having been met. | | | | | | | | |
|  | | | | |  | | | **Page 1 of 13** | | |
| Confidential | © Boehringer Ingelheim  This protocol is the property of Boehringer Ingelheim and may not - in full or in part - be passed on, reproduced, published or otherwise used without the express permission of Boehringer Ingelheim | | | | | | | | | |

# PROTOCOL AMENDMENT SIGNATURE PAGE

| **BI Trial No.:** | 1100.1413 | | |  | | |
| --- | --- | --- | --- | --- | --- | --- |
| **Amendment No.:** | 6 | | |  | | |
| Trial Clinical Monitor: | |  |  | |  |  |
| Name  Organisation/Department | |  | date | |  | Dr. Julia Botha  Boehringer Ingelheim (Pty) Ltd / Medical Dept. |
| Trial Statistician: (indicate early information on signature, if applicable) | |  |  | |  |  |
| Name  Organisation/Department | |  | date | |  | Mr. Toshio Kimura  Boehringer Ingelheim Pharmaceuticals, Inc./ Biometrics  and Data Management |
| Medical Director: | |  |  | |  |  |
| Name  Organisation/Department | |  | date | |  | Dr. Mark Hopley  Boehringer Ingelheim (Pty) Ltd / Medical Dept. |
| Team Member Medicine: (indicate early information on approval, if applicable) | |  |  | |  |  |
| Name  Organisation/Department | |  | date | |  | Dr. Jon Leith  Boehringer Ingelheim Pharmaceuticals, Inc. / Clinical Research |
| I herewith certify that I agree to adhere to the amended trial protocol and to all documents referenced in the amended trial protocol. | | | | | | |
| Investigator: | |  |  | |  |  |
| Name | |  | date | |  |  |
| Organisation/Department | |  |  | |  |  |

| **Page**  **(Section Number)** | **Changes** | **Reason for Change** |
| --- | --- | --- |
| TP 1  Trial Clinical Monitor | Dr. J. Steytler  **Amended to:**  Dr. J. Botha | Dr. Julia Botha is the current Trial Clinical Monitor for this study. |
| TP1  Planned Dates of Trial: | October 2002 – June 2004  **Amended to:**  October 2002 – July 2006 | The planned end date for this trial has been extended due to the increase in the required number of patients, difficulties in recruitment of patients and the MCC’s request to extend the observation period of the resistant patients to 18 months. See Amendment 1. |
| TP 2  Clinical Monitor Local | Dr. J. Steytler  **Amended to:**  Dr. J. Botha | Dr. Julia Botha is the current Trial Clinical Monitor for this study. |
| TP 4  Objectives | To determine whether a regimen of single dose nevirapine combined with either 4 or 7 days of Combivir®, compared to a regimen of single dose nevirapine, for the prevention of mother to child transmission can reduce the rate of development of drug resistant mutations of HIV-1, in HIV-1 infected pregnant women, who have not received antiretroviral therapy previously.  **Amended to:**  Initial Objective:  To determine whether a regimen of single dose nevirapine combined with either 4 or 7 days of Combivir®, compared to a regimen of single dose nevirapine, for the prevention of mother to child transmission can reduce the rate of development of drug resistant mutations of HIV-1, in HIV-1 infected pregnant women, who have not received antiretroviral therapy previously.  Subsequent Objectives:  An interim analysis of the first 61 patients showed that a clinical and statistical difference exists between the occurrence of HIV-1 NNRTI resistant mutations in the single dose nevirapine only arm (50%) and the two other combination arms (9%). These findings partially answered the objectives outlined in the initial objectives. Consequently enrolment onto the single dose nevirapine arm was terminated. The objective of the trial was modified to compare whether either the 4 or the 7 day combination of Combivir® and nevirapine would result in any significant reduction in the incidence of nevirapine resistance. | Following the results of the interim analysis |
| TP 4  No. of subjects total:  Each treatment: | 300 HIV infected pregnant women and their infants randomised to a 1:1:1 ratio.  **Amended to:**  360 - 390 HIV infected pregnant women and their infants.  Following the results of the unplanned interim analysis the design has been amended to include 150 patients for each of the two combination arms (nevirapine plus Combivir® for 4 days and 7 days). The mothers will be randomised in a 1:1 ratio, once the nevirapine arm had been closed. The balance of the patients relate to those patients already enrolled into the single dose nevirapine arm. | Following the results of the interim analysis |
| TP 4  Duration of Treatment: | Mothers to receive a single dose of nevirapine or a single dose of nevirapine and either 4 or 7 days of Combivir®.  Infants to receive a single dose of nevirapine or a single dose of nevirapine and either 4 or 7 days of zidovudine and 3TC.  **Amended to:**  Mothers to receive nevirapine and either 4 or 7 days of Combivir®. The single dose nevirapine arm will be discontinued following amendment 6.  Infants to receive nevirapine and either 4 or 7 days of Zidovudine and 3TC. The single dose nevirapine arm to be discontinued following amendment 6. | Following the results of the interim analysis |
| TP 6  Footnote 1 | All mothers to receive a single dose of nevirapine in labour and will be randomised to either no Combivir® or 4 or 7 days of Combivir®, also to be administered while in labour. Infants to receive the same treatment as mother.  Amended to:  All mothers to receive a single dose of nevirapine in labour and will be randomised to either 4 or 7 days of Combivir®, also to be administered while in labour. Infants to receive the same treatment as mother. | Following the results of the interim analysis the single dose nevirapine arm has been terminated |
| TP 15  Section 1.3  Description and Rationale for Performing the Trial | An additional paragraph was inserted after the third paragraph.  Added:  An interim analysis showed that the addition of Combivir® did result in decreased emergence of viral resistance to nevirapine. The trial will be continued with further recruitment into the two combination arms in order to assess the differences between the additions of 4 vs 7 days of Combivir® to single dose | Following the results of the interim analysis the single dose nevirapine arm has been closed |
| TP 15  Section 1.3  Description and Rationale for Performing the Trial  Last paragraph | The study will be conducted as an open label three arm study involving a population of antiretroviral naïve HIV infected pregnant women and their newborn infants. Only mothers with a viral load of > 2000 copies/mL will be included in the study.  **Amended to:**  Initially the study was conducted as an open label three arm study, however following amendment 6 it will be a two armed study, involving a population of antiretroviral naïve HIV infected pregnant women and their newborn infants. Only mothers with a viral load of > 2000 copies/mL will be included in the study. | Following the results of the interim analysis the single dose nevirapine arm has been terminated |
| TP 16  Section 2.1  General Aim/ Primary Objective | To determine whether a regimen of single dose nevirapine combined with either 4 or 7 days of Combivir®, compared to a regimen of single dose nevirapine, for the prevention of mother to child transmission can reduce the rate of development of drug resistant mutations of HIV-1, in HIV-1 infected pregnant women, who have not received antiretroviral therapy previously. Amended to: To determine whether a regimen of single dose nevirapine combined with either 4 or 7 days of Combivir®, compared to a regimen of single dose nevirapine, for the prevention of mother to child transmission can reduce the rate of development of drug resistant mutations of HIV-1, in HIV-1 infected pregnant women, who have not received antiretroviral therapy previously.An interim analysis of the first 61 patients showed that a clinical and statistical difference between the occurrence between HIV-1 resistant mutations in the single dose nevirapine only arm (50%) and the two other combination arms (9%). These findings partially answered the objectives outlined in the initial objectives. Consequently enrolment onto the single dose nevirapine arm was terminated. The objective of the trial was modified to compare whether either the 4 or the 7 day combination of Combivir® and nevirapine would result in any significant reduction in the incidence of nevirapine resistance. | Following the results of the interim analysis |
| TP 16  Section 3.1  Number of Subjects Planned | The total trial population will consist of 300 HIV-1-infected pregnant women, who meet the inclusion and exclusion criteria, and their neonates to achieve 240 mother-infant pairs who are evaluable. Patient participation will be for 6 weeks unless resistance is demonstrated in which case patients will be followed up for 12 months. All patients demonstrating resistance after 12 months will be followed for a further 6 month period. Five study centres in South Africa are envisaged to provide a total of 300 patients.  **Amended to:**  The total trial population will consist of 360 - 390 HIV infected pregnant women, who meet the inclusion and exclusion criteria, and their infants.  Following the results of the interim analysis the design has been amended to include 150 evaluable patients for each of the two combination arms (single dose nevirapine plus Combivir® for 4 days and 7 days). The balance of the patients relate to those patients already enrolled into the single dose nevirapine arm. | Following the results of the interim analysis |
| TP 19  Section 4.1.3  Dosage and Treatment Schedules  Point 1 | 1. A single dose of a nevirapine 200mg tablet during labour.   **Amended to:**   1. A single dose of a nevirapine 200mg tablet during labour. Following Amnedment 6 this arm was closed. | Following the results of the interim analysis |
| TP 19  Section 4.1.3  Dosage and Treatment Schedules | Mothers randomised to the nevirapine only arm in false labour **will not receive a second dose of nevirapine** when presenting in active labour. **Neonates born to these mothers will receive a dose of nevirapine immediately after delivery and a second dose of nevirapine 24 to 72 hours post delivery or immediately prior to discharge.**  **Amended to:**  During recruitment into the nevirapine only arm the following procedure was followed. Mothers randomised to the nevirapine only arm in false labour **did not receive a second dose of nevirapine** when presenting in active labour. **Neonates born to these mothers received a dose of nevirapine immediately after delivery and a second dose of nevirapine 24 to 72 hours post delivery or immediately prior to discharge.** | Following the results of the interim analysis |
| TP 20  Section 4.1.3  Dosage and Treatment Schedules  Point 1 for Neonates | 1. A single dose of 2mg/kg nevirapine suspension within 24- 72 hours after birth or just prior to discharge if hospital stay is less than 24 hours.  **Amended to:**  1. A single dose of 2mg/kg nevirapine suspension within 24- 72 hours after birth or just prior to discharge if hospital stay is less than 24 hours. Following a preliminary analysis, recruitment into this arm was not continued (Amendment 6). | Following the results of the interim analysis. |
| TP 28  Section 6.1  Mothers, Point 1 | 1. A single dose of a nevirapine 200mg tablet during labour.   **Amended to:**  1. A single dose of a nevirapine 200mg tablet during labour. Recruitment into this arm was stopped follwing a preliminary analysis that showed clinically and statistically fewer resistant HIV-1 mutants in the combination arms described below (Amendment 6). | Following the results of the interim analysis. |
| TP 29  Section 6.1  Neonates, Point 1 | 1. A single dose of 2mg/kg nevirapine suspension within 24- 72 hours after birth.   **Amended to:**   1. A single dose of 2mg/kg nevirapine suspension within 24- 72 hours after birth. This arm will be discontinued following the interim analysis (Amendment 6). | Following the results of the interim analysis. |
| TP 38  Section 7  Statistics | **Added:**  Based on an interim analysis, the design of this trial has been changed to terminate the single dose nevirapine arm but to continue randomization of patients into one of two treatment groups: single dose nevirapine plus either 1) Combivir® for 4 days or 2) plus Combivir® for 7 days. | Following the results of the interim analysis. |
| TP 38  Section 7.2  Null and Alternative Hypotheses | The null hypothesis is that differences in development of resistance-associated mutations are a result of random variation and that the expected proportion of mothers developing one or more resistance associated reverse transcriptase (RT) mutation is the same for all three treatment groups. Alternate hypotheses, tested sequentially, are that 7 day Combivir® treatment has a lower proportion of mothers developing reverse transcriptase mutations than no Combivir®, followed by 4 day Combivir® treatment has a lower proportion than no Combivir®.  **Amended to:**  The null hypothesis is that differences in development of resistance-associated mutations are a result of random variation and that the expected proportion of mothers developing one or more resistance associated reverse transcriptase (RT) mutation is the same for all three treatment groups. Based on the current amendment, a comparison of the proportions of patients with resistance will be made between the nevirapine plus 4 days of Combivir® and nevirapine plus 7 days of Combivir®. Alternate hypotheses, tested sequentially, are that 7 day Combivir® treatment has a lower proportion of mothers developing reverse transcriptase mutations than no Combivir®, followed by 4 day Combivir® treatment has a lower proportion than no Combivir®, followed by 7 day Combivir® treatment has a lower proportion than 4 day treatment with Combivir®. | Following the results of the interim analysis. |
| TP 38  Section 7.3.1  Primary analyses | Treatment groups will be compared using the Fisher's Exact test to perform pairwise comparisons. All mothers who meet the eligibility criteria, completed intrapartum dosing of study medication as per protocol and who have genotypic testing results at least 2 weeks and no more than 8 weeks after delivery will be included in the analysis.  **Amended to:**  Treatment groups will be compared using the Fisher's Exact test to perform pairwise comparisons. All mothers with genotypic testing results at least 2 weeks and no more than 8 weeks after delivery will be included in the analysis. | Following the results of the interim analysis. |
| TP 39  Section 7.3.4  Interim analyses | No interim analyses are planned.  **Amended to:**  No further interim analyses are planned. An analysis was performed, following release of new information , which supported the importance of resistance in subsequent response to antiretroviral treatment following Viramune®-containing mother to child prevention therapy ^18^ | Following the results of the interim analysis. |
| TP 40  Section 7.5  Randomisation | One randomisation sequence, maintaining a one to one balance between treatment arms will be generated. Treatment assignments will be maintained blinded until randomisation, only unblinding each mother/infant pair immediately before dosing of the mother. Mothers will be assigned to treatment with 7 days Combivir®, 4 days Combivir®, or no Combivir®.  **Amended to:**  One randomisation sequence, maintaining a one to one balance between treatment arms will be generated.Treatment assignments will be maintained blinded until randomisation, only unblinding each mother/infant pair immediately before dosing of the mother. Mothers will be assigned to treatment single dose nevirapine with 7 days Combivir® or 4 days Combivir®, Thereafter, mothers will be assigned to treatment with 7 days Combivir® or 4 days Combivir®. | Following the results of the interim analysis |
| TP 40  Section 7.6  Sample Size Issues | With a different genotyping process and different subtypes of HIV-1, resistance-associated mutations were seen in approximately 20% of mothers. With a different dosing regimen, 2 doses of nevirapine, HIV-1 resistance-associated mutations were seen in approximately 60% of mothers. Sample size of 80 evaluable mothers per treatment group was selected based on considering control rates of 20-30%. At 20% rate for the nevirapine-only arm, 80% power is achieved for an alpha-0.05(2-sided) test if Combivir® results in an 80% reduction to 4% in the proportion of mothers with nevirapine-associated mutations. At 25% rate for the nevirapine-only arm, 92% power is achieved for an alpha-0.05 (2-sided) test if Combivir® results in an 80% reduction to 5% in the proportion of mothers with nevirapine-associated mutations. At 30% rate for the nevirapine-only arm, 85% power is achieved for an alpha-0.05 (2-sided) test if Combivir® results in a 66% reduction to 10% in the proportion of mothers with nevirapine-associated mutations.  **Amended to:**  With a different genotyping process and different subtypes of HIV-1, resistance-associated mutations were seen in approximately 20% of mothers. With a different dosing regimen, 2 doses of nevirapine, HIV-1 resistance-associated mutations were seen in approximately 60% of mothers. Sample size of 80 evaluable mothers per treatment group was selected based on considering control rates of 20-30%. At 20% rate for the nevirapine-only arm, 80% power is achieved for an alpha-0.05(2-sided) test if Combivir® results in an 80% reduction to 4% in the proportion of mothers with nevirapine-associated mutations. At 25% rate for the nevirapine-only arm, 92% power is achieved for an alpha-0.05 (2-sided) test if Combivir® results in an 80% reduction to 5% in the proportion of mothers with nevirapine-associated mutations. At 30% rate for the nevirapine-only arm, 85% power is achieved for an alpha-0.05 (2-sided) test if Combivir® results in a 66% reduction to 10% in the proportion of mothers with nevirapine-associated mutations.  As a consequence of this amendment, the sample size for the nevirapine plus 4 days of Combivir® (sdNVP+CBV4) and for the nevirapine plus 7 days of Combivir® groups (sdNVP+CBV7) have been adjusted to a total of 150 for each treatment group (Table). The sample size was determined based on an assumption of 15% proportion of mothers demonstrating resistance by 6 weeks for the less effective treatment group of sdNVP+CBV4 or sdNVP+ CBV7. The more effective treatment group may be expected to decrease the proportion to 5%. Thus the study would have a 79% power to detect a difference with an alpha-0.05 (2-sided) test if one treatment results in a reduction from 15% reduced to 5% in the proportion of mothers with nevirapine-associated mutations. | Following the results of the interim analysis |
| TP 40 Section 7.6  Sample size issues | **Added:**  See Table below: | Following the results of the interim analysis |

**TABLE**

|  | **sdNVP** | **sdNVP+CBV4** | **sdNVP+CBV7** |
| --- | --- | --- | --- |
| **Approximate allocation of mothers at time of current amendment (1:1:1)** | **52** | **52** | **52** |
| **Additional evaluable mothers based on current amendment** | **0** | **98** | **98** |
| **Planned Total Evaluable Mothers** | **52** | **150** | **150** |

| TP 48  Section 9  Signature Page(s) | **Deleted:**  Dr. John Steytler  **Added:**  Dr. Julia Botha | Change of Clinical Monitor |
| --- | --- | --- |
| TP 48  Section 9  Signature Page(s) | **Deleted:**  Dr. Lynette Boshoff  **Added:**  Dr. Mark Hopley | Change of Medical Director |
| TP 48  Section 9  Signature Page(s) | **Deleted:**  Dr. Patrick Robinson  **Added:**  Dr. Jonathan Leith | Change of Team Member Medicine |
| TP 50  References | **Added:**  18. G Jourdain, N Ngo-Giang-Huong, P Tungyai, A Kummee, C Bowonwatanuwong , P Kantipong, P Lechanachai, S Hammer, M  Lallemant, and Perinatal HIV Prevention Trial Group : Exposure to Intrapartum Single-dose Nevirapine and Subsequent Maternal 6-Month Response to NNRTI-based Regimens. Abstract 41LB. 11th Conference on Retroviruses and Opportunistic Infections. San Francisco, CA Feb 2004. |  |
