# Supplementary material for: Efficacy of Short-Course AZT Plus 3TC to Reduce Nevirapine Resistance in the Prevention of Mother-to-Child HIV Transmission: A Randomized Clinical Trial
Source: PLoS Med. 2009 Oct 27;6(10):e1000172. doi: 10.1371/journal.pmed.1000172 (PMC2760761; doi:10.1371/journal.pmed.1000172)
Supplement: Text S8 — Protocol amendment 7. (0.25 MB DOC) [file pmed.1000172.s008.doc]

**Clinical Trial Protocol** **Amendment**

Boehringer Ingelheim (Pty), Ltd.

ABCD

| **Amendment Number:**  **Date:** | | | | 7 | | |  | | | |
| --- | --- | --- | --- | --- | --- | --- | --- | --- | --- | --- |
|  |  |  |  | 23 July 2004 | | |  | | |  |
| Trial No.: | | | 1100.1413 | | | ⌧ | | | Implemented only after documented approval of IRB / IEC | |
| Test Substance(s) | | | Nevirapine | | |  | | | Implemented immediately in order to eliminate immediate hazard IRB / IEC to be notified of change with request for approval | |
|  | | |  | | |  | | | Implemented immediately as changes involve only logistical or administrative aspects. IRB / IEC notified of changes only | |
| Title: | | | An Open-label Study evaluating the Resistance profile of Single dose Nevirapine (NVP) when combined with a 4 or 7 day course of Combivir® (ZDV/3TC) compared to Single dose Nevirapine for the Prevention of Mother to Child Transmission (pMTCT) of HIV - Treatment Options Preservation Study (T.O.P.S.) | | | | | | | |
| Changes: | | | Allow for interim analysis and include visit no 10 | | | | | | | |
| Reason For Change: | | Following amendment 6, which led to the cessation of enrollment into the single dose Nevirapine arm only, it was deemed necessary to perform a formal interim analysis on all patients who were entered into the trial until the implementation of amendment 6.  To extend the follow up period to 24 months for any patient who demonstrates genotypic resistance to nevirapine at 18 months  To describe the informal 6 monthly report as required by the South African Medicines Control Council | | | | | | | | |
|  | | | | |  | | | **Page 1 of 11** | | |
| Confidential | © Boehringer Ingelheim  This protocol is the property of Boehringer Ingelheim and may not - in full or in part - be passed on, reproduced, published or otherwise used without the express permission of Boehringer Ingelheim | | | | | | | | | |

# PROTOCOL AMENDMENT SIGNATURE PAGE

| **BI Trial No.:** | 1100.1413 | | |  | | |
| --- | --- | --- | --- | --- | --- | --- |
| **Amendment No.:** | 7 | | |  | | |
| Trial Clinical Monitor: | |  |  | |  |  |
| Name  Organisation/Department | |  | date | |  | Dr. Julia Botha  Boehringer Ingelheim (Pty) Ltd / Medical Dept. |
| Trial Statistician: (indicate early information on signature, if applicable) | |  |  | |  |  |
| Name  Organisation/Department | |  | date | |  | Mr. Toshio Kimura  Boehringer Ingelheim Pharmaceuticals, Inc./ Biometrics  and Data Management |
| Medical Director: | |  |  | |  |  |
| Name  Organisation/Department | |  | date | |  | Dr. Mark Hopley  Boehringer Ingelheim (Pty) Ltd / Medical Dept. |
| Team Member Medicine: (indicate early information on approval, if applicable) | |  |  | |  |  |
| Name  Organisation/Department | |  | date | |  | Dr. Jonathan Leith  Corporate Medical advisor Virology  Boehringer Ingelheim GmbH |
| I herewith certify that I agree to adhere to the amended trial protocol and to all documents referenced in the amended trial protocol. | | | | | | |
| Investigator: | |  |  | |  |  |
| Name | |  | date | |  |  |
| Organisation/Department | |  |  | |  |  |

| **Page**  **(Section Number)** | **Changes** | **Reason for Change** |
| --- | --- | --- |
| Amendment3  AM3  Flow chart and footnote changes | **See Attachment 1** | Flow charts have been amended to incorporate Visit 10 at 24 months |
| Amendment 1  AM3  Section 3.1  No. of subjects planned | Patient participation will be for 6 weeks unless resistance is demonstrated in which case patients will be followed up for 12 months. All patients demonstrating resistance after 12 months will be followed for a further 6 month period.  **Amended to:**  Patient participation will be for 6 weeks unless resistance is demonstrated in which case patients will be followed up for 24 months. | To provide longer follow-up of resistance |
| Amendment 1  AM4  Section 6.2  Study procedures at each visit, mothers | Visit 9: (final visit, day 504 ± 14 days)  Assess mother for any HIV related signs and symptoms  Perform a targeted physical examination  Record any adverse events and concomitant therapies  Perform laboratory tests for CD4, viral load, resistance and other HIV-1 virologic testing  This visit concludes participation in the study by the mother  **Amended to:**  Visit 9 (day 504 ±14 days)  Assess mother for any HIV related signs and symptoms  Perform a targeted physical examination  Record any adverse events and concomitant therapies  Perform laboratory tests for CD4, viral load, resistance and other HIV-1 virologic testing  This visit concludes participation in the study by the mother if there is no demonstrated genotypic resistance at this visit. | Visit 9 is no longer the final visit. |
| TP33  Section 6.2  Study procedures at each visit, mothers | **Added:**  Visit 10: (final visit, day 672 ± 14 days)  Assess mother for any HIV related signs and symptoms  Perform a targeted physical examination  Record any adverse events and concomitant therapies  Perform laboratory tests for CD4, viral load, resistance and other HIV-1 virologic testing  This visit concludes participation in the study by the mother. | Visit 10 allows for 24 month follow up of patients with genotypic resistance at 18 months. |
| Amendment 1  AM5  Section 6.2  Study procedures at each visit, infants | Visit 9 ( final visit, day 504 ± 14 days)  Perform a targeted physical examination on the infant  Assess the infant for HIV related signs and symptoms  Record any adverse events and all concomitant therapies in the infant  Record the method of infant feeding  Collect blood samples for viral load measurement (HIV RNA PCR) and resistance and other HIV-1 virologic testing  This visit concludes participation in the study by the infant  **Amended to:**  Visit 9 (day 504 ± 14 days)  Perform a targeted physical examination on the infant  Assess the infant for HIV related signs and symptoms  Record any adverse events and all concomitant therapies in the infant  Record the method of infant feeding  Collect blood samples for viral load measurement (HIV RNA PCR) and resistance and other HIV-1 virologic testing  This visit concludes participation in the study by the infant if there is no demonstrated genotypic resistance at this visit. | Visit 9 is no longer the final visit |
| TP 36  Section 6.2  Study procedures at each visit, infants | **Added:**  Visit 10: (final visit, day 672 ± 14 days)  Assess infant for any HIV related signs and symptoms  Perform a targeted physical examination  Record any adverse events and concomitant therapies  Perform laboratory tests, viral load, resistance and other HIV-1 virologic testing  This visit concludes participation in the study by the infant. | Visit 10 allows for 24 month follow up of patients with genotypic resistance at 18 months. |
| Amenment 6  AM10  Section 7.3.4 | 7.3.4 Interim analyses No further interim analyses are planned. An analysis was performed, following release of new information , which supported the importance of resistance in subsequent response to antiretroviral treatment following Viramune®-containing mother to child prevention therapy ^18^  **Amended to:**  An analysis was performed, following release of new information , which supported the importance of resistance in subsequent response to antiretroviral treatment following Viramune®-containing mother to child prevention therapy ^18^  A further interim analysis will be performed on all the mother-infant pairs who were entered into the trial prior to implementation of amendment 6 (which discontinued the enrolment into the single dose nevirapine only arm) and who have completed 6 weeks. This interim analysis will not be used for additional study design modifications.  Further 6 monthly informal assessements of resistance and efficacy data will be performed to provide updates to regulatory health authorities. These reports will not be used to re-design or change the study plan . | Formal interim analysis to look at patients entered into the trial prior to amendment 6, and to comply with regulatory authorities’ requirements. |

Attachment 1: Flow chart changes

MOTHER

| Visit Number | 1 | 1.1 | 2 | 3 | 3.1 ^12^ | 4 | **5^2, 3^** | **6**** | **7**** | **7.1** | **8^5, 8^**** | 9*** |  |
| --- | --- | --- | --- | --- | --- | --- | --- | --- | --- | --- | --- | --- | --- |
|  | Screening  (Prenatal) | Screening Eligi-  bility  Visit | Enrollment  (Labour and delivery) |  | Drug Account-ability Visit |  |  |  |  |  |  |  | End of trial |
| Day | ≥ 34 weeks gestation | >Gest. age 34 w to Day 0 | 0 | 1-2 | 8 (±1) | 14  (± 2) | 42  (± 6) | 90  (± 10) | 168  (± 10) | 252  (± 10) | 336  (± 14) | 504  (± 14) |  |
| Informed Consent | X |  |  |  |  |  |  |  |  |  |  |  |  |
| HIV ELISA Test | X^9^ |  |  |  |  |  |  |  |  |  |  |  |  |
| Demographics | X |  |  |  |  |  |  |  |  |  |  |  |  |
| Review Inclusion/ Exclusion criteria | X | X | X |  |  |  |  |  |  |  |  |  |  |
| Medical History | X |  |  |  |  |  |  |  |  |  |  |  |  |
| Randomisation |  |  | X |  |  |  |  |  |  |  |  |  |  |
| Post delivery history |  |  |  | X |  |  |  |  |  |  |  |  |  |
| HIV related symptoms and signs | X |  |  | X |  |  | X | X | X | X | X | X |  |
| Physical Examination | X |  |  | X |  |  | X | X | X | X | X | X |  |
| Viral load PCR (RNA) | X |  | X | X |  | X | X | X | X | X | X | X |  |
| CD4 | X |  | X | X |  | X | X | X | X | X | X | X |  |
| Laboratory Tests ^4^ | X |  | X | X |  | X | X |  |  |  |  |  |  |
| Labour and Delivery History |  |  | X |  |  |  |  |  |  |  |  |  |  |
| Adverse Events | X | X | X | X | X | X | X | X | X | X | X | X |  |
| Concomitant Therapy | X | X | X | X | X | X | X | X | X | X | X | X |  |
| Drug Administration |  |  | X^1^ | X^1^ |  |  |  |  |  |  |  |  |  |
| Drug accountability/ compliance |  |  | X | X | X | X |  |  |  |  |  |  |  |
| Sampling for Resistance testing |  |  | X | X |  | X | X | X | X | X^11^ | X | X |  |
| End of trial admin/trial completion |  |  |  |  |  |  |  |  |  |  |  |  | X^2,3,5,10^ |

Amended to:

MOTHER

| Visit Number | 1 | 1.1 | 2 | 3 | 3.1^11^ | 4 | **5^2, 3^** | **6**** | **7**** | **7.1**** | **8^5, 8^**** | 9*** | 10**** |  |
| --- | --- | --- | --- | --- | --- | --- | --- | --- | --- | --- | --- | --- | --- | --- |
|  | Screening  (Prenatal) | Screening Eligi-  bility  Visit | Enrollment  (Labour and delivery) |  | Drug Account-ability Visit |  |  |  |  |  |  |  |  | End of trial |
| Day | ≥ 34 weeks gestation | >Gest. age 34 w to Day 0 | 0 | 1-2 | 4 to 9 | 14  (± 2) | 42  (± 6) | 90  (± 10) | 168  (± 10) | 252  (± 10) | 336  (± 14) | 504  (± 14) | 672  (± 14) |  |
| Informed Consent | X |  |  |  |  |  |  |  |  |  |  |  |  |  |
| Demographics | X |  |  |  |  |  |  |  |  |  |  |  |  |  |
| Review Inclusion/ Exclusion criteria | X | X | X |  |  |  |  |  |  |  |  |  |  |  |
| Medical History | X |  |  |  |  |  |  |  |  |  |  |  |  |  |
| Randomisation |  |  | X |  |  |  |  |  |  |  |  |  |  |  |
| Post delivery history |  |  |  | X |  |  |  |  |  |  |  |  |  |  |
| HIV related symptoms and signs | X |  |  | X |  |  | X | X | X | X | X | X | X |  |
| Physical Examination | X |  |  | X |  |  | X | X | X | X | X | X | X |  |
| Viral load PCR (RNA) | X |  | X | X |  | X | X | X | X | X | X | X | X |  |
| CD4 | X |  | X | X |  | X | X | X | X | X | X | X | X |  |
| Laboratory Tests ^4^ | X |  | X | X |  | X | X |  |  |  |  |  |  |  |
| Labour and Delivery History |  |  | X |  |  |  |  |  |  |  |  |  |  |  |
| Adverse Events | X | X | X | X | X | X | X | X | X | X | X | X | X |  |
| Concomitant Therapy | X | X | X | X | X | X | X | X | X | X | X | X | X |  |
| Drug Administration |  |  | X^1^ | X^1^ |  |  |  |  |  |  |  |  |  |  |
| Drug accountability/ compliance |  |  | X | X | X | X |  |  |  |  |  |  |  |  |
| Sampling for Resistance testing |  |  | X | X |  | X | X | X | X | X^10^ | X | X | X |  |
| End of trial admin/trial completion |  |  |  |  |  |  |  |  |  |  |  |  |  | X^2,3,5,9^ |

INFANT

| **Visit Number** | **2** | **3** | **3.1^11^** | **4** | **5^2 ,3^** | **5.1*** | **6**** | **7**** | **7.1**** | **8^58^**** | **9***** |  |
| --- | --- | --- | --- | --- | --- | --- | --- | --- | --- | --- | --- | --- |
| **Day** | **0** | **1-2**  **(within 0-72 hrs)** | **4 to 9** | **14**  **(± 2)** | **42**  **(± 6)** | **49**  **(± 6)** | **90**  **(± 10)** | **168**  **(± 10)** | **252**  **(± 10)** | **336**  **(± 14)** | **504**  **(± 14)** | **End of Trial** |
| Neonatal History (Including Apgar score) |  | X |  |  |  |  |  |  |  |  |  |  |
| Patient Demographics |  | X |  |  |  |  |  |  |  |  |  |  |
| Eligibility criteria |  | X |  |  |  |  |  |  |  |  |  |  |
| Record infant feeding method |  | X |  | X | X |  | X | X | X | X | X |  |
| Physical Examination |  | X |  | X | X |  | X | X | X | X | X |  |
| HIV related symptoms and signs |  |  |  |  | X |  | X |  |  | X | X |  |
| Laboratory Tests^4^ |  | X |  | X | X |  |  |  |  |  |  |  |
| PCR (RNA) |  | X |  | X | X | X | X | X | X | X | X |  |
| PCR (DNA) |  | X^7^ |  | X | X² | X |  |  |  |  |  |  |
| Drug Administra-tion |  | X^1^, ^6^ |  |  |  |  |  |  |  |  |  |  |
| Drug Accountability/  compliance |  | X | X | X |  |  |  |  |  |  |  |  |
| Adverse Events |  | X | X | X | X |  | X | X | X | X | X |  |
| Concomitant Therapy |  | X | X | X | X |  | X | X | X | X | X |  |
| Sampling for Resistance testing |  | X |  | X | X | X | X | X | X^10^ | X | X |  |
| End of trial admin/trial completion |  |  |  |  |  |  |  |  |  |  |  | X^2,3,.5,9^ |

1. All mothers to receive a single dose of nevirapine in labour and will be randomised to either no Combivir® or 4 or 7 days of Combivir®, also to be administered while in labour. Infants to receive the same treatment as mother.
2. Visit 5 concludes patient participation if mother and infant do not demonstrate resistance or the infant remains HIV DNA PCR negative.
3. Visit 5 also to be completed, if possible, for all dropouts and withdrawals prior to visit 5.
4. Laboratory tests refer to: Full blood count, serum creatinine, AST, ALT, ALP, Total bilirubin, amylase.
5. Visit 8 concludes mother and infant participation in the trial for those mothers and infants with demonstrated resistance after visit 5, but with no demonstrated resistance at visit 8.

6. Retrovir® and 3TC® administered to infant within 24 hours after birth, nevirapine to be administered 24-72 hours after birth, or just prior to discharge if hospital stay is less than 24 hours.

7. Initial HIV DNA PCR to be performed within 48 hours.

8. Visit 8 also to be completed, if possible, for all dropouts and withdrawals between visits 5 to 8.

9. Visit 9 will conclude participation in the trial for all mothers and infants with demonstrated resistance at visit 8.

10. The sample will be stored from this visit, and will be tested for resistance only if the patient is lost to follow up after this visit, or if there was no detectable resistance at visit 8.

11.This visit is applicable only to those patients randomised to either the 4 or 7 day CBV arm.

* Extra visit for infants who test HIV DNA PCR positive for first time at visit 5.

****** Only for those patients with resistant virus.

******* Visit 9 is intended for those patients with demonstrated genotypic resistance at visit 8.

Amended to:

INFANT

| **Visit Number** | **2** | **3** | **3.1^11^** | **4** | **5^2 ,3^** | **5.1*** | **6**** | **7**** | **7.1**** | **8^58^**** | **9***** | **10^****^** |  |
| --- | --- | --- | --- | --- | --- | --- | --- | --- | --- | --- | --- | --- | --- |
| **Day** | **0** | **1-2**  **(within 0-72 hrs)** | **4 to 9** | **14**  **(± 2)** | **42**  **(± 6)** | **49**  **(± 6)** | **90**  **(± 10)** | **168**  **(± 10)** | **252**  **(± 10)** | **336**  **(± 14)** | **504**  **(± 14)** | **672**  **(± 14)** | **End of Trial** |
| Neonatal History (Including Apgar score) |  | X |  |  |  |  |  |  |  |  |  |  |  |
| Patient Demographics |  | X |  |  |  |  |  |  |  |  |  |  |  |
| Eligibility criteria |  | X |  |  |  |  |  |  |  |  |  |  |  |
| Record infant feeding method |  | X |  | X | X |  | X | X | X | X | X | X |  |
| Physical Examination |  | X |  | X | X |  | X | X | X | X | X | X |  |
| HIV related symptoms and signs |  |  |  |  | X |  | X |  |  | X | X | X |  |
| Laboratory Tests^4^ |  | X |  | X | X |  |  |  |  |  |  |  |  |
| PCR (RNA) |  | X |  | X | X | X | X | X | X | X | X | X |  |
| PCR (DNA) |  | X^7^ |  | X | X² | X |  |  |  |  |  |  |  |
| Drug Administra-tion |  | X^1^, ^6^ |  |  |  |  |  |  |  |  |  |  |  |
| Drug Accountability/  compliance |  | X | X | X |  |  |  |  |  |  |  |  |  |
| Adverse Events |  | X | X | X | X |  | X | X | X | X | X | X |  |
| Concomitant Therapy |  | X | X | X | X |  | X | X | X | X | X | X |  |
| Sampling for Resistance testing |  | X |  | X | X | X | X | X | X^10^ | X | X | X |  |
| End of trial admin/trial completion |  |  |  |  |  |  |  |  |  |  |  |  | X^2,3,.5,9^ |

1. All mothers to receive a single dose of nevirapine in labour and will be randomised to either 4 or 7 days of Combivir®, also to be administered while in labour. Infants to receive the same treatment as mother.
2. Visit 5 concludes patient participation if mother and infant do not demonstrate resistance or the infant remains HIV DNA PCR negative.
3. Visit 5 also to be completed, if possible, for all dropouts and withdrawals prior to visit 5.
4. Laboratory tests refer to: Full blood count, serum creatinine, AST, ALT, ALP, Total bilirubin, amylase.
5. Visit 8 concludes mother and infant participation in the trial for those mothers and infants with demonstrated resistance after visit 5, but with no demonstrated resistance at visit 8.

6. Retrovir® and 3TC® administered to infant within 24 hours after birth, nevirapine to be administered 24-72 hours after birth, or just prior to discharge if hospital stay is less than 24 hours.

7. Initial HIV DNA PCR to be performed within 48 hours.

8. Visit 8 also to be completed, if possible, for all dropouts and withdrawals between visits 5 to 8.

9. Visit 9 will conclude participation in the trial for all mothers and infants with demonstrated resistance at visit 8.

10. The sample will be stored from this visit, and will be tested for resistance only if the patient is to follow up after this visit, or if there was no detectable resistance at visit 8.

11.This visit is applicable only to those patients randomised to either the 4 or 7 day CBV arm.

* Extra visit for infants who test HIV DNA PCR positive for first time at visit 5.

****** Only for those patients with resistant virus.

*******Visit 9 is intended for those patients with demonstrated genotypic resistance at visit 8.

**** Visit 10 is intended for those patients with demonstrated genotypic resistance at Visit 9.
